# Cross-economy stability of predictor rankings for adolescent school belonging and mathematics anxiety: an explainable machine-learning analysis of PISA 2022

**DOI:** 10.3389/fpsyg.2026.1875261

**Published:** 2026-09-03

**Authors:** Yuan Liao, Qin Peng, Runlin Li

**Affiliations:** School of Intelligence Technology, Geely University of China, Chengdu, Sichuan, China

**Keywords:** cross-economy comparison, explainable machine learning, fixed-effects regression, mathematics anxiety, PISA 2022, questionnaire coverage, school belonging, TreeSHAP

## Abstract

**Introduction:**

School belonging and mathematics anxiety reflect distinct relational and motivational processes, but cross-economy comparisons can blur distinctions among psychological relationships, model-based importance, and questionnaire availability.

**Methods:**

We analyzed Program for International Student Assessment (PISA) 2022 data from 613,744 students; outcome-valid samples comprised 561,339 students in 78 economies for school belonging and 475,792 students in 76 economies for mathematics anxiety. Per-economy LightGBM models and TreeSHAP rankings estimated relative predictive salience. Secondary post-hoc analyses assessed economy-level predictor availability, excluded unadministered modules, and fitted complementary association models. Official individualism scores covered 74 economies; outcome-eligible reference sets covered 73 for belonging and 70 for anxiety.

**Results:**

Eight of 12 focal combinations retained stable relative predictive salience and formed two psychologically interpretable predictor patterns. Perceived school safety, exposure to bullying, and student-teacher relations formed a relational-institutional pattern for belonging, while mathematics self-efficacy, 21st-century-skills self-efficacy, achievement, growth mindset, and persistence formed an efficacy-achievement-engagement pattern for mathematics anxiety. In separate weighted economy fixed-effects samples of 241,786-476,803 students, safety (β = 0.323) and student-teacher relations (β = 0.282) were positively associated with belonging, exposure to bullying was negatively associated (β = 0.253), and the five focal mathematics predictors were negatively associated with anxiety (β = −0.080 to −0.340). Continuous individualism interactions were small (absolute β ≤ 0.047), inconsistent, and multilevel sensitivity models retained the direction of every main association. Four combinations involving stress resistance, assertiveness, and cooperation failed the availability criterion because these indices were much less available in low-individualism economies and unevenly distributed across IDV terciles.

**Discussion:**

The evidence therefore supported stable relative predictive salience across economies more strongly than individualism-related differentiation. Together, the predictive rankings and association models provided complementary evidence for these two patterns but did not establish psychological mechanisms, causal effects, or transferable intervention benefits.

## 1 Introduction

Interventions intended to strengthen adolescent school belonging and academic affect—including social-emotional-learning curricula and growth-mindset programs—are studied and implemented across diverse education systems, but whether their effects generalize across contexts remains an empirical question (Davies et al., 2025; Sidney et al., 2025; Macnamara and Burgoyne, 2023). Meta-analytic evidence shows that effect sizes vary substantially by sample composition, cultural context, and educational system (Macnamara and Burgoyne, 2023). The practical question motivating this study is therefore not whether PISA predictors establish intervention effects, but which predictors show cross-economy stable predictive salience after accounting for questionnaire availability and which require context-specific follow-up. The Program for International Student Assessment (PISA) 2022 cycle, with its harmonized items administered to over 600,000 15-year-olds in 80 economies, offers an opportunity to construct such a predictive map (OECD, 2023a, 2024).

School belonging—the subjective sense of being valued, included, and accepted by peers and teachers at one's school—is a foundational construct in educational psychology with deep theoretical roots in basic-needs theories of social motivation (Baumeister and Leary, 1995; Goodenow, 1993). Meta-analytic syntheses across thousands of studies have consistently linked school belonging to academic motivation, engagement, achievement, and mental health (Allen et al., 2018; Korpershoek et al., 2020; Allen, 2025). It is also a developmental and institutional outcome rather than a fixed student attribute: pupils' emotional engagement generally declines from late primary through secondary school, with the magnitude of that decline varying across systems (Jerrim and Kaye, 2026). PISA evidence on the pandemic likewise shows changes in school belonging, growth mindset, and exposure to bullying that vary by gender, migration background, and socioeconomic disadvantage (Quintero Peña et al., 2025). Recent multilevel analyses of PISA data have identified teacher–student relations and perceived school safety as relational conditions associated with belonging across countries (Elizondo-González et al., 2025; Palikara et al., 2025; Liu et al., 2024; Yin et al., 2024; Allen et al., 2024). Cross-cultural work has highlighted moderation by Hofstede's individualism and power-distance dimensions (Johnson et al., 2024; Ooi and Cortina, 2023), and a recent synthesis calls for cultural and contextual updating of belonging theory (Allen, 2025; Allen et al., 2025). The Integrative Framework of Belonging describes belonging as emerging from four interacting components—competencies, opportunities, motivations, and perceptions—across temporal, social, and environmental contexts (Allen et al., 2021). Perceived safety reflects students' appraisal of the school environment; exposure to bullying captures adverse relational experiences; and student–teacher relations represent opportunities for recognition and support at the interpersonal and institutional levels. They are conditions of students' school experience rather than personal skills. We use the framework to organize the interpretation of these predictors, not to test all of its proposed pathways.

Mathematics anxiety—the apprehension and tension experienced when engaging with mathematical tasks—is similarly a well-validated construct (Hopko et al., 2003) with consistent meta-analytic links to mathematics achievement (negative correlation around *r* = −0.29) (Barroso et al., 2021; Namkung et al., 2019; Caviola et al., 2022). Mathematics anxiety has both individual and contextual (peer-group) components: classroom-mean math anxiety predicts achievement above and beyond the individual level, with cross-country variation in the strength of the association (Lau et al., 2022). Bandura's self-efficacy account defines efficacy as a judgment of capability that can shape effort and persistence, rather than as a fixed personality trait (Bandura, 1977). Pekrun's control–value theory proposes that perceived control and value appraisals organize achievement emotions, including anxiety (Pekrun, 2006). These perspectives distinguish capability and control beliefs from responsibility, persistence, and other motivational or self-regulatory processes; the constructs should not be collapsed into a single undifferentiated class of “non-cognitive skills” (Jerrim, 2026). The PISA indices used here do not capture the full set of task-value appraisals or the mediating pathways proposed by these theories, so the study assesses whether the observed pattern is consistent with them rather than testing either account as a complete model. Mathematics motivation moderates the anxiety–achievement link, such that the negative association between anxiety and performance is weaker among more motivated students (Wang et al., 2015). Recent PISA 2022 evidence further documents heterogeneity in mathematics-anxiety correlates across digital learning environments, school cooperation climate, and parental involvement (Wang et al., 2025; Djekourmane et al., 2025).

The focal predictors therefore reflect several distinct psychological processes. MATHEFF and MATHEF21 are domain-specific self-efficacy beliefs: they concern perceived capability in mathematics rather than general self-worth. GROSAGR reflects a belief that ability can develop, whereas MATHPERS and PERSEVAGR reflect sustained effort or persistence after difficulty. These beliefs and behaviors may influence whether a demanding task is appraised as manageable, whether effort continues after an error, and whether physiological arousal is interpreted as a threat. STRESAGR concerns self-reported stress regulation, while COOPAGR and ASSERAGR describe interpersonal tendencies involved in coordinating with peers, voicing needs, and negotiating social situations. Their developmental meaning depends on classroom norms and relationship contexts; a higher score is not automatically adaptive in every setting. Mathematics achievement plausible values represent academic performance, not a psychological skill. This conceptual separation matters because a high SHAP rank for any of these variables identifies predictive information, not the psychological pathway that generated the association.

Together, these processes link the two outcomes to adolescent development, engagement, wellbeing, and inequality. A supportive relationship environment can satisfy relatedness needs and sustain emotional engagement, whereas exposure to bullying can undermine safety and participation. In mathematics, efficacy and control beliefs can shape threat appraisal and task engagement, while repeated success or failure can, in turn, reshape those beliefs. Social and economic disadvantage, migration experiences, gendered expectations, and unequal access to supportive teaching may condition each part of these reciprocal processes. The present analysis consequently treats student beliefs and social-emotional tendencies as situated within school and relationship environments, rather than as decontextualized attributes that alone explain adolescent outcomes.

A growing body of secondary-analysis research has applied machine-learning (ML) techniques to PISA data to predict academic and affective outcomes and identify their key correlates. Pooled-sample studies using gradient-boosted decision trees with TreeSHAP attributions have ranked features such as math self-efficacy, socioeconomic status, math anxiety, and disciplinary climate (Huang et al., 2024; Alvarez-Garcia et al., 2024; Öz et al., 2025; Zhu et al., 2025; Zhang and Cutumisu, 2024). Cross-national variants have used either per-cultural-cluster regression or pooled models with country fixed effects (Cheung et al., 2024; King et al., 2024; Alkan et al., 2025). Earlier-cycle ML analyses have also reported predictive heterogeneity across regions (Acıslı-Celik and Yesilkanat, 2023; Bayirli et al., 2023; San Martin Soares, 2024; Arroyo Resino et al., 2024). Among the PISA-ML studies reviewed for this study, we found no quantified per-feature typology distinguishing cross-economy stable from context-varying predictive importance. The closest analog (King et al., 2024) reported per-cultural-cluster ML models without an explicit per-feature cross-cultural classification. Existing PISA-ML papers have also noted that pooled designs do not adequately capture the economy's structure (Cheung et al., 2024; Huang et al., 2024), leaving an opening for analyses that combine per-economy attribution with cross-cluster aggregation.

The present study makes three related contributions. First, it identifies eight focal combinations that maintained stable relative predictive salience after the availability assessment and interprets them as a relational–institutional pattern for school belonging and an efficacy–achievement–engagement pattern for mathematics anxiety. Second, it complements predictive rankings with regression estimates of association direction and standardized magnitude. Third, it treats economy–feature availability as a prerequisite for interpreting apparent cultural differences, rather than as a secondary data-cleaning detail. The initial predictive analysis sorted features into four operational rank categories: cross-tercile stable, high-IDV-aligned, low-IDV-aligned, or not classified. These categories describe model-based rank patterns only; they do not denote context-invariant psychological mechanisms, cultural essences, or transferable intervention effects. The availability assessment and association models were formulated after the initial rank results were known and are therefore treated as *post-hoc* and exploratory rather than as confirmatory tests. We therefore separate three questions about predictive-rank stability, association direction and magnitude, and coverage-constrained cultural patterning.

### 1.1 Research questions and analysis chronology

The three research questions below organize the secondary analyses. They were formulated after the initial rank analysis and are therefore *post-hoc*, exploratory questions rather than prospectively formulated hypotheses.

- RQ1 (predictive stability). Among the 12 focal outcome–predictor combinations selected from the initial rank analysis, which variables maintain stable relative predictive salience after applying the economy–outcome–predictor availability rule across low-, mid-, and high-IDV economies?
- RQ2 (direction and magnitude). For the 12 focal outcome–predictor combinations, what are the directions and standardized magnitudes of the associations, and does continuous economy-level IDV moderate those associations after adjustment for student covariates and economy fixed effects?
- RQ3 (exploratory cultural pattern). After restricting comparisons to economies where a variable is usable, does any high-IDV-aligned predictive-rank pattern pass variable-specific permutation testing with false-discovery-rate correction and show a directionally consistent IDV interaction in the complementary association models?

Before the secondary analyses were developed, the initial rank analysis was motivated by five directional expectations: (H1) FEELSAFE, RELATST, and BULLIED would consistently be prominent predictors of school belonging; (H2) MATHEFF, mathematics achievement, and GROSAGR would be consistently prominent predictors of mathematics anxiety; (H3) growth mindset would rank more prominently in higher-IDV contexts; (H4) cooperation and peer-related variables would rank more prominently in lower-IDV contexts; and (H5) the belonging predictor structure would be more similar across cultural clusters than the mathematics-anxiety structure (Elizondo-González et al., 2025; Allen et al., 2018; Caviola et al., 2022; Barroso et al., 2021; Ooi and Cortina, 2023; Johnson et al., 2024). These expectations record the rationale for the initial analysis; they are not confirmatory hypotheses for subsequent availability or association analyses, and unsupported expectations are reported as such.

## 2 Materials and methods

### 2.1 Data

We use the PISA 2022 student questionnaire data file (CY08MSP_STU_QQQ.SAV), publicly released by the OECD (OECD, 2024, 2023a). The full file contains responses from 613,744 15-year-old students sampled within 80 participating economies under PISA's two-stage stratified probability-proportional-to-size sampling design (OECD, 2024; Mang et al., 2021). We use item-level data, OECD-derived weighted-likelihood-estimate (WLE) scale indices, final student weights, and 80 balanced-repeated-replication (BRR) replicate weights with Fay factor 0.5 as released.

Our documentation check identified three sources of missing values: student item non-response, planned item-level missingness resulting from the balanced incomplete block booklet design, and economy-level structural non-availability when a questionnaire module was not implemented or an index could not be derived. OECD international documentation and variable metadata were checked for construct definition, valid codes, scale direction, and module coverage; observed availability was then assessed separately for every economy, outcome, and predictor. Recent PISA case evidence shows that an apparently harmonized international item can still conceal economy-specific wording changes documented only in local materials (Jerrim et al., 2026). This check helps identify coding and coverage problems but cannot rule out all local questionnaire adaptations, which remains a limitation.

The primary predictive models are unweighted because the estimand is a within-sample, model-based ordering of feature importance for each economy, not a population mean, prevalence, or regression coefficient. Sampling weights remain essential for design-based population estimation in PISA; therefore, we use BRR weights for a descriptive comparison (Section 1) and include a final-student-weighted LightGBM/SHAP sensitivity analysis to assess whether unequal sampling probabilities alter the feature rankings or four-category assignments (Section 3.6; Supplementary Table S12).

Following the stated outcome-availability criterion, we excluded two economies (ISR, KSV) from belonging analyses with fewer than 500 non-missing rows; we excluded four (ISR, IDN, PHL, THA) from anxiety analyses for the same reason. The outcome-valid samples comprised 561,339 students in 78 economies for BELONG and 475,792 students in 76 economies for ANXMAT.

### 2.2 Outcomes

We focus on two affective constructs operationalized by OECD-released six-item WLE indices.

School belonging (BELONG) aggregates responses to six questionnaire items (ST034Q01TA–ST034Q06TA) measuring sense of being included and valued at school, scaled with the generalized partial credit model and trend-anchored to PISA 2018 (OECD, 2024). The released index was non-missing for 91.5% of students.

Mathematics anxiety (ANXMAT) aggregates six items (ST292Q01JA–ST292Q06JA) on tension and apprehension when doing mathematics, similarly GPCM-scaled and trend-anchored to PISA 2012, the last cycle in which mathematics was the major domain (OECD, 2024). The released index was non-missing for 77.5% of students.

### 2.3 Predictors

Seventy-three candidate predictors were assembled across eleven analytic blocks in the PISA 2022 international release: (1) demographics (gender ST004D01T, age, immigration status IMMIG, language at home LANGN, parents' ISCED, modal-grade GRADE); (2) socioeconomic resources (ESCS, HISEI, HOMEPOS, ICTRES, ICTHOME, BMMJ1, BFMJ2); (3) academic performance (first plausible values in mathematics, reading, and science: PV1MATH, PV1READ, PV1SCIE); (4) domain-specific motivational beliefs (MATHEFF, MATHEF21, GROSAGR); (5) social-emotional tendencies and self-regulation (ASSERAGR, COOPAGR, EMPATAGR, PERSEVAGR, STRESAGR, EMOCOAGR, CURIOAGR); (6) wellbeing and experience (EXPWB, LIFESAT, PSYCHSYM, BODYIMA, FEELLAH); (7) relational and school experiences (DISCLIM, BULLIED, RELATST, FEELSAFE, SCHRISK); (8) pedagogy and mathematics engagement (TEACHSUP, COGACMCO, COGACRCO, MATHPERS, EXPO21ST, EXPOFA, FAMCON); (9) attendance and engagement (SKIPPING, MISSSC); (10) information and communication technology (ICTAVHOM, ICTAVSCH, ICTOUT, ICTSCH, ICTQUAL, ICTSUBJ, ICTWKDY, ICTWKEND, ICTEFFIC, ICTENQ, ICTFEED, ICTINFO, ICTREG); and (11) family, parental, self-directed-learning, and creativity indices (FAMSUP, FAMSUPSL, CURSUPP, PARINVOL, SOCONPA, SOCCON, PASCHPOL, SDLEFF, PROBSELF, INFOSEEK, CREATEFF, CREATOPN, IMAGINE, OPENART). These blocks organize the analysis; they do not imply that all variables within a block form a single psychological construct. Supplementary Table S10 provides full predictor descriptions and coding directions. Economy code is treated as a grouping variable (not a predictor) and used for stratified cross-validation and per-economy attribution. The primary analysis uses PV1MATH, PV1READ, and PV1SCIE, with PV1–PV10 substitutions reported as a sensitivity analysis (Section 3.6; Supplementary Table S14).

Table 1 defines the psychological meaning, score direction, and analytic role of the 12 focal outcome–predictor combinations. In particular, BULLIED represents greater exposure to bullying at higher scores, and mathematics achievement uses PV1MATH in the predictive analysis but PV1MATH–PV10MATH in the complementary association analysis.

**Table 1:** Psychological meaning and score direction of the 12 focal outcome–predictor combinations.

Outcome	Code and measure	Psychological domain	Higher scores indicate	Measurement and interpretation not
Belonging	FEELSAFE: Feeling safe at school	Perceived school safety	Feeling safer at and around school	Student perception rather than an objective safety record.
Belonging	BULLIED: Exposure to bullying	Peer threat and victimization	More frequent exposure to bullying	Higher scores indicate more frequent exposure to bullying.
Belonging	RELATST: Student–teacher relationships	Interpersonal and institutional support	Better perceived student–teacher relationships	Student-reported relationship quality rather than a measure of overall teacher effectiveness.
Belonging	STRESAGR: Stress resistance	Self-regulation	Greater reported ability to remain calm and resist stress	Coverage-limited exploratory combination; inference applies only where the module was administered.
Belonging	ASSERAGR: Assertiveness	Interpersonal social-emotional tendency	Greater reported initiative and assertive expression	Coverage-limited exploratory combination; higher scores are not assumed beneficial in every context.
Belonging	COOPAGR: Cooperation	Interpersonal social-emotional tendency	Greater self-reported cooperative tendency	Individual tendency rather than classroom climate; coverage-limited exploratory combination.
Math anxiety	MATHEFF: Mathematics self-efficacy: formal and applied mathematics	Domain-specific self-efficacy	Greater confidence in formal and applied mathematics tasks	Positive published WLE scores indicate greater confidence; no additional reversal is required.
Math anxiety	MATHEF21: Mathematics self-efficacy: reasoning and 21st-century skills	Domain-specific self-efficacy	Greater confidence in mathematical reasoning and related tasks	A capability belief rather than an objective test of these skills.
Math anxiety	PVMATH: Mathematics achievement	Cognitive achievement	Higher estimated mathematics proficiency	PV1MATH informs the focal SHAP rank; PV1MATH–PV10MATH estimates and variances are pooled for regression.
Math anxiety	GROSAGR: Growth mindset	Belief about ability development	Stronger belief that ability can develop	A belief measure, not evidence that ability changed or that an intervention worked.
Math anxiety	MATHPERS: Effort and persistence in mathematics	Mathematics engagement	More proactive effort and sustained engagement in mathematics	Mathematics-specific study behavior rather than a general fixed personality trait.
Math anxiety	STRESAGR: Stress resistance	Self-regulation	Greater reported ability to remain calm and resist stress	Coverage-limited exploratory combination estimated in a separate outcome-specific sample.

BELONG increases with stronger school belonging, whereas ANXMAT increases with greater mathematics anxiety. WLE denotes weighted likelihood estimate; reverse-keyed items were handled in the published OECD indices. SHAP ranks quantify relative predictive contribution but have no positive or negative direction. Fixed-effects coefficients are conditional associations from separate models and do not establish causal effects or mutually adjusted independent contributions. The 12 rows comprise eight combinations that passed the coverage rule and four coverage-limited exploratory combinations.

When the outcome of an analysis is BELONG, we omit ANXMAT from the predictor set, and vice versa, to avoid assigning trivially high importance to one strongly correlated affective index over the other. The predictor set is intentionally broad and retains eight ICT-engagement variables at varying levels of granularity (subject-related lesson use, weekday and weekend frequency, self-efficacy, inquiry-based use, feedback-via-ICT, information-seeking, and views of regulated ICT use). None of these eight variables enter the initial cross-tercile-stable or high-IDV-aligned groups in either outcome, and excluding them yields identical focal classifications.

### 2.4 Cluster scheme

We used individualism (IDV) and long-term-orientation (LTO) scores from The Culture Factor Group's official country-comparison page, which states that these two dimensions were updated on 16 October 2023 (Minkov and Kaasa, 2022; The Culture Factor Group, 2023). The page was accessed on 12 July 2026, and the exact economy mapping, access date, page hash, and source-country correspondence are retained with the analysis files. A direct official-page IDV score could be mapped to 74 of the 80 economies that occur in the PISA student file; BRN, KHM, KSV, MAC, PSE, and UZB had no corresponding score and were excluded from all IDV analyses rather than assigned regional estimates. The PISA scopes for Baku (QAZ) and the participating Ukrainian regions (QUR) were mapped to the official Azerbaijan and Ukraine country scores, respectively; this use of a country score for a subnational reporting scope is examined separately in a sensitivity analysis. IDV scores were divided into terciles containing 25 low-IDV, 25 mid-IDV, and 24 high-IDV economies. Economy membership is reported in Supplementary Table S11. We used LTO only as an alternative grouping sensitivity; three of the 74 mapped economies had no LTO value on the official page. We use these economy-level scores as an exploratory macro-level grouping framework rather than as causal treatments or measures of individual students' cultural orientations.

The Hofstede tradition and revised Minkov–Hofstede scores have been criticized on several grounds, including national-level generalization, conceptual overlap between dimensions, and ecological-fallacy concerns when economy scores are applied to individuals (e.g. Johnson et al., 2024). We retain IDV because the initial analytical expectations and exploratory RQ3 focus on the individualism–collectivism axis, but we treat all cultural comparisons as secondary and descriptive. LTO grouping and exclusion of the two subnational reporting scopes provide sensitivity checks; neither check identifies individualism as the source of a rank or slope difference.

### 2.5 Economy–feature availability and structural missingness

In the secondary availability assessment, we counted non-missing observations for every economy–outcome–predictor cell. An economy was outcome-eligible when at least 500 students had a non-missing outcome. Within an eligible economy, a predictor was defined as usable for that outcome only when it had at least 500 non-missing observations and was observed for at least 50% of students with a valid outcome. This yielded 11,680 assessed cells (80 economies × 2 outcomes × 73 predictors). We summarized the proportion of usable economies within low-, mid-, and high-IDV terciles. As a continuous check, Spearman correlations related IDV to the economy-level non-missing proportion and point-biserial correlations related IDV to the binary usable indicator. These diagnostics identify whether apparent cultural rank differences coincide with questionnaire availability.

We then repeated each per-economy LightGBM/TreeSHAP model using only predictors that met the economy–outcome usability rule. A module that was structurally unavailable in an economy was not imputed. Because the number of usable predictors could differ across economies, we converted mean absolute SHAP values to ranks among the available predictors and to within-economy percentile importance before aggregation. We compared each predictor only across economies in which it was usable. Variable-specific low- vs. high-IDV permutation tests used 1,000 shuffles when at least three usable economies were present in each contrasted group and were corrected across all estimable feature tests with the Benjamini–Hochberg procedure. For a focal cross-cluster interpretation, a predictor additionally had to be usable in at least 50% of outcome-eligible economies in each of the three IDV terciles. Focal availability and percentile summaries are reported in Supplementary Table S17; full feature-level availability and coverage-adjusted results are included in the machine-readable code-and-results supplement.

### 2.6 Statistical inference framework

For the initial SHAP rank analysis, uncertainty was assessed using (a) bootstrap 95% confidence intervals on per-cluster mean ranks (1,000 resamples, economy-level resampling); (b) permutation-null distributions of the difference in cluster mean ranks (1,000 random shuffles of the economy-cluster assignment); (c) Benjamini–Hochberg false-discovery-rate control at *q* = 0.05 over all candidate features with estimable tests; and (d) sensitivity replication under alternative thresholds, weighting choices, random seeds, plausible values, aggregation statistics, and cluster schemes. SHAP magnitudes are not standardized across economies before aggregation; instead, they are converted within each economy to ranks (1 = highest mean absolute SHAP value), so that cross-economy comparisons depend on relative importance order rather than the raw SHAP scale. Cluster-level rank aggregation uses the economy-level mean, with the median reported as an aggregation sensitivity because the two summaries respond differently to uneven rank distributions. Operational thresholds for the four-category rank classification are stated in Section 1, and sensitivity to alternative thresholds and aggregators is reported in Section 3.6. Inferential procedures for the complementary association models are described in Section 2.8.

### 2.7 Analysis overview

**Figure 1:**
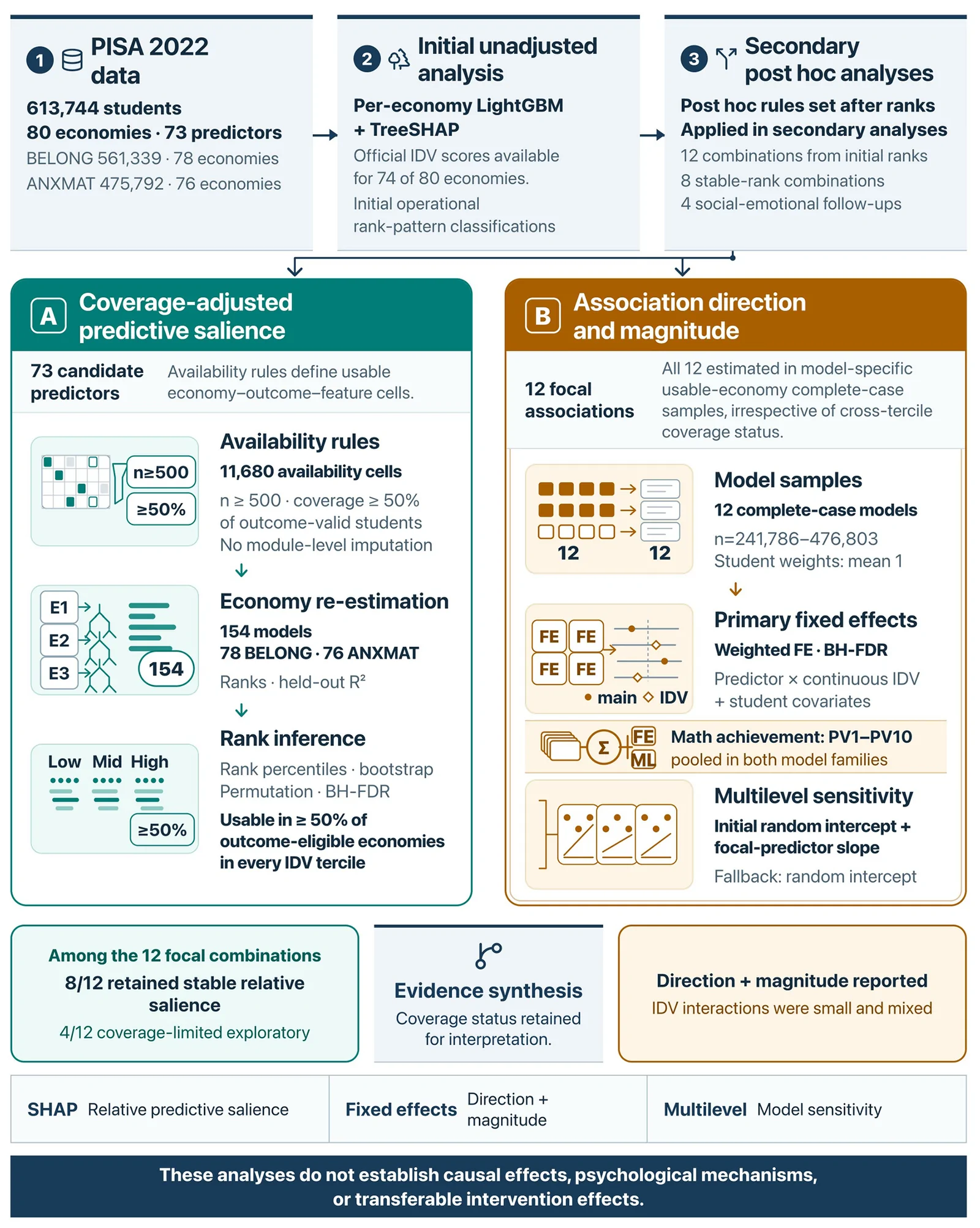
Analytical workflow. An initial unadjusted per-economy LightGBM/TreeSHAP rank analysis was followed by two secondary *post-hoc* branches: coverage-adjusted models assessing economy–feature availability, refitted models with usable predictors, and recomputed ranks; and weighted economy fixed-effects models with continuous individualism interactions, with multilevel models as sensitivity analyses. Interpretation integrates availability, rank stability, and association direction and magnitude; neither branch estimates causal effects.

Figure 1 summarizes the secondary coverage-adjusted and association workflow that followed the initial unadjusted rank analysis. PISA data and outcome-specific samples first undergo an economy–feature availability assessment. The predictive branch fits coverage-filtered per-economy LightGBM models, derives TreeSHAP ranks and percentiles, and applies IDV-tercile inference and the stated availability criterion. The association branch fits 12 separate weighted economy fixed-effects models with continuous IDV interactions and then evaluates multilevel-model sensitivity. The two branches distinguish relative predictive salience from association direction, standardized magnitude, and model-specification sensitivity. Because the workflow was formulated after the initial rank results were known, it is described as secondary and *post-hoc*.

Stage 1 (descriptive check). BRR-based economy-level weighted means and standard errors for BELONG and ANXMAT were computed using OECD's 80 BRR replicate weights with a Fay factor of 0.5 (OECD, 2024; Khorramdel et al., 2020). The OECD-37 weighted means were −0.09 (SD 0.97) for BELONG and +0.25 (SD 1.16) for ANXMAT; the positive ANXMAT mean is consistent with the post-pandemic increase in mathematics anxiety relative to PISA 2012 reported in OECD Volume II (OECD, 2023b). Per-economy BRR standard errors had medians 0.014 (BELONG) and 0.019 (ANXMAT), within plausible ranges for WLE-scaled indices.

Stage 2 (limited complete-case reliability and loading diagnostics). Because the balanced-incomplete-block design yielded very few students with responses to all six items, descriptive complete-case diagnostics could be calculated in only four economies with at least 30 complete cases for each scale. Across those four economies, Cronbach's α had a median of 0.78 (range 0.66–0.83) for BELONG and 0.85 (range 0.80–0.89) for ANXMAT. For each scale, 76 of 80 economies had fewer than 30 complete cases; 74 economies had no six-item complete case for BELONG and 75 had none for ANXMAT. We also examined single-factor principal-component loadings within the four eligible economies. These limited diagnostics describe internal consistency and loading patterns only; they are not formal tests of cross-economy measurement invariance, configural invariance, metric invariance, or alignment (Asparouhov and Muthén, 2014; Vandenberg and Lance, 2000; Ding et al., 2023; Sözer Boz, 2025).

Stages 3–4 (pooled linear and gradient-boosted comparators). For each outcome, we retained economies with at least 500 non-missing outcome rows. The linear comparator was ridge regression (RidgeCV over α∈{0.01, 0.1, 1, 10, 100}) with training-fold median imputation, standardization of continuous predictors, and one-hot encoding of categorical predictors and economy indicators. Because all coefficients, including the economy indicators, were ridge-penalized, this is not an unpenalized economy fixed-effects model. The pooled LightGBM comparator used native missing-data and categorical-feature handling (Ke et al., 2017; Borisov et al., 2024; Shwartz-Ziv and Armon, 2022; Chen and Guestrin, 2016; Arik and Pfister, 2021), included economy code as a categorical predictor, and used a learning rate of 0.05, 63 leaves, a minimum of 100 observations per leaf, feature and row subsampling fractions of 0.8, and no maximum-depth limit. Performance was estimated with ten student-level folds stratified by economy; this design preserves each economy's proportion in every fold and does not evaluate transfer to unseen economies. Within each outer LightGBM training fold, an economy-stratified 80%/20% fit/validation split selected the boosting iteration by early stopping. The model was then refitted for that fixed number of iterations on the complete outer training fold and evaluated only once on the untouched outer test fold. These pooled models provide a model-comparison benchmark rather than the per-economy attribution evidence used for the cross-economy conclusions. The choice of LightGBM over deep tabular networks (e.g., FT-Transformer, TabPFN Hollmann et al., 2025) was motivated by the empirical finding that gradient-boosted decision trees match or exceed deep tabular networks on heterogeneous tabular data of the size and structure of PISA (Borisov et al., 2024; Shwartz-Ziv and Armon, 2022).

Stage 5 (per-economy LightGBM with TreeSHAP). For each economy with ≥500 non-missing outcome rows, we fit an independent LightGBM regressor on a 64%/16%/20% train/validation/test split with early stopping on validation loss. These models used a learning rate of 0.05, 31 leaves, maximum depth 6, a minimum of 30 observations per leaf, and feature and row subsampling fractions of 0.8. Economy code was a grouping variable and was not supplied as a predictor. We extracted TreeSHAP attributions (Lundberg and Lee, 2017; Lundberg et al., 2020) on the entire per-economy sample using LightGBM's native pred_contrib=True mode and computed mean absolute SHAP per feature as the attribution magnitude. These attributions quantify each feature's contribution to fitted-model predictions under the observed data distribution. Held-out-sample *R*^2^ was reported per economy. We used the same split and hyperparameters when refitting the models after economy-specific feature-availability filtering.

Stage 6 (cluster aggregation). Per-economy mean absolute SHAP values were converted to within-economy feature ranks (1 = most important). We aggregated to the cluster level using the mean rank across economies within each cluster. The mean preserves information from the full rank distribution when economy-level ranks are uneven or multimodal; we report the median as an aggregation sensitivity. Bootstrap 95% confidence intervals on cluster mean rank were obtained from 1,000 economy-level resamples within each cluster.

Stage 7 (cross-cluster Spearman correlation and permutation null). Pairwise Spearman rank correlations on the union of each cluster's top-20 features were computed across cluster pairs. Permutation null distributions for these correlations were generated by 1,000 random shuffles of the economy-cluster assignment and recomputation of cluster mean ranks. Per-feature permutation *p*-values for the difference between low-IDV and high-IDV cluster mean ranks were also computed and BH-FDR-corrected across the 73 features.

Stage 8 (rank-pattern classification). Operational rules are: a feature is cross-tercile stable if its cluster mean rank is ≤ 15 in every cluster and the maximum CI width across clusters is ≤ 30; high-IDV-aligned if its cluster mean rank is ≤ 15 in the high-IDV cluster and ≥25 in the low-IDV cluster, with a BH-FDR-significant low-IDV-vs.-high-IDV difference; low-IDV-aligned under the corresponding reversed rule; and not classified otherwise. The rank-15 cutoff corresponds to approximately the top quintile of the 73-feature candidate set, while the rank-25 contrast criterion requires a feature to move clearly outside the top third in the comparison cluster. These thresholds are operational summaries of continuous rank evidence, not canonical natural cut-points. Sensitivity to these thresholds, to percentile-normalized thresholds, and to a stricter alternative rule set is reported in Section 3.6 and Supplementary Tables S6, S15.

Stage 9 (initial sensitivity analyses). The cluster-aggregation, cross-cluster, and rank-classification steps (Stages 6–8) are repeated under LTO terciles in place of IDV terciles, under multiple imputation of the predictor matrix for the ANXMAT outcome, after excluding the two subnational PISA reporting scopes QAZ and QUR that use corresponding country scores, and under alternative classification thresholds. Cohen's κ between primary and sensitivity classifications is computed for both the full four-category assignment and the cross-tercile-stable-vs.-other binary assignment.

Stage 10 (additional sensitivity analyses). Five further checks evaluate the stability of the typology: final-student-weighted LightGBM/SHAP models, five random seeds (42, 202, 2,022, 2,024, 777), PV1–PV10 plausible-value substitutions for achievement predictors, percentile-normalized thresholding, and a mean-vs.-median aggregation comparison. These analyses are reported in Section 3.6 and Supplementary Tables S12–S16.

### 2.8 Complementary association models

To provide direction and standardized magnitude that mean absolute SHAP rankings cannot, we estimated 12 focal outcome–predictor associations selected before the availability assessment. For BELONG, the focal predictors were FEELSAFE, BULLIED, RELATST, STRESAGR, ASSERAGR, and COOPAGR. For ANXMAT, the focal predictors were MATHEFF, MATHEF21, mathematics achievement (PV1MATH–PV10MATH), GROSAGR, MATHPERS, and STRESAGR. The first eight represented stable rank findings. The remaining four represented the social-emotional combinations highlighted by the preliminary high-IDV rank comparison; after restricting the cultural analysis to official IDV scores, COOPAGR no longer met the high-IDV-aligned rule, but it was retained to transparently evaluate the previously highlighted pattern. Coverage status was evaluated independently of the regression estimates.

Each focal predictor was fitted in a separate complete-case model rather than placing all 12 variables into a common model. This preserved the available sample for each questionnaire module and avoided conditioning every estimate on the intersection of structurally different modules. All models adjusted for ESCS, age, gender, immigration background, grade, and economy fixed effects. A structurally unavailable variable was not imputed. The resulting coefficients are conditional associations from separate models, not mutually adjusted direct effects and not causal estimates.

For the primary association analysis, the PISA final student weight was normalized to mean 1 within each model's economy-specific sample. These normalized weights were used both to standardize the outcome, focal continuous predictor, ESCS, and age within economy and to fit the weighted least-squares model. Continuous IDV was standardized once across the 74 economies with official scores. Model-specific regression samples ranged from 241,786 to 476,803 students across 34–67 economies. The model was specified as follows (Equation 1):


$$\begin{array}{c} Y_{ic}^{z}=\beta_{1}X_{ic}^{z}+\beta_{2}\left(X_{ic}^{z}\times \text{ID}{\text{V}}_{c}^{z}\right)\\ +\gamma^{T}C_{ic}+\alpha_{c}+\varepsilon_{ic}. \end{array}$$ (1)

Here, *i* indexes students and *c* indexes economies. $Y_{ic}^{z}$ is the outcome standardized within economy, and $X_{ic}^{z}$ is the focal predictor standardized within economy; both use the normalized final student weights. $\text{ID}{\text{V}}_{c}^{z}$ is the official economy-level individualism score standardized with equal economy weight across the 74 economies with available scores. ***C***_*ic*_ contains standardized ESCS and age together with indicator terms for gender, immigration background, and grade. β_1_ is the focal-predictor association at the mean level of IDV, β_2_ is the change in that association for a one-standard-deviation increase in IDV, **γ** contains the covariate coefficients, α_*c*_ denotes economy fixed effects, and ε_*ic*_ is the student-level residual. The IDV main effect is omitted because it is perfectly collinear with the economy fixed effects. Standard errors were clustered by economy, and finite-cluster inference used the number of economies minus one as the reference degrees of freedom. For mathematics achievement, we fitted the same model to PV1MATH through PV10MATH on an identical analysis sample; we combined estimates and within- and between-plausible-value variance using the standard multiple-imputation pooling rule with a Barnard–Rubin degrees-of-freedom adjustment. Within each outcome and modeling framework, the six focal main-effect tests and six *X*×IDV interaction tests formed separate Benjamini–Hochberg false-discovery-rate families.

As a model sensitivity analysis, we fitted unweighted linear mixed models to the same preprocessed samples, with economy random intercepts and focal-predictor random slopes. PISA weights were used to define the within-economy preprocessing but were not passed to lmer as prior weights. When the initial random-slope model was singular or failed to converge, the fallback rule retained an economy random intercept; we recorded all fallback decisions and final diagnostics. The ten mathematics plausible-value fits used a common final random-effects structure before pooling. These mixed models assess sensitivity to partial pooling and random-slope specification; they are not a complete complex-sampling variance analysis. Supplementary Table S18 reports estimates from both modeling frameworks and the final multilevel model status; detailed diagnostics are included in the machine-readable code-and-results supplement.

## 3 Results

### 3.1 Analysis samples and predictive performance

**Figure 2:**
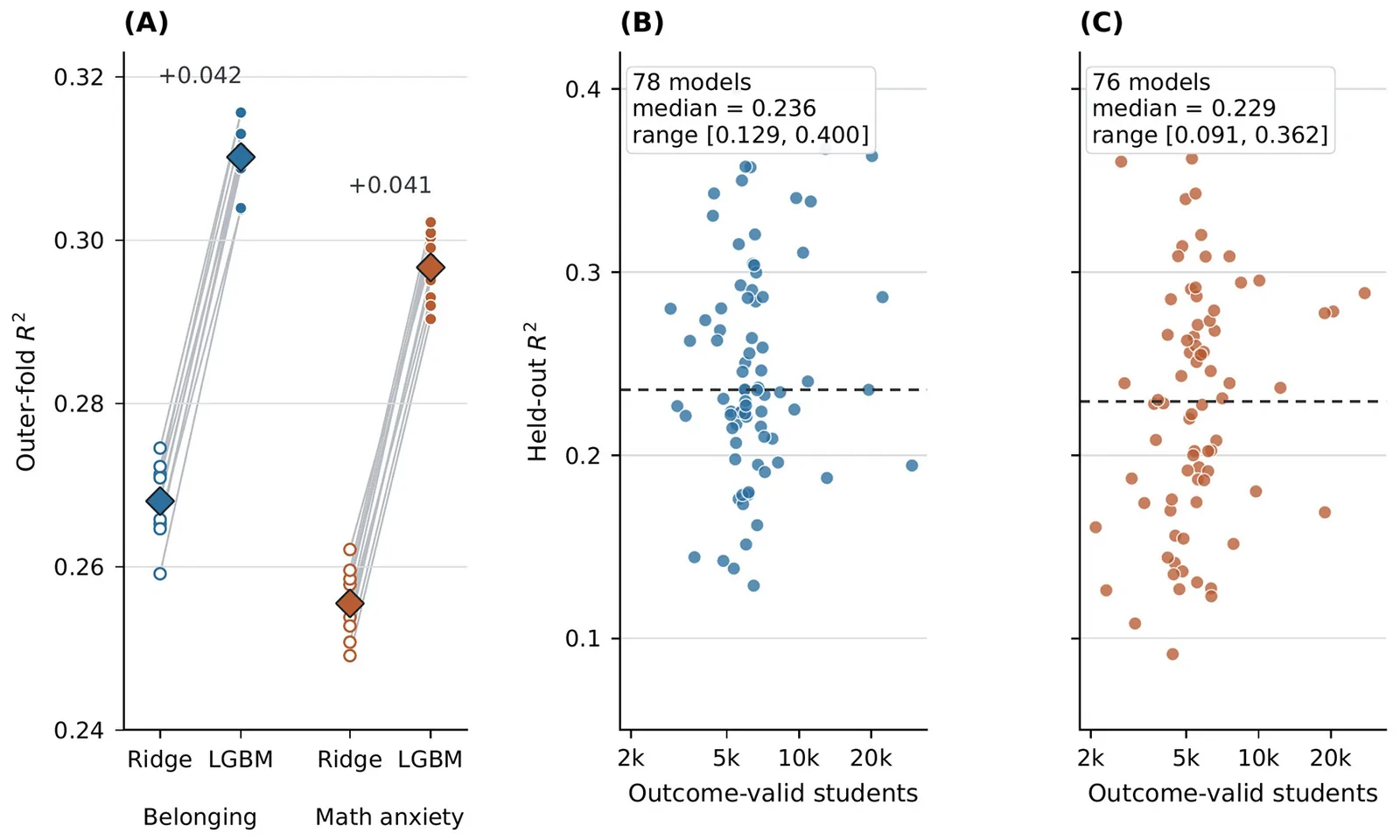
Predictive performance in pooled and economy-specific evaluations. **(A)** Links Ridge and LightGBM results from the same outer test fold for each outcome. The pooled comparison uses economy-stratified student-level outer test folds and does not evaluate transfer to unseen economies. **(B, C)** Show held-out *R*^2^ for the 78 BELONG and 76 ANXMAT coverage-adjusted economy-specific models, respectively; each point represents one economy.

Table 2 separates the source data, outcome-valid samples, economy-specific prediction models, official-IDV comparison set, and model-specific association samples. In the pooled comparison evaluated on untouched outer test folds, LightGBM produced higher *R*^2^ than ridge regression with penalized economy indicators: 0.310 vs. 0.268 for BELONG and 0.297 vs. 0.255 for ANXMAT. The coverage-adjusted economy-specific models had median held-out *R*^2^ values of 0.236 (range 0.129–0.400) for BELONG and 0.229 (range 0.091–0.362) for ANXMAT. Figure 2 shows both the paired pooled-fold comparison and the distribution of held-out performance across economy-specific models.

**Table 2:** Analytic samples, model counts, and out-of-sample predictive performance.

Panel A. Analytic samples and model counts			
Analysis unit	Students, *N*		Economies, *G*		Models or fitted units
PISA 2022 source data	613,744		80		–
BELONG outcome-valid sample	561,339		78		78 economy-specific samples
ANXMAT outcome-valid sample	475,792		76		76 economy-specific samples
Coverage-adjusted prediction (BELONG / ANXMAT)	–		78 / 76		154 outcome–economy models
Official individualism reference set	–		74		–
Weighted economy fixed-effects associations	241,786–476,803		34–67		12 summaries; 21 variable–PV fits
Multilevel sensitivity analysis	241,786–476,803		34–67		12 summaries; 21 variable–PV fits; 6 fallbacks
**Panel B. Out-of-sample predictive performance**					
**Outcome**	**Model and evaluation**	*N*	*G*	**Units**	*R*^2^ **summary**
Belonging	Ridge, Pooled 10-fold CV	561,339	78	10 folds	Mean 0.268 (SD 0.005); range [0.259, 0.275]
Belonging	LightGBM, Pooled 10-fold CV	561,339	78	10 folds	Mean 0.310 (SD 0.004); range [0.304, 0.316]
Belonging	Coverage-adjusted LightGBM, Per economy	561,339	78	78 models	Mean 0.247 (SD 0.060); median 0.236; range [0.129, 0.400]
Math anxiety	Ridge, Pooled 10-fold CV	475,792	76	10 folds	Mean 0.255 (SD 0.004); range [0.249, 0.262]
Math anxiety	LightGBM, Pooled 10-fold CV	475,792	76	10 folds	Mean 0.297 (SD 0.004); range [0.290, 0.302]
Math anxiety	Coverage-adjusted LightGBM, Per economy	475,792	76	76 models	Mean 0.226 (SD 0.065); median 0.229; range [0.091, 0.362]

The source-data count is not used in place of outcome-specific or model-specific samples. The 154 per-economy models comprise 78 BELONG and 76 ANXMAT models; students can contribute to both outcomes, so those outcome samples are not added. The official individualism reference set is economy-level and has no separate student count. Fixed-effects and multilevel results each summarize 12 focal combinations built from 21 variable–plausible-value fits; six multilevel fits required the stated fallback from a random-slope to a random-intercept model. Fixed-effects samples vary with focal-variable and covariate completeness. Pooled cross-validation used ten outer test folds stratified by economy. Bracketed values are the observed range across folds or economy models, not confidence intervals. Per-economy *R*^2^ values are independent held-out estimates.

### 3.2 Economy–feature availability

**Figure 3:**
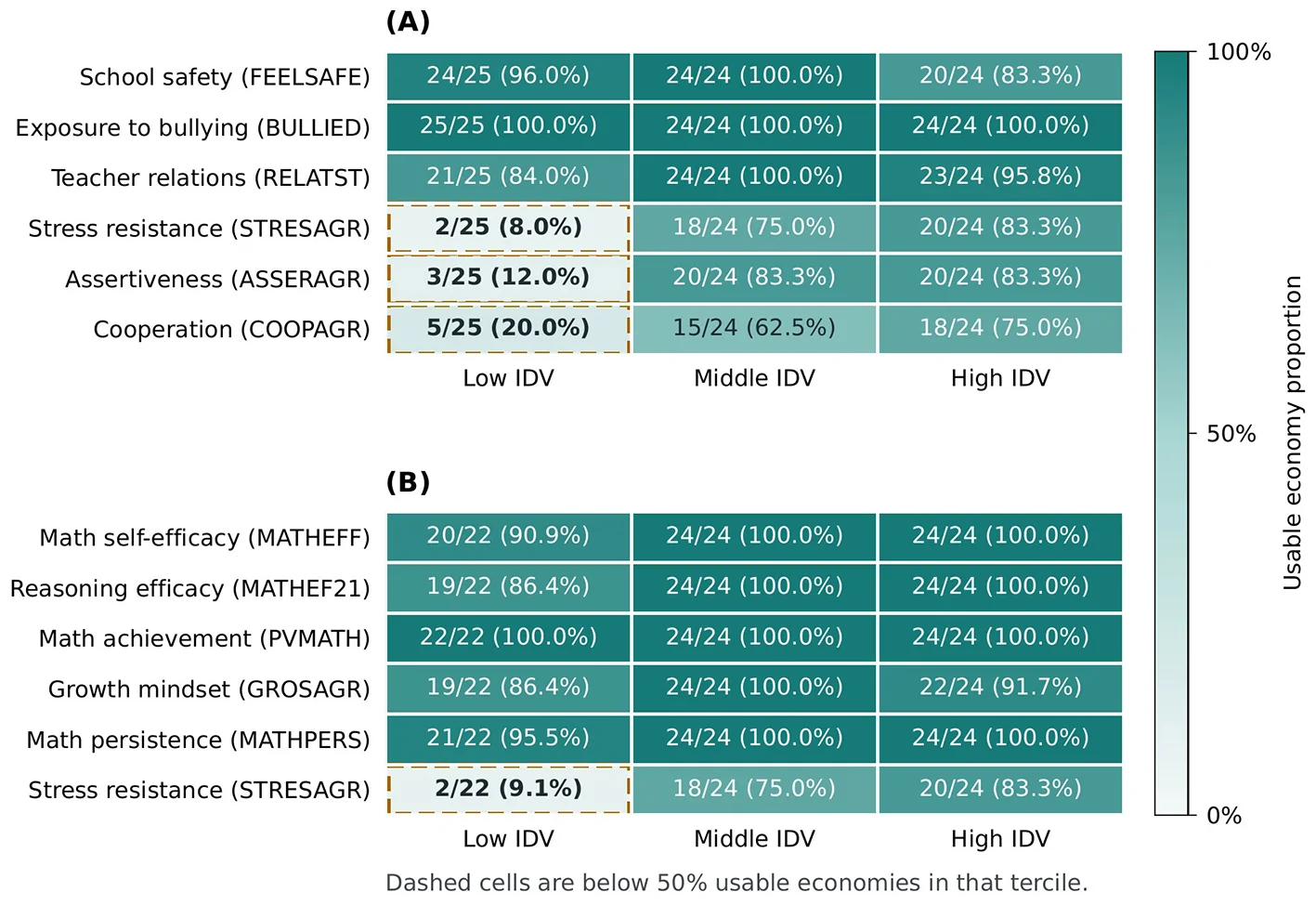
Availability of the 12 focal outcome–predictor combinations across individualism terciles. Each cell reports the number of usable economies divided by the number of outcome-eligible economies with an official IDV score in that tercile, followed by the corresponding percentage. An economy–feature cell was usable when it contained at least 500 non-missing students and at least 50% of that economy's outcome-valid sample. A focal cross-tercile interpretation additionally required usability in at least 50% of the outcome-eligible economies within every tercile. Dashed outlines identify the four combinations that failed this cross-tercile criterion. The mathematics-achievement row uses PV1MATH for the predictive analysis. **(A)** School belonging. **(B)** Mathematics anxiety.

The availability assessment covered 11,680 economy–outcome–predictor cells before applying the official-score restriction to cultural comparisons. The four social-emotional combinations selected for cultural follow-up showed pronounced imbalance across IDV terciles. Among outcome-eligible economies with official IDV scores, usable-economy coverage in the low-/mid-/high-IDV groups was 8%/75%/83% for BELONG–STRESAGR, 12%/83%/83% for BELONG–ASSERAGR, 20%/63%/75% for BELONG–COOPAGR, and 9%/75%/83% for ANXMAT–STRESAGR. Each failed the requirement of at least 50% usable-economy coverage in every tercile. By contrast, coverage for the eight focal combinations with stable relative predictive salience ranged from 83% to 100% in every outcome-relevant tercile. The four imbalanced combinations therefore cannot support a primary cross-cultural comparison and are retained only as coverage-limited exploratory results (Figure 3; Supplementary Table S17).

### 3.3 Coverage-adjusted predictive salience

After restricting each economy model to usable predictors, the median number of included variables was 57 for BELONG and 58 for ANXMAT. FEELSAFE ranked first among the available predictors in 56 of 78 belonging models, and MATHEFF ranked first in 39 of 76 mathematics-anxiety models.

Among the 12 focal outcome–predictor combinations, normalizing importance among the predictors available within each economy showed that FEELSAFE, BULLIED, and RELATST retained cross-cluster-stable relative predictive salience for school belonging. MATHEFF, MATHEF21, mathematics achievement, GROSAGR, and MATHPERS retained the corresponding status for mathematics anxiety (Table 3). BULLIED denotes exposure to bullying, with higher values indicating greater exposure; its mean absolute SHAP ranking indicates predictive salience but not whether the association is positive or negative.

**Table 3:** Coverage-adjusted predictive evidence for the 12 focal outcome–predictor combinations.

Outcome	Predictor	Usable economies, *n*/*N* (L/M/H)	Mean percentile (L/M/H)	High–low BH-FDR *q*	Interpretation
Belonging	FEELSAFE	24/25; 24/24; 20/24	0.99/0.99/1.00	0.256	Stable predictive rank
Belonging	BULLIED	25/25; 24/24; 24/24	0.95/0.96/0.98	0.068	Stable predictive rank
Belonging	RELATST	21/25; 24/24; 23/24	0.97/0.96/0.94	0.079	Stable predictive rank
Belonging	STRESAGR^†^	2/25; 18/24; 20/24	0.81/0.92/0.92	NE	Coverage-limited exploratory
Belonging	ASSERAGR^†^	3/25; 20/24; 20/24	0.92/0.93/0.92	0.837	Coverage-limited exploratory
Belonging	COOPAGR^†^	5/25; 15/24; 18/24	0.95/0.98/0.96	0.301	Coverage-limited exploratory
Math anxiety	MATHEFF	20/22; 24/24; 24/24	0.98/0.98/0.99	0.144	Stable predictive rank
Math anxiety	MATHEF21	19/22; 24/24; 24/24	0.97/0.97/0.97	0.452	Stable predictive rank
Math anxiety	PVMATH^‡^	22/22; 24/24; 24/24	0.88/0.90/0.94	0.057	Stable predictive rank
Math anxiety	GROSAGR	19/22; 24/24; 22/24	0.84/0.86/0.84	0.962	Stable predictive rank
Math anxiety	MATHPERS	21/22; 24/24; 24/24	0.95/0.92/0.81	0.023	Stable predictive rank
Math anxiety	STRESAGR^†^	2/22; 18/24; 20/24	0.99/0.98/0.98	NE	Coverage-limited exploratory

L/M/H denote low-, mid-, and high-individualism terciles. Each *n*/*N* is the number of usable economies divided by the number with a valid outcome in that tercile. A variable was usable when at least 500 students had non-missing data and they represented at least 50% of the economy's outcome-valid sample. The label Stable predictive rank required all three mean percentiles to be at least 0.806 and the largest bootstrap confidence-interval width to be at most 0.417; it does not require equal importance across terciles. The *q*-value separately tests the high-minus-low difference in mean percentile importance. The intended family comprised 73 candidate variables, and Benjamini–Hochberg correction was applied to the 68 estimable tests within each outcome. NE indicates that fewer than three low-tercile economies were estimable, not a non-significant result.^†^ At least one tercile failed the 50% economy-coverage rule; the combination remains exploratory regardless of its regression coefficient.^‡^ The predictive row uses PV1MATH and is labeled PVMATH to align with the ten-plausible-value regression summary.

After restricting the analysis to economies with official IDV scores and usable predictor data, the four coverage-limited focal combinations did not yield an interpretable high-IDV rank comparison. Only two low-IDV economies were usable for each STRESAGR comparison, so the requirement of at least three economies per contrasted group was not met, and no permutation *p* or FDR-adjusted *q*-value was computed. The available ASSERAGR and COOPAGR comparisons were not significant after BH-FDR correction (*q* = 0.837 and 0.301, respectively). Their mean percentile importance remained high in the economies where the modules were administered, but the low-IDV evidence was too sparse to support a cultural rank claim. The preliminary contrast cannot be separated from differential questionnaire availability and therefore does not support a stable cultural rank difference. No focal combination satisfied all three RQ3 requirements of balanced availability, FDR-corrected rank evidence, and a directionally consistent continuous-IDV interaction.

An exploratory scan of all 73 predictors identified additional rule-based classifications beyond the 12 focal combinations: EMOCOAGR met the coverage-adjusted stability rule for ANXMAT; OPENART and TEACHSUP met the high-IDV rank rules for BELONG and ANXMAT, respectively; and CURIOAGR met the low-IDV rank rule for BELONG. None was included in the complementary focal association models, so these classifications lack the interaction-direction check required by RQ3. They remain all-feature exploratory results in the machine-readable supplement and are not included in the primary conclusions.

**Figure 4:**
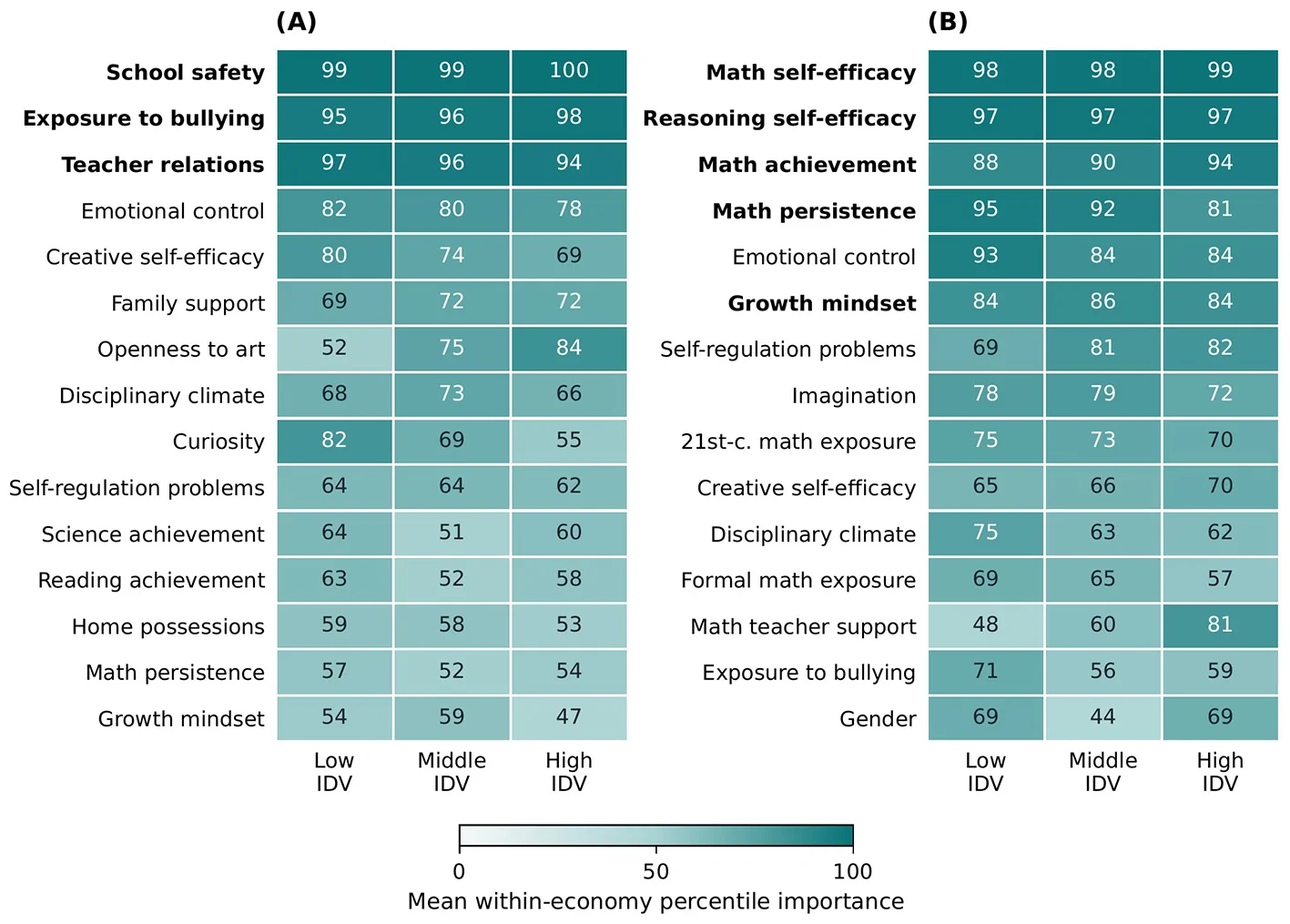
Coverage-adjusted predictive-importance landscape. For each outcome, the display includes the 15 coverage-eligible predictors with the highest equal-weighted average of the three IDV-tercile means. Cells show the mean within-economy percentile importance, with larger values indicating higher relative rank among the predictors available in that economy. Bold labels identify the eight focal combinations that retained stable relative predictive salience; a non-bold label does not, by itself, imply instability because the focal classification and this descriptive top-15 display answer different questions. Mean absolute SHAP does not indicate association direction or causal effects. **(A)** School belonging. **(B)** Mathematics anxiety.

**Figure 5:**
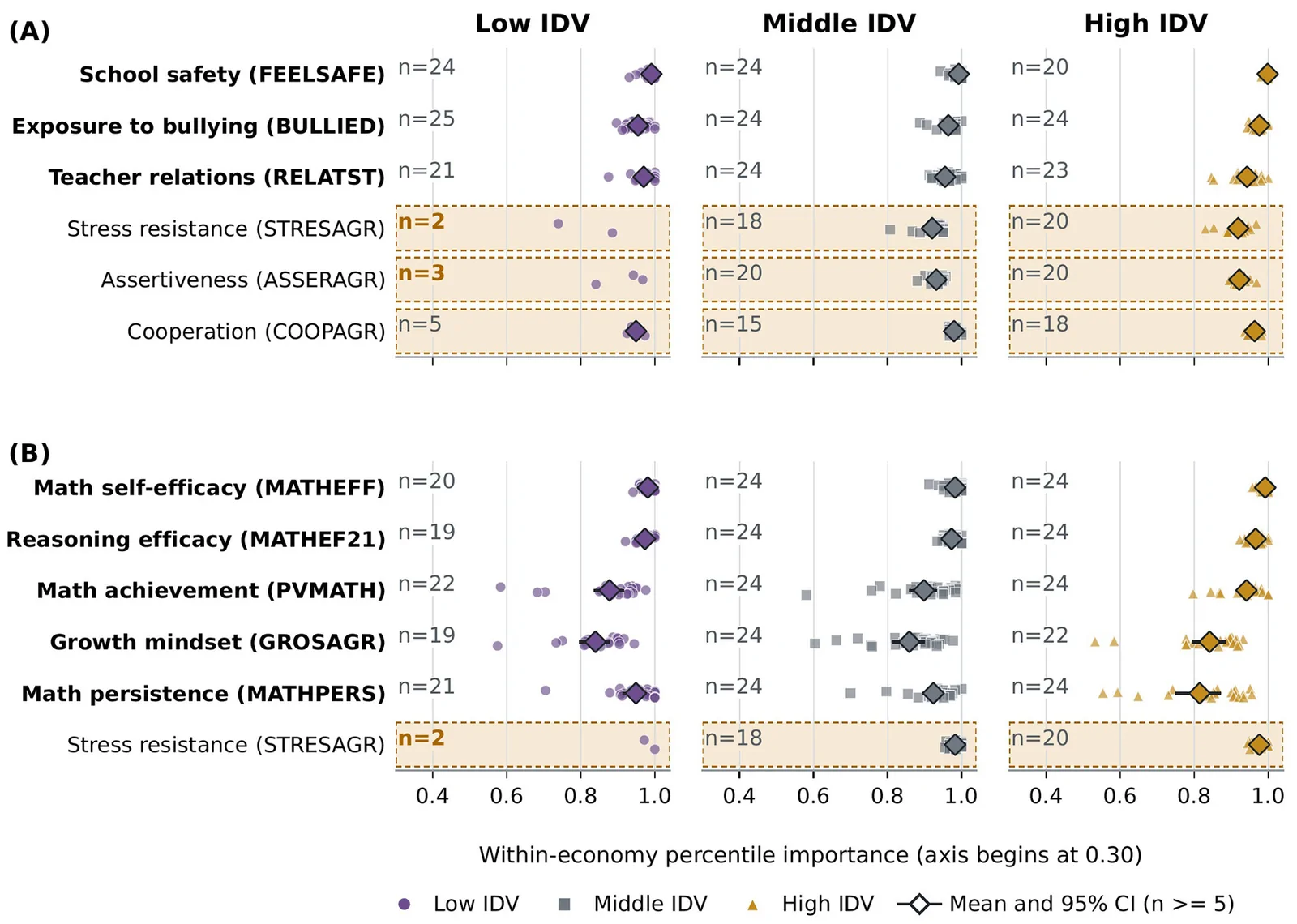
Economy-level distributions of coverage-adjusted percentile importance for the 12 focal combinations. Points represent usable economies; large markers and intervals show the IDV-tercile mean and the economy-bootstrap 95% confidence interval when at least five economies are available. Dashed rows identify coverage-limited combinations. Stable predictive rank means that relative importance remains high under the stated criteria; it does not require equal importance across IDV terciles. Mathematics achievement uses PV1MATH for the predictive analysis, and mean absolute SHAP does not indicate the sign of an association. **(A)** School belonging. **(B)** Mathematics anxiety.

Figure 4 places the focal findings within the broader coverage-eligible predictor landscape, and Figure 5 shows the economy-level distributions underlying the 12 focal summaries.

### 3.4 Association direction and magnitude

**Figure 6:**
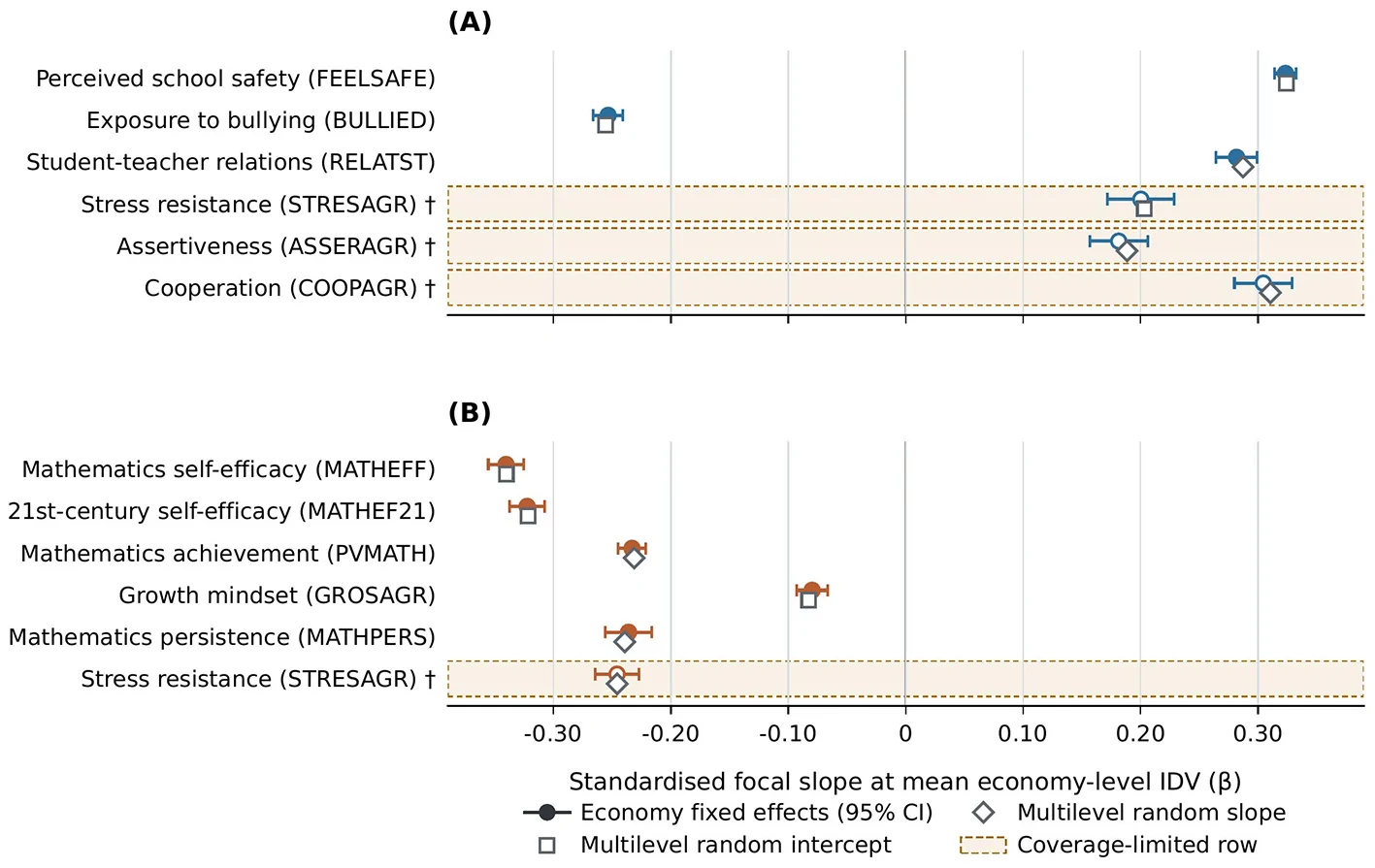
Standardized main associations for the 12 focal outcome–predictor combinations. Circles and horizontal lines show economy fixed-effects estimates and 95% economy-clustered confidence intervals; squares or diamonds show the corresponding multilevel sensitivity estimates. The fixed-effects estimates are the primary association results, whereas the multilevel points assess sensitivity to partial pooling and random-slope specification. Each predictor was fitted in a separate covariate-adjusted model. Mathematics achievement combines PV1MATH–PV10MATH. Shaded, dashed rows are coverage-limited and remain exploratory even when their within-sample association is precise. All estimates are observational associations, not causal effects. **(A)** School belonging. **(B)** Mathematics anxiety.

All 12 focal main associations survived outcome-specific BH-FDR correction (Table 4; Figure 6). For belonging, perceived school safety [β = 0.323, 95% CI [0.314, 0.333]] and student–teacher relations [β = 0.282, [0.264, 0.299]] were positively associated with the outcome, whereas greater exposure to bullying was negatively associated with belonging [β = −0.253, [-0.266, -0.241]]. For mathematics anxiety, higher mathematics self-efficacy (β = −0.340), 21st-century-skills self-efficacy (β = −0.322), mathematics achievement pooled across ten plausible values (β = −0.233), growth mindset (β = −0.080), and mathematics persistence (β = −0.236) were associated with lower anxiety. These coefficients provide direction and standardized magnitude, which mean absolute SHAP rankings alone cannot supply. Because each focal predictor was fitted separately, the coefficients are not mutually adjusted and should not be read as a ranking of independent contributions.

**Table 4:** Economy fixed-effects associations for the 12 focal outcome–predictor combinations.

Outcome	Predictor	Focal association β [95% CI]; *q*	Focal × IDV β [95% CI]; *q*	Students, *N*	Economies, *G*
Belonging	FEELSAFE	0.323 [0.314, 0.333]; *q* < 0.001	0.025 [0.015, 0.034]; *q* < 0.001	432,443	63
Belonging	BULLIED	–0.253 [–0.266, –0.241]; *q* < 0.001	–0.031 [–0.043, –0.018]; *q* < 0.001	476,803	67
Belonging	RELATST	0.282 [0.264, 0.299]; *q* < 0.001	–0.019 [–0.034, –0.004]; *q* = 0.017	427,031	62
Belonging	STRESAGR^†^	0.200 [0.172, 0.229]; *q* < 0.001	0.042 [0.011, 0.074]; *q* = 0.017	258,011	37
Belonging	ASSERAGR^†^	0.181 [0.157, 0.206]; *q* < 0.001	0.001 [–0.024, 0.025]; *q* = 0.956	274,137	40
Belonging	COOPAGR^†^	0.305 [0.280, 0.329]; *q* < 0.001	0.006 [–0.014, 0.026]; *q* = 0.685	246,041	34
Math anxiety	MATHEFF	–0.340 [–0.356, –0.325]; *q* < 0.001	–0.013 [–0.029, 0.003]; *q* = 0.146	369,638	62
Math anxiety	MATHEF21	–0.322 [–0.337, –0.307]; *q* < 0.001	–0.008 [–0.022, 0.006]; *q* = 0.282	377,227	61
Math anxiety	PVMATH	–0.233 [–0.245, –0.221]; *q* < 0.001	–0.033 [–0.050, –0.016]; *q* < 0.001	401,045	64
Math anxiety	GROSAGR	–0.080 [–0.093, –0.067]; *q* < 0.001	–0.028 [–0.044, –0.011]; *q* = 0.003	374,425	60
Math anxiety	MATHPERS	–0.236 [–0.256, –0.216]; *q* < 0.001	0.047 [0.026, 0.067]; *q* < 0.001	391,107	63
Math anxiety	STRESAGR^†^	–0.246 [–0.265, –0.227]; *q* < 0.001	–0.031 [–0.052, –0.010]; *q* = 0.007	241,786	37

IDV denotes economy-level individualism. Coefficients are standardized within-economy associations; the focal × IDV term gives the change in the focal association per one-economy-standard-deviation increase in IDV. Each focal predictor was fitted in a separate complete-case model with ESCS, age, gender, migration background, grade, and economy fixed effects; focal predictors were not mutually adjusted. Student weights were normalized to mean 1 within economy. Continuous student variables were weighted-standardized within economy, and IDV was standardized across the 74 economies with official scores. Standard errors were clustered by economy with a small-sample correction. Main associations and interactions were corrected separately within each outcome using the Benjamini–Hochberg false-discovery-rate procedure. The PVMATH row pools PV1MATH–PV10MATH estimates and variances.^†^ The combination failed the availability rule and remains a coverage-limited exploratory finding regardless of coefficient significance. These observational associations do not identify causal effects.

The IDV interactions were small in magnitude (|β| ≤ 0.047) and did not form a single moderation pattern. In the belonging models, the interaction was positive for FEELSAFE (β = 0.025, *q* < 0.001), negative for BULLIED (β = −0.031, *q* < 0.001), and slightly negative for RELATST (β = −0.019, *q* = 0.017). In the primary fixed-effects mathematics-anxiety models, the MATHEFF and MATHEF21 interactions did not survive FDR correction, whereas the interactions for mathematics achievement (β = −0.033, *q* < 0.001), GROSAGR (β = −0.028, *q* = 0.003), and MATHPERS (β = 0.047, *q* < 0.001) did. These terms describe variation in conditional association slopes; they do not establish psychological mechanisms attributable to IDV.

Coverage-limited models are also reported. In the economies where the measures were available, STRESAGR, ASSERAGR, and COOPAGR were positively associated with belonging (β = 0.200, 0.181, and 0.305, respectively), and STRESAGR was negatively associated with mathematics anxiety (β = −0.246). Each of the two STRESAGR models included only 37 economies and failed the cross-tercile availability criterion. Their interactions were statistically detectable, whereas the ASSERAGR and COOPAGR interactions were near zero and non-significant. Statistical significance within these selected samples does not restore any of the four coverage-limited results to primary status.

### 3.5 Multilevel model sensitivity

**Figure 7:**
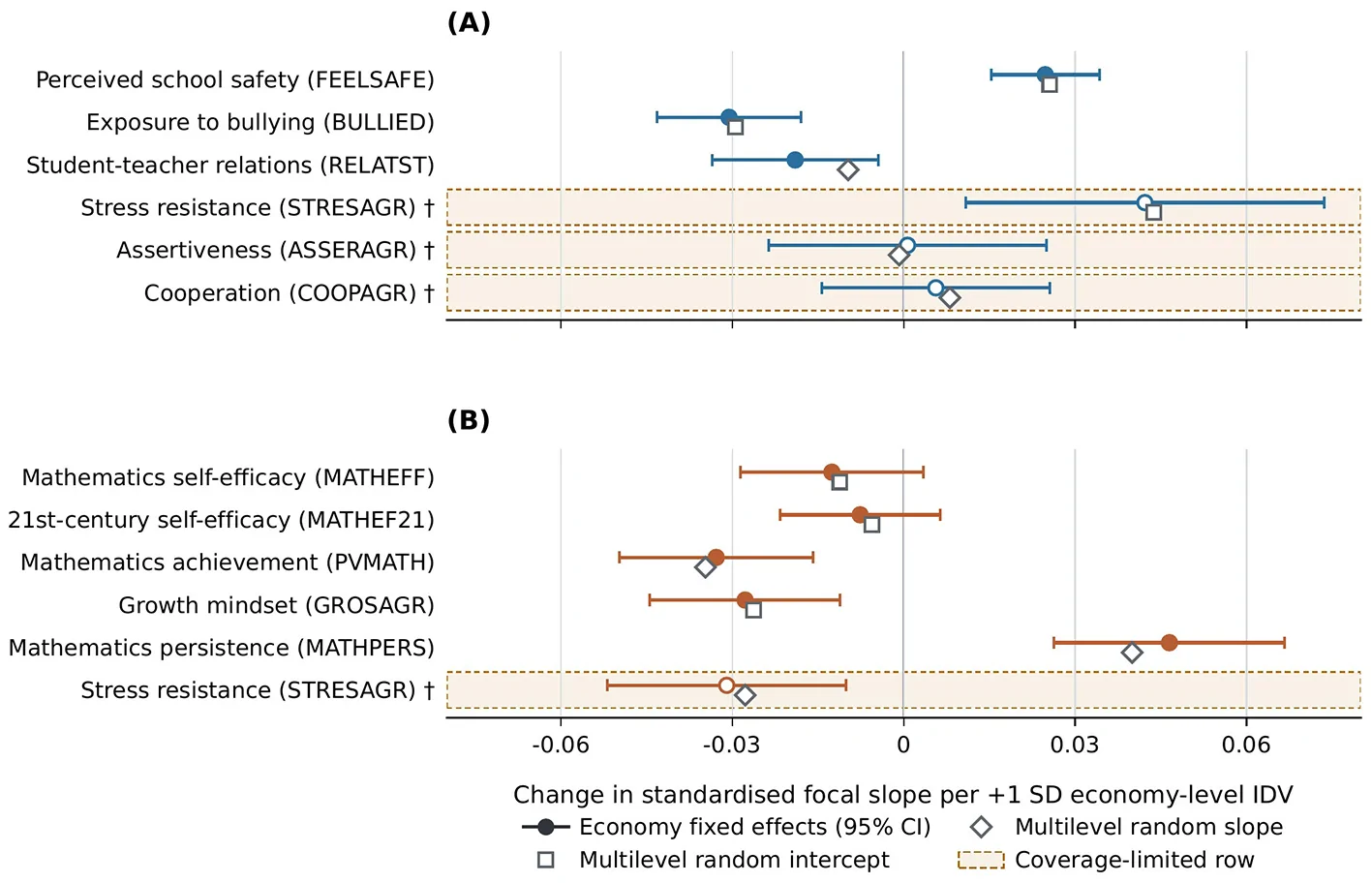
Continuous individualism interactions in the economy fixed-effects and multilevel models. Circles and horizontal lines show the primary fixed-effects interaction estimates and 95% economy-clustered confidence intervals; squares or diamonds show the multilevel sensitivity estimates. Each coefficient represents the change in the standardized focal-predictor slope associated with a one-standard-deviation increase in the official economy-level IDV. IDV is an economy-level measure rather than an individual cultural orientation. Agreement in direction does not imply identical estimates; the only sign difference concerns the near-zero, coverage-limited ASSERAGR interaction. The small, mixed-direction interactions do not establish a coherent cultural mechanism. **(A)** School belonging. **(B)** Mathematics anxiety.

The multilevel sensitivity estimates agreed in sign with the fixed-effects estimates for all 12 main associations (Figure 6) and for 11 of the 12 IDV interactions (Figure 7). The only sign discrepancy involved the near-zero, coverage-limited ASSERAGR interaction. Six of the 21 model/plausible-value fits used the random-intercept fallback after the initial random-slope model was singular; no final fit remained singular or failed. The fixed-effects models therefore remain the primary association analysis. Supplementary Table S18 reports the complete focal multilevel estimates and final model status; detailed diagnostics are included in the machine-readable code-and-results supplement.

### 3.6 Initial predictive-rank and sensitivity results

**Figure 8:**
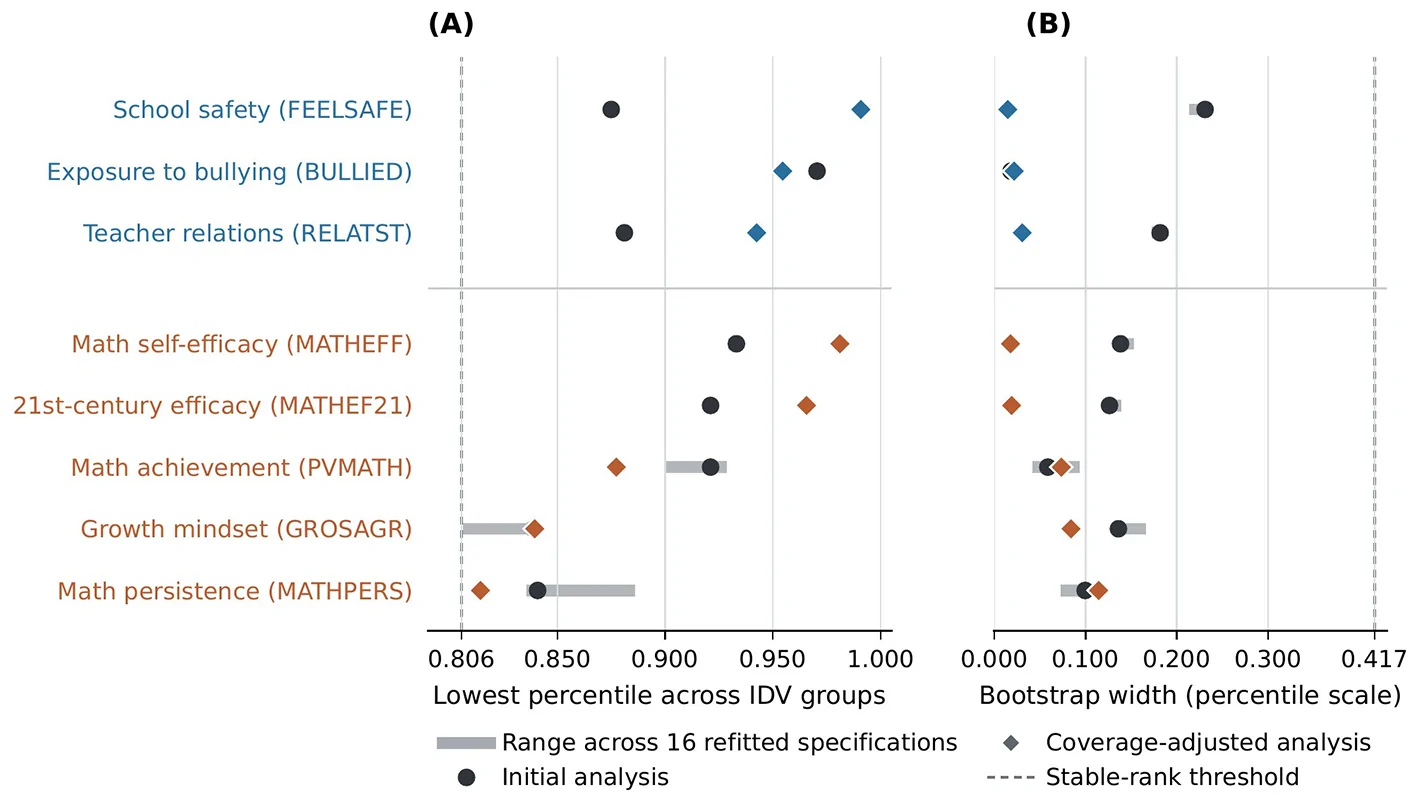
Predictive-rank sensitivity for the eight focal combinations with stable relative predictive salience. Panel A shows the minimum percentile importance across the three IDV terciles, and Panel B shows the maximum economy-bootstrap confidence-interval width. Gray ranges span five random-seed refits, ten mathematics plausible-value substitutions, and one final-student-weighted refit; these sixteen refitted specifications are sensitivity analyses, not independent replications. Filled circles show the initial unadjusted rank analysis with a fixed 73-predictor denominator, whereas colored diamonds show the coverage-adjusted analysis with each economy's actual number of usable predictors. Both are relative-rank summaries, so differences between these points should not be interpreted as changes in effect size. Dashed lines mark the stable-rank and interval-width criteria. Initial and coverage-adjusted mathematics-achievement points use PV1MATH; PV1MATH–PV10MATH are represented among the sensitivity refits. **(A)** Minimum percentile importance. **(B)** Rank uncertainty.

With the official IDV mapping, the unadjusted rank analysis placed the same eight focal combinations retained by the coverage-adjusted assessment in the cross-tercile-stable category. Among the four social-emotional follow-up combinations, BELONG–STRESAGR, BELONG–ASSERAGR, and ANXMAT–STRESAGR met the high-IDV-aligned rule, whereas BELONG–COOPAGR did not. Weighted, random-seed, plausible-value, percentile-normalized, and median-aggregation checks preserved all 12 focal assignments; non-focal assignments varied in some checks. Figure 8 summarizes the eight focal combinations with stable relative predictive salience across the 16 model-refitting specifications, while the threshold, aggregation, LTO, and imputation checks remain in Supplementary Tables. Grouping by LTO preserved the eight focal stable assignments but yielded no high-LTO-aligned feature. The student-level MICE analysis yielded Cohen's κ =.927 for the full classification and 0.902 for the stable-vs.-other comparison, but it could not address the non-availability of economy-level structural modules.

Of the five initial directional expectations, H1 and H2 were supported, whereas H3, H4, and H5 were not. GROSAGR did not rank more prominently in high-IDV economies (mean ranks 11.6/8.5/12.6 in the low-/mid-/high-IDV groups), and COOPAGR did not show the expected low-IDV prominence; its unequal availability further precludes a cultural interpretation. The high-vs.-low-IDV top-20 rank correlations were 0.070 for BELONG and 0.435 for ANXMAT, the opposite of the expectation that the belonging predictor structure would be more similar across cultural groups. These descriptive correlations and their permutation-null summaries are reported in Supplementary Table S5.

The initial unadjusted assignments, cross-cluster summaries, measurement and imputation checks, and analyses of thresholds, cluster schemes, weighting, seed, plausible values, and aggregation remain in Supplementary Tables S1, S5–S16 as a record of the initial analysis. The coverage-adjusted secondary assessment provides the basis for the primary interpretation of cross-economy rank stability.

## 4 Discussion

After accounting for questionnaire availability, eight focal combinations retained stable relative predictive salience and formed two psychologically interpretable patterns. Perceived safety, exposure to bullying, and student–teacher relations formed a relational–institutional pattern for school belonging. Mathematics self-efficacy, achievement, growth mindset, and sustained mathematics engagement formed an efficacy–achievement–engagement pattern for mathematics anxiety. Evidence for stable relative predictive salience across economies was stronger than evidence for individualism-related differentiation: stress resistance, cooperation, and assertiveness could not support a primary high-IDV conclusion because their questionnaire coverage was concentrated in higher-IDV economies.

The economy–feature availability assessment and complementary association models were secondary *post-hoc* analyses formulated after the initial rank results were known and are therefore interpreted as exploratory. The three analyses answer different questions. Coverage-adjusted SHAP reports a variable's relative contribution to predictions among the variables available in an economy. The economy fixed-effects models report the conditional direction and standardized magnitude of linear association. The multilevel models assess sensitivity to partial pooling and random-effects specification. These results are descriptive and do not identify a causal pathway, psychological mechanism, or intervention effect.

### 4.1 School belonging: relational and school-context correlates

Perceived safety and student–teacher relations were positively associated with belonging in the fixed-effects models (β = 0.323 and 0.282), whereas greater exposure to bullying was negatively associated (β = −0.253). The multilevel main-association estimates were very similar (0.324, 0.287, and −0.255, respectively). This convergence is consistent with the Integrative Framework of Belonging: safety and exposure to bullying speak to perceptions of relational value and threat, while teacher relationships provide interpersonal and institutional opportunities for recognition and reliable adult support (Allen et al., 2021). The models do not directly measure those mediating processes, so the framework offers a psychologically plausible interpretation rather than a demonstrated mechanism.

This relational interpretation also situates belonging within adolescent development and school institutions. Emotional engagement commonly declines during the transition to secondary school, though the magnitude of that decline varies across education systems (Jerrim and Kaye, 2026). Pandemic-era PISA comparisons likewise show that belonging and exposure to bullying changed differently by gender, migration background, and socioeconomic disadvantage (Quintero Peña et al., 2025). Safety, exposure to bullying, and teacher relationships should therefore not be read as interchangeable student “skills”: they describe the conditions under which adolescents experience inclusion, support, and threat. The small IDV interactions for FEELSAFE and BULLIED had the same direction in both modeling frameworks, but the RELATST interaction did not remain statistically distinguishable from zero in the multilevel model. Taken together, the interactions do not establish a general cultural moderation process.

### 4.2 Mathematics anxiety: efficacy, control, engagement, and achievement

Both mathematics self-efficacy indices showed substantial negative associations with anxiety (β = −0.340 and −0.322). In Bandura's account, self-efficacy is a domain-relevant judgement of capability that can shape effort and persistence (Bandura, 1977). Control–value theory places such control beliefs within the appraisal processes that organize achievement emotions (Pekrun, 2006). The negative associations are consistent with the control side of this account: difficult tasks may feel more manageable when students perceive greater capability, while repeated anxiety, success, and failure may in turn reshape perceived control. Because the study did not measure the complete task-value or mediating pathways, it does not test control–value theory as a full model. Even longitudinal observational evidence cannot by itself establish that control beliefs cause subsequent attainment (Jerrim, 2026).

Mathematics achievement and persistence were also negatively associated with anxiety (β = −0.233 and −0.236). The association with achievement may be reciprocal: anxiety may disrupt performance, while repeated difficulty may increase anxiety. MATHPERS represents mathematics effort and sustained engagement rather than a general personality characteristic. A growth mindset, the belief that ability can develop, showed a smaller negative association (β = −0.080); this does not mean mindset was the strongest mechanism, but that the belief provided stable predictive information and was associated with lower anxiety in its separate model. Because the five predictors were not entered together, their coefficients cannot be compared as independent psychological contributions.

The IDV interactions again did not form a coherent pattern. The mathematics-achievement and growth-mindset interactions were negative in both modeling frameworks, whereas the MATHPERS interaction was positive: its negative association with anxiety became modestly weaker as economy-level IDV increased. The fixed-effects interactions for MATHEFF and MATHEF21 did not survive FDR correction. These small, differently directed terms are better understood as exploratory slope variation rather than as evidence of a coherent IDV moderation process.

### 4.3 Coverage-limited social-emotional findings

Stress resistance, cooperation, and assertiveness require a different interpretation than the eight focal combinations that met the coverage and stable-rank criteria. The belonging models covered only 37, 34, and 40 economies, respectively, and the mathematics-anxiety STRESAGR model covered 37. Within those selected economies, greater stress resistance, cooperation, and assertiveness were associated with higher belonging, and greater stress resistance was associated with lower anxiety. These directions are consistent with self-regulation and interpersonal-coordination accounts but cannot be generalized to the full PISA economy set.

The coverage-adjusted rank comparisons provide an important qualification. In the economies where these indices were administered, their percentile importance was high and broadly similar across low-, mid-, and high-IDV groups. The ASSERAGR and COOPAGR interactions were near zero in both modeling frameworks. Although the STRESAGR interactions were statistically detectable, significance across 37 economies cannot compensate for the failed availability criterion. The evidence therefore does not support a high-IDV psychological mechanism. Cooperation and assertiveness remain interpersonal tendencies whose meaning depends on classroom norms, while stress resistance is a self-regulatory report whose measurement and relevance require local validation (Ooi and Cortina, 2023; Johnson et al., 2024).

The initial top-20 rank correlations may reflect a mix of relationship-context differences, measurement non-equivalence, and uneven questionnaire implementation. They describe predictor-structure similarity but cannot determine whether the underlying constructs are cross-culturally invariant. They are not used as a primary psychological conclusion.

### 4.4 Implications for local assessment and future intervention research

These implications identify priorities for local assessment and prospective testing; they are not instructions for deploying an intervention based on observational coefficients. School climate and belonging. Perceived safety, exposure to bullying, and student–teacher relations may be considered together because they represent complementary aspects of adolescents' relational environment. Schools could use locally validated climate surveys, transition-period check-ins, and student consultations to identify where students feel unsafe, excluded, or unsupported. Local pilot studies could test strategies to strengthen reliable adult relationships and peer inclusion when emotional engagement declines during the transition to secondary school (Jerrim and Kaye, 2026). Local analyses should also examine whether gender, migration experience, and socioeconomic disadvantage shape access to those relational supports (Quintero Peña et al., 2025).

Bullying prevention and student support: The BULLIED result reflects greater exposure to bullying, not a student-level protective skill. Its negative association with belonging warrants examination of school-level conditions, including supervision, confidential reporting channels, peer norms, restorative responses, and access to trusted adults, all of which require direct evaluation. Schools could assess whether existing safeguarding and referral procedures provide timely, supportive responses to students reporting exposure to bullying, low belonging, or high anxiety, rather than treating these reports as evidence of deficient resilience. The models are research analyses of aggregate patterns, not validated individual screening or diagnostic instruments.

Mathematics teaching: Self-efficacy and perceived control identify candidate instructional components for local pilot testing: mastery-oriented feedback, transparent learning goals, opportunities to correct errors, appropriately scaffolded challenges, and classroom norms that treat mistakes as part of learning. Local studies could examine whether existing referral and support routes adequately respond to students who report persistent mathematics anxiety or low confidence. Because achievement and anxiety may influence one another, future evaluations could test support models that address both learning opportunities and emotional experiences, rather than assuming that a single mindset message will resolve anxiety. Results on growth mindset and persistence provide candidate correlates for future trials, not evidence that generic mindset or perseverance programs will work across settings (Jerrim, 2026; Macnamara and Burgoyne, 2023).

Social-emotional learning and cultural adaptation: Stress resistance, cooperation, and assertiveness remain relevant constructs, but the limited evidence on their coverage does not justify a one-size-fits-all social-emotional-learning package or a program selected solely because an economy has a high IDV score. Future intervention studies could evaluate a locally adapted sequence: verify that translated measures capture the intended construct, consult students and teachers about locally appropriate expressions of cooperation and self-advocacy, conduct a small pilot, monitor differential participation and benefits, and use an experimental or strong quasi-experimental design where feasible. Economy-level IDV is an ecological descriptor and must not be used to assign a cultural label or intervention to an individual student.

### 4.5 Limitations

Several limitations define the scope of the findings. First, PISA 2022 is cross-sectional. Mean absolute SHAP identifies predictive attribution but has no sign and is not a causal effect. The fixed-effects coefficients indicate the direction and magnitude of conditional linear associations, while the multilevel coefficients provide a model-specification sensitivity check; neither removes unmeasured individual-level confounding, reverse association, or selection. Cross-economy stable predictive salience is therefore not evidence of a common psychological mechanism or a transferable intervention benefit.

Second, each focal predictor was fitted in a separate regression to avoid reducing all analyses to the intersection of differently administered questionnaire modules. This choice preserves information but means that the coefficients are not mutually adjusted: differences in coefficient magnitude can reflect different samples and omitted correlated predictors. They should not be compared as independent or direct contributions. In particular, the observational association between mathematics achievement and anxiety may operate in both directions.

Third, the analyses do not constitute a complete design-based PISA variance analysis. The initial per-economy SHAP models targeted within-sample rank structure and were unweighted, although we retained a final student-weighted sensitivity analysis. SHAP values were summarized across the entire per-economy sample rather than the test split alone, so the rankings may partly reflect sample-internal fitted structure; held-out *R*^2^ evaluates predictive performance but does not remove this limitation. The primary fixed-effects regressions used student weights normalized within economy and economy-clustered standard errors, but did not propagate all 80 BRR replicate weights; mathematics achievement did use all ten plausible values. The mixed models were deliberately unweighted because lmer prior weights are not PISA sampling weights and relied on large-sample Wald inference. Six of 21 model/plausible-value fits used the documented random-intercept fallback; final fits had no residual singularity or error, but the multilevel results remain model-based sensitivity evidence.

Fourth, missingness takes several forms. ANXMAT is missing for 22.5% of students in the full 80-economy source file, and LightGBM native routing and the MICE analysis address only student-level missingness. They cannot repair economy-level structural non-availability. The four social-emotional combinations selected for cultural follow-up failed to meet the requirement of at least 50% usable-economy coverage in every IDV tercile and consequently remain coverage-limited. No imputation can create information for a questionnaire module that an economy did not administer.

Fifth, PISA documentation and measurement practices constrain cross-economy comparability. Local wording, coding, translation, and questionnaire adaptations may not be fully visible in the international file (Jerrim et al., 2026). Planned missingness from the balanced incomplete block design is distinct from structural module non-availability. Complete-case reliability and loading diagnostics could be calculated for only four economies per scale and do not constitute formal tests of cross-economy measurement invariance or alignment. The study did not conduct such a formal measurement analysis, and residual measurement non-equivalence may influence both ranks and regression slopes.

Sixth, IDV is an economy-level index, not a measure of an individual student's cultural orientation. Its interaction with a student-level predictor is vulnerable to ecological misinterpretation and may proxy GDP, inequality, education policy, region, curriculum, post-pandemic disruption, or other economy-level conditions. Six PISA economies lacked an official IDV score and were excluded from cultural comparisons; for the subnational PISA reporting scopes QAZ and QUR, we applied the corresponding country scores for Azerbaijan and Ukraine. LTO and a sensitivity analysis excluding QAZ and QUR provide limited robustness evidence, not identification of individualism as the cause of slope variation (Johnson et al., 2024).

Seventh, the four-category rules, rank thresholds, mean aggregation, and 50% availability criterion are operational analytic choices rather than natural categories. High-IDV-aligned assignments changed under the source restriction and alternative grouping scheme and, after the availability assessment, do not support a main cultural conclusion. Four retained focal variables (FEELSAFE, RELATST, GROSAGR, MATHPERS) had bootstrap-CI upper bounds one to three ranks above 15, although the initial stable rule used a cluster mean rank of ≤ 15 and a maximum CI width of ≤ 30. With this threshold qualification, they are described as cross-tercile-stable predictive signals, not as context-invariant constructs.

Finally, PISA samples primarily 15-year-olds enrolled in school during one post-pandemic assessment cycle. Associations may differ at other ages, outside formal schooling, or in later cohorts. The practical suggestions require local measurement checks and prospective experimental or quasi-experimental evaluation before they can inform program choice.

### 4.6 Future work

Four extensions could be examined. First, formal cross-economy measurement-invariance methods suited to the PISA item design and missingness pattern would provide stronger evidence than the limited complete-case reliability and loading diagnostics used here. Second, leave-one-economy-out cross-validation (King et al., 2024; Cheung et al., 2024) would quantify out-of-economy predictive performance and identify economies for detailed study when transfer fails. Third, macro-level models incorporating GDP, educational inequality, regional system indicators, and school climate policies would test whether the IDV-associated patterns remain after economy-level confounding is addressed. Fourth, cross-cultural randomized or quasi-experimental studies would be needed to determine whether stable predictive salience corresponds to intervention outcomes across settings.

### 4.7 Conclusion

After accounting for questionnaire availability, the results could be organized into two psychologically meaningful predictor patterns across economies: a relational–institutional pattern for school belonging and an efficacy–achievement–engagement pattern for mathematics anxiety. The economy–feature availability assessment and complementary association models were secondary *post-hoc* analyses formulated after the initial rank results were known, and their findings are therefore interpreted as exploratory. In outcome-valid samples of 561,339 students in 78 economies and 475,792 students in 76 economies, respectively, the same eight focal combinations retained stable relative predictive salience. The fixed-effects models showed that greater safety and more positive student–teacher relations were associated with higher belonging, greater exposure to bullying with lower belonging, and all five focal mathematics variables with lower anxiety; the multilevel sensitivity models preserved the direction of every main association. The four social-emotional combinations selected for cultural follow-up failed the cross-tercile availability criterion; we report their qualified results for transparency but exclude them from the primary conclusions. Additional all-feature rule classifications lacked focal association models and are reported in the supplement as exploratory signals rather than as primary conclusions. By bringing predictor availability, ranking stability, and association direction into one analysis, the study identifies candidate domains for local assessment and prospective experimental or quasi-experimental evaluation, but does not establish common mechanisms, causal effects, or directly transferable policies.

## Data Availability

The original contributions presented in the study are included in the article/Supplementary material, further inquiries can be directed to the corresponding author.
