# Supplementary material for: Cross-economy stability of predictor rankings for adolescent school belonging and mathematics anxiety: an explainable machine-learning analysis of PISA 2022
Source: Front Psychol. 2026 Sep 3;17:1875261. doi: 10.3389/fpsyg.2026.1875261 (PMC13582362; doi:10.3389/fpsyg.2026.1875261)
Supplement: Supplementary file 1 [file Data_Sheet_1.pdf]

Supplementary Material

1 OVERVIEW

This supplementary document accompanies the main manuscript "Cross-Economy Stability of Predictor Rankings for Adolescent School Belonging and Mathematics Anxiety: An Explainable Machine-Learning Analysis of PISA 2022" by Yuan Liao, Qin Peng, and Runlin Li. Tables S1–S16 report the initial unadjusted analyses and their sensitivity checks; these tables preserve the initial operational rank codes for reproducibility but do not determine the primary cultural interpretation. The initial unadjusted classification is retained as a record of model-based rank patterns. Tables S17–S18 provide the secondary post hoc coverage-adjusted and association evidence used in the primary interpretation. Supporting analyses include the economy–feature availability assessment, the mean-versus-median aggregation comparison, and a PISA documentation check.

Data sources.

PISA 2022 micro-data are publicly available from the OECD at `https://webfs.oecd.org/pisa2022/`. The documented 2023 cultural-dimension scores used for the IDV and LTO groupings are available from the official country-comparison page at `https://www.theculturefactor.com/country-comparison-tool`. The IDV mapping contains 74 economies, divided into 25 low-, 25 mid-, and 24 high-IDV economies. BRN, KHM, KSV, MAC, PSE, and UZB have no official IDV score and are omitted from IDV-based analyses rather than assigned substitute values. QAZ and QUR are matched to Azerbaijan and Ukraine country scores, respectively; TAP uses the direct Taiwan score.

Index.

- **Table S1** – Initial unadjusted classification of operational rank patterns (all 73 features, both outcomes, IDV scheme).
- **Table S2** – Per-economy LightGBM fit summary (sample size, R^2, top-5 features), both outcomes.
- **Table S3** – Sensitivity classification under long-term-orientation (LTO) terciles, all features, both outcomes.
- **Table S4** – Per-economy Cronbach's α for both BELONG and ANXMAT scales.
- **Table S5** – Bootstrap 95% confidence intervals and pairwise cross-cluster rank agreement (both outcomes).
- **Table S6** – Stricter alternative rules vs. initial unadjusted operational codes (both outcomes).
- **Table S7** – Treatment of QAZ, QUR, TAP, and MAC in the subnational country-score sensitivity.
- **Table S8** – Exploratory low-IDV-aligned rank differences under a direction-only rule.
- **Table S9** – Multiple-imputation (MICE) per-economy fit summary for ANXMAT.
- **Table S10** – Predictor dictionary for all 73 candidate variables.
- **Table S11** – Official 2023 IDV scores and tercile membership.
- **Table S12** – Weighted versus unweighted SHAP sensitivity.
- **Table S13** – Random-seed stability.
- **Table S14** – Plausible-values sensitivity.
- **Table S15** – Percentile-threshold sensitivity.
- **Table S16** – Mean versus median aggregation comparison.
- **Table S17** – Economy–feature availability and focal percentile summary.
- **Table S18** – Complete focal fixed-effects and multilevel association estimates and final model status.

2 TABLE S1. INITIAL UNADJUSTED CLASSIFICATION OF OPERATIONAL RANK PATTERNS

2.1 S1a. Outcome: BELONG (school belonging)

Table S1: Initial unadjusted classification of operational rank patterns for BELONG (school belonging): all features sorted by the initial operational code (`U` = cross-tercile stable, `W` = high-IDV-aligned, `C` = low-IDV-aligned, and `I` = not classified) and then by mean rank in the high-individualism cluster. `Rank` columns show within-economy mean rank averaged across economies in each official individualism (IDV) tercile (1 = most important). `CI width` = maximum across clusters of the bootstrap 95% CI width on cluster mean rank. `Perm. p` = permutation p-value for the difference in mean rank between low-IDV and high-IDV clusters; `BH-sig.` indicates Benjamini–Hochberg false-discovery-rate-corrected significance at $q = 0.05$. These initial codes are retained for reproducibility and do not determine the coverage-adjusted interpretation.

Code	Feature label	Operational code	Rank low	Rank mid	Rank high	CI width	Perm. p	BH-sig.
BULLIED	Exposure to bullying	U	3.1	3.1	2.3	1.3	0.044	no
RELATST	Teacher–student relations	U	9.6	3.6	6.0	13.1	0.304	no
FEELSAFE	School safety	U	3.0	1.5	10.0	16.6	0.049	no
STRESAGR	Stress resistance	W	44.7	18.8	12.0	17.1	< 0.001	yes
ASSERAGR	Assertiveness	W	42.1	13.8	12.2	16.0	< 0.001	yes
OPENART	Openness to art	W	26.0	17.5	14.9	11.0	0.004	yes
CURIOAGR	Curiosity	C	12.2	20.6	29.8	10.7	< 0.001	yes
COOPAGR	Cooperation tendency	I	39.5	22.4	16.0	21.2	0.003	yes
FAMSUP	Family support	I	17.6	16.9	17.8	11.0	0.967	no
PERSEVAGR	Perseverance	I	36.2	18.9	19.5	15.8	0.003	yes
DISCLIM	Disciplinary climate	I	15.5	20.2	20.5	9.0	0.094	no
ST004D01T	Gender	I	31.9	42.2	22.0	15.8	0.111	no

continued on next page

Code	Feature label	Operational code	Rank low	Rank mid	Rank high	CI width	Perm. p	BH-sig.
PV1SCIE	Science achievement (PV1)	I	17.1	28.2	22.2	14.3	0.173	no
CREATEFF	Creative self-efficacy	I	14.1	14.7	22.4	11.3	0.018	yes
EMOCOAGR	Emotional control	I	19.2	13.6	23.1	15.0	0.435	no
ICTREG	ICT regulation	I	34.5	32.3	23.2	12.5	0.005	yes
ICTQUAL	ICT quality	I	34.5	26.8	23.7	11.8	0.008	yes
PROBSELF	Self-regulation problems	I	21.3	27.2	24.5	11.5	0.422	no
MATHPERS	Math persistence	I	23.4	30.8	24.9	10.8	0.691	no
PV1READ	Reading achievement (PV1)	I	17.1	26.5	26.1	10.4	0.018	yes
MATHEFF	Math self-efficacy	I	27.8	28.7	28.6	10.0	0.831	no
EMPATAGR	Empathy	I	43.3	23.3	28.8	13.5	0.005	yes
COGACMCO	Cognitive activation	I	27.9	28.7	29.0	8.9	0.719	no
HOMEPOS	Home possessions	I	22.3	26.1	29.1	11.3	0.071	no
FAMSUPSL	Family support self-dir.	I	25.4	29.0	30.0	11.3	0.192	no
ESCS	Socio-economic index	I	28.4	34.8	30.2	12.8	0.633	no
MATHEF21	Math self-efficacy 21st-c.	I	27.8	32.1	32.0	8.9	0.182	no
SDLEFF	SDL self-efficacy	I	26.4	32.5	32.3	12.9	0.123	no
BMMJ1	Mother occupation	I	32.6	34.3	32.5	11.4	0.972	no
GROSAGR	Growth mindset	I	24.0	29.2	33.1	10.6	0.007	yes
ICTEFFIC	ICT self-efficacy	I	32.5	32.5	33.7	11.4	0.765	no
EXPOFA	Exposure formal math	I	29.4	35.9	33.7	9.2	0.189	no
BFMJ2	Father occupation	I	28.2	31.5	34.2	8.7	0.043	no
COGACRCO	Cognitive reasoning	I	28.8	30.5	34.3	8.8	0.049	no
FISCED	Father education	I	25.0	38.8	34.9	12.0	0.022	no
PV1MATH	Math achievement (PV1)	I	19.0	33.4	35.3	12.3	<0.001	yes
ICTFEED	ICT feedback	I	41.4	39.2	35.8	10.8	0.100	no
EXPO21ST	Exposure 21c math	I	30.6	34.6	36.1	8.2	0.042	no
ICTWKEND	ICT weekend	I	39.9	34.8	36.2	9.8	0.287	no
TEACHSUP	Math teacher support	I	26.3	39.8	37.0	12.1	0.017	yes
FAMCON	Math familiarity	I	42.3	35.8	37.1	11.7	0.143	no

continued on next page

Code	Feature label	Operational code	Rank low	Rank mid	Rank high	CI width	Perm. p	BH-sig.
INFOSEEK	Information seeking	I	30.1	31.5	37.5	11.3	0.029	no
HISEI	Parent ISEI	I	29.8	40.2	37.5	9.0	0.014	yes
ICTINFO	ICT info-seeking	I	37.6	33.5	37.8	10.3	0.963	no
ICTWKDY	ICT weekday	I	41.5	40.2	37.8	9.4	0.203	no
MISCED	Mother education	I	27.8	39.2	38.3	11.1	0.010	yes
IMAGINE	Imagination	I	34.4	31.8	38.4	10.9	0.287	no
ICTENQ	ICT inquiry use	I	41.6	39.4	38.5	9.3	0.294	no
ICTOUT	ICT out-of-school use	I	38.4	39.2	38.7	9.1	0.936	no
LANGN	Language at home	I	40.1	45.2	39.0	15.8	0.831	no
ICTSUBJ	ICT subject lessons	I	37.0	37.3	39.5	10.2	0.431	no
IMMIG	Immigration status	I	46.6	54.4	40.7	13.8	0.145	no
ICTRES	ICT resources	I	24.0	34.8	41.1	17.7	0.008	yes
FEELLAH	Feelings learning home	I	33.2	40.5	41.8	10.6	0.023	no
SCHRISK	School risk	I	32.3	41.5	43.7	12.2	0.016	yes
LIFESAT	Life satisfaction	I	39.5	50.5	44.7	18.9	0.358	no
BODYIMA	Body image	I	40.2	51.2	45.9	17.1	0.278	no
EXPWB	Experienced wellbeing	I	39.7	52.4	46.0	15.6	0.179	no
AGE	Age	I	41.2	43.0	46.3	11.5	0.109	no
PSYCHSYM	Psychosomatic symptoms	I	39.5	51.4	46.4	16.5	0.176	no
SOCCON	Social connection	I	39.3	52.2	46.9	15.3	0.125	no
MISSSC	Missing school	I	37.4	49.5	50.6	10.8	0.003	yes
SOCONPA	Social conn. parents	I	41.4	56.8	50.6	11.0	0.014	yes
ICTSCH	ICT availability school	I	43.4	53.1	51.0	6.9	0.005	yes
GRADE	Grade vs modal	I	40.6	55.2	52.1	10.4	0.003	yes
CREATOPN	Creative openness	I	43.7	46.9	53.0	12.8	0.016	yes
ICTAVSCH	ICT use at school	I	47.0	55.8	53.2	6.1	0.009	yes
SKIPPING	Class skipping	I	44.2	55.4	53.3	7.6	< 0.001	yes
ICTHOME	ICT availability home	I	46.3	55.2	53.4	6.2	0.003	yes
PARINVOL	Parental involvement	I	42.2	52.0	54.3	8.9	< 0.001	yes
PASCHPOL	School parent policy	I	43.8	48.6	54.7	11.0	0.002	yes
ICTAVHOM	ICT use at home	I	46.5	56.1	55.1	6.0	< 0.001	yes

continued on next page

Code	Feature label	Operational code	Rank low	Rank mid	Rank high	CI width	Perm. p	BH-sig.
CURSUPP	Current support	I	41.9	51.6	56.6	11.5	< 0.001	yes

2.2 S1b. Outcome: ANXMAT (mathematics anxiety)

Table S1: Initial unadjusted classification of operational rank patterns for ANXMAT (mathematics anxiety): all features sorted by the initial operational code (`U` = cross-tercile stable, `W` = high-IDV-aligned, `C` = low-IDV-aligned, and `I` = not classified) and then by mean rank in the high-individualism cluster. `Rank` columns show within-economy mean rank averaged across economies in each official individualism (IDV) tercile (1 = most important). `CI width` = maximum across clusters of the bootstrap 95% CI width on cluster mean rank. `Perm. p` = permutation p-value for the difference in mean rank between low-IDV and high-IDV clusters; `BH-sig.` indicates Benjamini–Hochberg false-discovery-rate-corrected significance at $q = 0.05$. These initial codes are retained for reproducibility and do not determine the coverage-adjusted interpretation.

Code	Feature label	Operational code	Rank low	Rank mid	Rank high	CI width	Perm. p	BH-sig.
MATHEFF	Math self-efficacy	U	5.8	2.0	1.5	10.0	0.018	yes
MATHEF21	Math self-efficacy 21st-c.	U	6.7	2.6	2.9	9.1	0.035	no
PV1MATH	Math achievement (PV1)	U	6.7	6.4	4.2	4.2	0.041	no
MATHPERS	Math persistence	U	4.6	5.3	12.5	7.2	< 0.001	yes
GROSAGR	Growth mindset	U	11.6	8.5	12.6	9.8	0.748	no
STRESAGR	Stress resistance	W	42.6	15.2	9.6	17.8	< 0.001	yes
TEACHSUP	Math teacher support	W	27.8	24.8	14.1	13.1	0.002	yes
PROBSELF	Self-regulation problems	I	16.7	14.3	13.3	9.7	0.329	no
FAMCON	Math familiarity	I	31.7	22.0	17.7	15.7	0.012	yes
EXPO21ST	Exposure 21c math	I	12.7	17.4	19.2	7.7	0.020	yes
ST004D01T	Gender	I	16.0	31.6	19.9	15.1	0.515	no
EMOCOAGR	Emotional control	I	16.3	10.3	20.4	16.5	0.437	no
CREATEFF	Creative self-efficacy	I	19.5	20.6	22.2	10.7	0.440	no
DISCLIM	Disciplinary climate	I	13.7	21.8	22.2	10.6	0.019	yes

continued on next page

Code	Feature label	Operational code	Rank low	Rank mid	Rank high	CI width	Perm. p	BH-sig.
RELATST	Teacher–student relations	I	24.8	27.0	22.2	11.2	0.455	no
PERSEVAGR	Perseverance	I	37.6	26.8	22.6	13.6	0.002	yes
EXPOFA	Exposure formal math	I	16.2	21.4	23.1	8.0	0.010	yes
OPENART	Openness to art	I	24.8	23.1	23.5	10.1	0.697	no
ICTOUT	ICT out-of-school use	I	34.9	24.8	23.8	12.5	0.005	yes
BULLIED	Exposure to bullying	I	16.0	27.6	24.4	13.6	0.065	no
EMPATAGR	Empathy	I	43.6	19.8	25.1	13.3	0.002	yes
COGACMCO	Cognitive activation	I	22.8	21.0	25.3	9.6	0.434	no
PV1SCIE	Science achievement (PV1)	I	20.4	31.5	26.0	11.3	0.154	no
INFOSEEK	Information seeking	I	26.0	30.1	29.4	12.1	0.342	no
IMAGINE	Imagination	I	22.2	14.8	29.9	15.3	0.111	no
PV1READ	Reading achievement (PV1)	I	19.7	27.9	30.6	11.1	0.003	yes
ICTINFO	ICT info-seeking	I	37.5	34.0	31.4	10.9	0.117	no
ASSERAGR	Assertiveness	I	43.6	31.1	31.5	12.8	0.005	yes
CURIOAGR	Curiosity	I	23.5	27.8	31.5	8.2	0.010	yes
COGACRCO	Cognitive reasoning	I	24.0	27.6	31.6	9.4	0.018	yes
FAMSUP	Family support	I	23.9	28.6	32.0	9.1	0.017	yes
FEELSAFE	School safety	I	30.0	30.1	32.0	12.8	0.643	no
ICTWKDY	ICT weekday	I	38.7	36.8	34.8	9.8	0.233	no
SDLEFF	SDL self-efficacy	I	24.3	36.0	35.1	9.0	< 0.001	yes
BFMJ2	Father occupation	I	35.7	40.2	35.3	10.0	0.877	no
ICTWKEND	ICT weekend	I	39.8	34.3	35.9	9.9	0.240	no
ICTSUBJ	ICT subject lessons	I	35.2	37.6	36.0	11.3	0.824	no
FAMSUPSL	Family support self-dir.	I	31.0	35.3	36.0	9.8	0.100	no
ICTQUAL	ICT quality	I	36.0	33.5	36.8	8.7	0.811	no
ICTREG	ICT regulation	I	35.0	32.6	36.8	9.3	0.568	no
ICTFEED	ICT feedback	I	38.5	37.1	38.1	8.5	0.878	no
COOPAGR	Cooperation tendency	I	41.5	39.0	38.6	12.7	0.502	no
ICTENQ	ICT inquiry use	I	38.2	32.5	38.9	8.8	0.820	no

continued on next page

Code	Feature label	Operational code	Rank low	Rank mid	Rank high	CI width	Perm. p	BH-sig.
ICTEFFIC	ICT self-efficacy	I	38.5	38.3	39.0	8.8	0.876	no
FISCED	Father education	I	32.3	44.1	39.2	10.8	0.078	no
MISCED	Mother education	I	33.1	41.8	39.5	10.9	0.120	no
HOMEPOS	Home possessions	I	32.3	38.7	39.7	9.4	0.023	no
BMMJ1	Mother occupation	I	36.5	38.0	40.9	8.3	0.122	no
ESCS	Socio-economic index	I	36.0	43.0	41.0	9.0	0.109	no
HISEI	Parent ISEI	I	36.0	41.2	42.6	9.0	0.018	yes
FEELLAH	Feelings learning home	I	30.3	40.3	42.9	9.4	0.002	yes
LANGN	Language at home	I	42.2	53.8	44.7	13.1	0.595	no
PSYCHSYM	Psychosomatic symptoms	I	36.5	50.2	44.9	18.1	0.144	no
BODYIMA	Body image	I	39.7	51.7	46.8	14.9	0.149	no
AGE	Age	I	39.8	49.8	47.2	8.6	0.011	yes
SCHRISK	School risk	I	39.2	46.5	47.6	9.5	0.006	yes
GRADE	Grade vs modal	I	45.8	54.7	48.0	11.5	0.546	no
IMMIG	Immigration status	I	47.5	55.2	48.2	9.2	0.807	no
LIFESAT	Life satisfaction	I	42.5	52.3	48.7	12.8	0.135	no
ICTRES	ICT resources	I	36.5	44.0	49.6	12.1	0.004	yes
EXPWB	Experienced wellbeing	I	43.5	54.8	49.8	11.3	0.073	no
ICTSCH	ICT availability school	I	44.8	51.9	50.9	6.4	0.011	yes
SOCCON	Social connection	I	42.0	52.8	51.0	10.9	0.017	yes
SKIPPING	Class skipping	I	44.2	56.2	51.3	8.7	0.025	no
SOCONPA	Social conn. parents	I	45.3	53.9	52.0	8.1	0.030	no
ICTHOME	ICT availability home	I	44.3	53.8	52.7	6.5	0.005	yes
PARINVOL	Parental involvement	I	44.2	49.9	53.5	8.6	0.002	yes
PASCHPOL	School parent policy	I	43.5	51.2	53.8	8.5	0.002	yes
CREATOPN	Creative openness	I	43.3	49.6	54.0	9.2	< 0.001	yes
ICTAVHOM	ICT use at home	I	45.1	56.1	54.2	8.0	< 0.001	yes
ICTAVSCH	ICT use at school	I	46.8	55.7	54.3	7.1	0.005	yes
MISSSC	Missing school	I	44.8	57.4	54.7	8.9	< 0.001	yes
CURSUPP	Current support	I	41.9	52.0	56.2	9.0	< 0.001	yes

3 TABLE S2. PER-ECONOMY FIT SUMMARY

3.1 S2a. Outcome: BELONG

Table S2: Per-economy LightGBM fit summary for BELONG in the initial unadjusted analysis: sample size after outcome-listwise deletion, held-out 20% test R^2, best boosting iteration selected on the validation split, and top-5 features ranked by mean |SHAP|.

Economy	n	R^2	Best iter	Top 1	Top 2	Top 3	Top 4	Top 5
ALB	4,743	0.262	59	RELATST	FEELSAFE	CURIOAGR	BULLIED	DISCLIM
ARE	22,265	0.293	82	FEELSAFE	RELATST	LIFESAT	BULLIED	EMPATAGR
ARG	10,888	0.239	117	BULLIED	FEELSAFE	RELATST	ASSERAGR	ST004D01T
AUS	12,896	0.362	103	FEELSAFE	BULLIED	STRESAGR	COOPAGR	ASSERAGR
AUT	5,971	0.244	69	FEELSAFE	COOPAGR	RELATST	BULLIED	STRESAGR
BEL	7,747	0.205	133	FEELSAFE	BULLIED	RELATST	STRESAGR	ASSERAGR
BGR	5,352	0.145	65	FEELSAFE	BULLIED	PERSEVAGR	RELATST	HOMEPOS
BRA	9,567	0.222	165	FEELSAFE	BULLIED	RELATST	ASSERAGR	LIFESAT
BRN	5,485	0.229	97	FEELSAFE	BULLIED	ASSERAGR	STRESAGR	EMOCOAGR
CAN	20,131	0.362	86	FEELSAFE	BULLIED	COOPAGR	STRESAGR	ASSERAGR
CHE	6,602	0.278	144	FEELSAFE	COOPAGR	BULLIED	STRESAGR	RELATST
CHL	5,672	0.224	103	FEELSAFE	BULLIED	RELATST	COOPAGR	STRESAGR
COL	7,184	0.248	105	RELATST	FEELSAFE	COOPAGR	BULLIED	PV1MATH
CRI	5,215	0.225	60	FEELSAFE	BULLIED	ASSERAGR	COOPAGR	RELATST
CZE	8,181	0.191	100	FEELSAFE	BULLIED	COOPAGR	RELATST	ASSERAGR
DEU	5,278	0.220	68	BULLIED	COOPAGR	RELATST	STRESAGR	ASSERAGR
DNK	5,712	0.283	78	BULLIED	RELATST	COOPAGR	STRESAGR	ST004D01T
DOM	6,042	0.225	70	FEELSAFE	RELATST	BULLIED	CURIOAGR	PV1SCIE
ESP	29,547	0.196	109	LIFESAT	BULLIED	COOPAGR	ASSERAGR	OPENART
EST	6,283	0.352	125	FEELSAFE	COOPAGR	BULLIED	RELATST	STRESAGR
FIN	9,736	0.343	80	FEELSAFE	BULLIED	COOPAGR	ST004D01T	RELATST
FRA	6,431	0.304	104	FEELSAFE	BULLIED	RELATST	COOPAGR	EXPWB
GBR	11,188	0.343	94	FEELSAFE	BULLIED	COOPAGR	STRESAGR	RELATST
GEO	5,609	0.179	63	FEELSAFE	RELATST	BULLIED	EMPATAGR	ST004D01T
GRC	6,215	0.255	82	FEELSAFE	COOPAGR	RELATST	BULLIED	STRESAGR
GTM	4,844	0.226	62	FEELSAFE	RELATST	CURIOAGR	BULLIED	EMOCOAGR
HKG	5,614	0.311	99	LIFESAT	COOPAGR	RELATST	ASSERAGR	FEELSAFE
HRV	5,982	0.232	82	FEELSAFE	COOPAGR	BULLIED	RELATST	STRESAGR
HUN	5,988	0.365	96	FEELSAFE	BULLIED	COOPAGR	LIFESAT	ASSERAGR
IDN	13,069	0.191	73	FEELSAFE	EMOCOAGR	CURIOAGR	BULLIED	FAMSUP
IRL	5,501	0.378	91	LIFESAT	FEELSAFE	COOPAGR	BULLIED	STRESAGR
ISL	3,114	0.228	74	FEELSAFE	BULLIED	RELATST	COOPAGR	STRESAGR
ITA	10,397	0.309	78	FEELSAFE	BULLIED	COOPAGR	ASSERAGR	RELATST
JAM	3,373	0.222	44	FEELSAFE	BULLIED	RELATST	ASSERAGR	EMOCOAGR
JOR	6,963	0.246	55	RELATST	BULLIED	FEELSAFE	CURIOAGR	CREATEFF
JPN	5,703	0.215	81	RELATST	BULLIED	TEACHSUP	ICTQUAL	MATHEFF
KAZ	19,500	0.239	96	FEELSAFE	RELATST	CREATEFF	CURIOAGR	BULLIED
KHM	4,841	0.129	32	PV1READ	FEELSAFE	PV1MATH	BULLIED	MATHPERS
KOR	6,379	0.289	96	COOPAGR	RELATST	FEELSAFE	ASSERAGR	EMOCOAGR
LTU	7,053	0.250	51	FEELSAFE	COOPAGR	BULLIED	ASSERAGR	RELATST
LVA	5,192	0.236	49	FEELSAFE	RELATST	BULLIED	STRESAGR	ASSERAGR
MAC	4,380	0.342	43	LIFESAT	FEELSAFE	COOPAGR	BULLIED	PSYCHSYM
MAR	6,476	0.127	70	FEELSAFE	BULLIED	DISCLIM	FAMSUP	PV1READ
MDA	5,964	0.231	108	RELATST	BULLIED	ST004D01T	PERSEVAGR	CREATEFF
MEX	6,145	0.180	50	FEELSAFE	LIFESAT	COOPAGR	BULLIED	BODYIMA

Economy	n	R^2	Best iter	Top 1	Top 2	Top 3	Top 4	Top 5
MKD	5,945	0.236	59	FEELSAFE	RELATST	BULLIED	CURIOAGR	HOMEPOS
MLT	2,920	0.313	60	FEELSAFE	COOPAGR	BULLIED	ASSERAGR	RELATST
MNE	5,425	0.197	117	FEELSAFE	BULLIED	RELATST	CREATEFF	PERSEVAGR
MNG	6,769	0.232	70	BULLIED	RELATST	FEELSAFE	CURIOAGR	CREATEFF
MYS	6,941	0.216	75	FEELSAFE	BULLIED	CURIOAGR	EMOCOAGR	LANGN
NLD	4,690	0.279	83	FEELSAFE	COOPAGR	LIFESAT	BULLIED	ASSERAGR
NOR	6,118	0.279	37	FEELSAFE	BULLIED	COOPAGR	FAMSUP	RELATST
NZL	4,427	0.352	51	LIFESAT	FEELSAFE	BULLIED	PSYCHSYM	COOPAGR
PAN	3,671	0.170	60	FEELSAFE	LIFESAT	RELATST	PV1MATH	BULLIED
PER	6,747	0.199	111	RELATST	FEELSAFE	BULLIED	COOPAGR	PERSEVAGR
PHL	6,973	0.234	55	FEELSAFE	PV1READ	CURIOAGR	FAMSUP	BULLIED
POL	5,849	0.180	67	COOPAGR	FEELSAFE	BULLIED	STRESAGR	RELATST
PRT	6,634	0.296	73	FEELSAFE	BULLIED	COOPAGR	STRESAGR	RELATST
PRY	4,560	0.254	83	FEELSAFE	RELATST	BULLIED	PV1READ	PV1SCIE
PSE	7,171	0.224	142	RELATST	BULLIED	FEELSAFE	CREATEFF	CURIOAGR
QAT	6,681	0.229	96	FEELSAFE	BULLIED	RELATST	CURIOAGR	ST004D01T
QAZ	6,021	0.150	55	BULLIED	RELATST	FEELSAFE	CURIOAGR	ICTRES
QUR	3,516	0.251	68	RELATST	BULLIED	FEELSAFE	ASSERAGR	CURIOAGR
ROU	7,068	0.298	104	FEELSAFE	COOPAGR	BULLIED	RELATST	ASSERAGR
SAU	6,505	0.309	90	FEELSAFE	RELATST	BULLIED	LIFESAT	BODYIMA
SGP	6,557	0.303	139	COOPAGR	FEELSAFE	BULLIED	RELATST	ASSERAGR
SLV	5,810	0.174	41	FEELSAFE	COOPAGR	RELATST	BULLIED	PV1MATH
SRB	6,170	0.196	80	FEELSAFE	BULLIED	RELATST	ASSERAGR	CREATEFF
SVK	5,469	0.209	80	FEELSAFE	BULLIED	RELATST	STRESAGR	ASSERAGR
SVN	6,364	0.256	60	FEELSAFE	BULLIED	LIFESAT	RELATST	PERSEVAGR
SWE	5,820	0.240	89	FEELSAFE	RELATST	BULLIED	ICTRES	ST004D01T
TAP	5,786	0.350	95	FEELSAFE	COOPAGR	RELATST	STRESAGR	BULLIED
THA	8,346	0.233	83	RELATST	FEELSAFE	BULLIED	EMOCOAGR	CREATEFF
TUR	7,210	0.203	56	FEELSAFE	BULLIED	COOPAGR	RELATST	ASSERAGR
URY	5,981	0.242	70	FEELSAFE	COOPAGR	BULLIED	RELATST	STRESAGR
USA	4,073	0.280	57	FEELSAFE	BULLIED	RELATST	ICTEFFIC	FAMSUP
UZB	6,695	0.167	80	FEELSAFE	CURIOAGR	BULLIED	DISCLIM	PV1SCIE
VNM	6,009	0.232	57	FEELSAFE	RELATST	EMOCOAGR	BULLIED	SDLEFF

3.2 S2b. Outcome: ANXMAT

Table S2: Per-economy LightGBM fit summary for ANXMAT in the initial unadjusted analysis: sample size after outcome-listwise deletion, held-out 20% test R^2, best boosting iteration selected on the validation split, and top-5 features ranked by mean |SHAP|.

Economy	n	R^2	Best iter	Top 1	Top 2	Top 3	Top 4	Top 5
ALB	3,674	0.216	93	MATHEFF	MATHEF21	DISCLIM	MATHPERS	FAMCON
ARE	20,441	0.280	85	MATHEF21	MATHPERS	MATHEFF	PV1MATH	PSYCHSYM
ARG	7,857	0.145	63	MATHEFF	PV1MATH	MATHEF21	EMOCOAGR	MATHPERS
AUS	12,327	0.237	111	STRESAGR	MATHEFF	MATHEF21	MATHPERS	PV1MATH
AUT	5,228	0.308	78	MATHEFF	MATHEF21	STRESAGR	PV1MATH	TEACHSUP
BEL	6,667	0.208	81	STRESAGR	MATHEF21	MATHEFF	CREATEFF	GROSAGR
BGR	4,476	0.142	49	MATHPERS	MATHEF21	MATHEFF	FAMCON	EMOCOAGR
BRA	7,566	0.243	103	MATHEFF	MATHEF21	STRESAGR	MATHPERS	ST004D01T
BRN	4,822	0.138	75	STRESAGR	MATHEF21	MATHEFF	EMOCOAGR	PROBSELF
CAN	18,834	0.280	104	MATHEFF	STRESAGR	MATHEF21	PV1MATH	MATHPERS
CHE	5,175	0.263	102	STRESAGR	MATHEFF	MATHEF21	PV1MATH	OPENART
CHL	5,372	0.271	67	MATHEFF	STRESAGR	MATHPERS	ST004D01T	MATHEF21
COL	6,342	0.126	50	MATHEF21	ST004D01T	MATHEFF	MATHPERS	IMAGINE
CRI	5,646	0.181	48	STRESAGR	MATHEF21	MATHEFF	PV1MATH	ST004D01T
CZE	7,567	0.304	104	MATHEFF	MATHEF21	STRESAGR	PV1MATH	TEACHSUP
DEU	4,812	0.329	201	MATHEFF	STRESAGR	MATHEF21	PV1MATH	CREATEFF
DNK	4,967	0.359	54	MATHEFF	PV1MATH	MATHEF21	STRESAGR	ST004D01T
DOM	3,343	0.154	38	MATHEF21	MATHPERS	EXPO21ST	ST004D01T	MATHEFF
ESP	27,646	0.288	160	MATHEF21	MATHEFF	STRESAGR	PV1MATH	PSYCHSYM
EST	6,018	0.301	105	MATHEFF	STRESAGR	MATHEF21	PV1MATH	EMOCOAGR
FIN	8,450	0.293	62	MATHEFF	STRESAGR	PV1MATH	MATHEF21	PROBSELF
FRA	5,478	0.260	72	STRESAGR	PV1MATH	MATHEFF	MATHEF21	IMAGINE
GBR	10,072	0.295	138	STRESAGR	MATHEFF	MATHEF21	PV1MATH	ST004D01T
GEO	4,497	0.158	88	MATHPERS	MATHEFF	MATHEF21	EMOCOAGR	BULLIED
GRC	5,770	0.317	59	MATHEFF	MATHEF21	STRESAGR	MATHPERS	PV1MATH
GTM	3,736	0.226	75	EMOCOAGR	MATHEFF	PV1MATH	BULLIED	EXPOFA
HKG	5,521	0.288	90	MATHEFF	MATHEF21	PV1MATH	STRESAGR	MATHPERS
HRV	5,579	0.262	62	MATHEF21	STRESAGR	TEACHSUP	MATHEFF	GROSAGR
HUN	5,465	0.302	80	MATHEFF	PV1MATH	STRESAGR	TEACHSUP	FAMCON
IRL	5,278	0.359	53	MATHEFF	MATHEF21	STRESAGR	PSYCHSYM	ST004D01T
ISL	2,765	0.245	70	MATHEFF	STRESAGR	MATHEF21	PV1MATH	DISCLIM
ITA	9,749	0.202	96	STRESAGR	PV1MATH	MATHEF21	MATHEFF	MATHPERS
JAM	2,322	0.157	87	MATHEFF	MATHEF21	GROSAGR	MATHPERS	EXPO21ST
JOR	5,594	0.206	55	MATHPERS	MATHEF21	MATHEFF	DISCLIM	COGACMCO
JPN	5,680	0.252	161	MATHEF21	MATHEFF	MATHPERS	PV1READ	PROBSELF
KAZ	18,876	0.169	85	MATHEF21	PROBSELF	EMOCOAGR	MATHPERS	PV1SCIE
KHM	4,184	0.146	102	MATHEFF	DISCLIM	MATHPERS	PV1MATH	PROBSELF
KOR	6,317	0.262	113	MATHEFF	STRESAGR	MATHEF21	MATHPERS	ICTOUT
KSV	4,428	0.146	84	MATHEFF	MATHPERS	MATHEF21	GROSAGR	PV1MATH
LTU	6,563	0.275	87	STRESAGR	PV1MATH	MATHEFF	MATHEF21	GROSAGR
LVA	4,623	0.305	45	MATHEFF	PV1MATH	STRESAGR	MATHEF21	EMOCOAGR
MAC	4,314	0.274	58	MATHEF21	MATHEFF	STRESAGR	PROBSELF	GROSAGR
MAR	4,296	0.164	58	MATHPERS	MATHEF21	MATHEFF	ST004D01T	PV1MATH
MDA	5,518	0.243	103	MATHEFF	PV1MATH	MATHEF21	MATHPERS	GROSAGR
MEX	5,545	0.134	52	MATHEFF	PSYCHSYM	MATHEF21	MATHPERS	EMOCOAGR
MKD	4,868	0.175	87	MATHEFF	MATHPERS	MATHEF21	FAMCON	GROSAGR
MLT	2,679	0.350	51	MATHEFF	STRESAGR	MATHEF21	PV1MATH	IMAGINE

Economy	n	R^2	Best iter	Top 1	Top 2	Top 3	Top 4	Top 5
MNE	4,768	0.228	57	MATHEFF	MATHEF21	MATHPERS	FAMCON	PV1MATH
MNG	6,262	0.264	83	MATHEFF	MATHPERS	MATHEF21	ST004D01T	BULLIED
MYS	6,351	0.199	90	EMOCOAGR	MATHEF21	MATHEFF	GROSAGR	MATHPERS
NLD	4,352	0.173	37	MATHEFF	MATHEF21	STRESAGR	PROBSELF	PSYCHSYM
NOR	5,466	0.347	88	MATHEFF	ST004D01T	MATHEF21	PV1MATH	BULLIED
NZL	4,016	0.246	83	STRESAGR	MATHEFF	MATHEF21	PSYCHSYM	EMOCOAGR
PAN	2,098	0.152	42	MATHEFF	MATHPERS	PV1READ	EMOCOAGR	PSYCHSYM
PER	5,150	0.202	68	MATHEFF	MATHEF21	MATHPERS	EMOCOAGR	ST004D01T
POL	5,406	0.215	96	MATHEFF	MATHEF21	PV1MATH	STRESAGR	MATHPERS
PRT	5,913	0.267	90	STRESAGR	MATHEFF	MATHEF21	MATHPERS	PV1MATH
PRY	3,810	0.233	62	MATHEFF	EMOCOAGR	PV1MATH	MATHPERS	DISCLIM
PSE	6,162	0.196	51	MATHEFF	MATHEF21	MATHPERS	PV1MATH	GROSAGR
QAT	5,343	0.188	165	MATHEFF	MATHPERS	PV1MATH	MATHEF21	PV1READ
QAZ	3,059	0.115	55	MATHEFF	MATHPERS	MATHEF21	PV1MATH	COGACMCO
QUR	2,962	0.194	78	MATHEFF	STRESAGR	MATHEF21	MATHPERS	BULLIED
ROU	6,356	0.139	130	STRESAGR	PV1MATH	MATHPERS	MATHEFF	GROSAGR
SAU	5,816	0.233	93	MATHEFF	MATHPERS	MATHEF21	GROSAGR	DISCLIM
SGP	6,524	0.282	63	STRESAGR	MATHEF21	MATHEFF	PV1MATH	MATHPERS
SLV	4,678	0.129	69	MATHPERS	MATHEF21	PV1MATH	EMOCOAGR	ST004D01T
SRB	5,514	0.178	72	MATHEFF	MATHEF21	STRESAGR	MATHPERS	FAMCON
SVK	5,038	0.257	115	MATHEF21	MATHEFF	STRESAGR	PV1MATH	MATHPERS
SVN	6,166	0.203	60	MATHEFF	MATHEF21	STRESAGR	PSYCHSYM	GROSAGR
SWE	5,260	0.234	68	MATHEFF	PV1MATH	ST004D01T	MATHEF21	BULLIED
TAP	5,747	0.258	76	MATHEF21	MATHEFF	STRESAGR	PV1MATH	IMAGINE
TUR	7,059	0.229	136	MATHEF21	MATHEFF	STRESAGR	MATHPERS	ST004D01T
URY	4,389	0.067	142	PV1MATH	MATHEFF	MATHEF21	STRESAGR	MATHPERS
USA	4,183	0.260	89	MATHEFF	MATHEF21	ST004D01T	PV1MATH	DISCLIM
UZB	5,063	0.185	72	MATHPERS	MATHEFF	MATHEF21	DISCLIM	CREATEFF
VNM	5,934	0.180	50	MATHPERS	EMOCOAGR	MATHEFF	DISCLIM	PROBSELF

4 TABLE S3. INITIAL UNADJUSTED IDV-CODE VS LTO-CODE SENSITIVITY (ALL FEATURES)

Table S3: Sensitivity of the initial unadjusted operational codes to an alternative cluster scheme. `IDV code` uses official individualism terciles; `LTO code` uses official long-term-orientation terciles. A W-coded rank pattern under IDV that changes to I under LTO does not reproduce under the alternative grouping and does not establish an IDV-specific psychological process.

Outcome	Code	Feature label	IDV code	LTO code	Agreement
BELONG	AGE	Age	I	I	agree
BELONG	ASSERAGR	Assertiveness	W	I	disagree
BELONG	BFMJ2	Father occupation	I	I	agree
BELONG	BMMJ1	Mother occupation	I	I	agree
BELONG	BODYIMA	Body image	I	I	agree
BELONG	BULLIED	Exposure to bullying	U	U	agree
BELONG	COGACMCO	Cognitive activation	I	I	agree
BELONG	COGACRCO	Cognitive reasoning	I	I	agree
BELONG	COOPAGR	Cooperation tendency	I	I	agree
BELONG	CREATEFF	Creative self-efficacy	I	I	agree
BELONG	CREATOPN	Creative openness	I	I	agree
BELONG	CURIOAGR	Curiosity	C	I	disagree
BELONG	CURSUPP	Current support	I	I	agree
BELONG	DISCLIM	Disciplinary climate	I	I	agree
BELONG	EMOCOAGR	Emotional control	I	I	agree
BELONG	EMPATAGR	Empathy	I	I	agree
BELONG	ESCS	Socio-economic index	I	I	agree
BELONG	EXPO21ST	Exposure 21c math	I	I	agree
BELONG	EXPOFA	Exposure formal math	I	I	agree
BELONG	EXPWB	Experienced wellbeing	I	I	agree
BELONG	FAMCON	Math familiarity	I	I	agree
BELONG	FAMSUP	Family support	I	I	agree
BELONG	FAMSUPSL	Family support self-dir.	I	I	agree
BELONG	FEELLAH	Feelings learning home	I	I	agree
BELONG	FEELSAFE	School safety	U	U	agree
BELONG	FISCED	Father education	I	I	agree
BELONG	GRADE	Grade vs modal	I	I	agree
BELONG	GROSAGR	Growth mindset	I	I	agree
BELONG	HISEI	Parent ISEI	I	I	agree
BELONG	HOMEPOS	Home possessions	I	I	agree
BELONG	ICTAVHOM	ICT use at home	I	I	agree
BELONG	ICTAVSCH	ICT use at school	I	I	agree
BELONG	ICTEFFIC	ICT self-efficacy	I	I	agree
BELONG	ICTENQ	ICT inquiry use	I	I	agree
BELONG	ICTFEED	ICT feedback	I	I	agree
BELONG	ICTHOME	ICT availability home	I	I	agree
BELONG	ICTINFO	ICT info-seeking	I	I	agree
BELONG	ICTOUT	ICT out-of-school use	I	I	agree
BELONG	ICTQUAL	ICT quality	I	I	agree
BELONG	ICTREG	ICT regulation	I	I	agree
BELONG	ICTRES	ICT resources	I	I	agree
BELONG	ICTSCH	ICT availability school	I	I	agree
BELONG	ICTSUBJ	ICT subject lessons	I	I	agree

Outcome	Code	Feature label	IDV code	LTO code	Agreement
BELONG	ICTWKDY	ICT weekday	I	I	agree
BELONG	ICTWKEND	ICT weekend	I	I	agree
BELONG	IMAGINE	Imagination	I	I	agree
BELONG	IMMIG	Immigration status	I	I	agree
BELONG	INFOSEEK	Information seeking	I	I	agree
BELONG	LANGN	Language at home	I	I	agree
BELONG	LIFESAT	Life satisfaction	I	I	agree
BELONG	MATHEF21	Math self-efficacy 21st-c.	I	I	agree
BELONG	MATHEFF	Math self-efficacy	I	I	agree
BELONG	MATHPERS	Math persistence	I	I	agree
BELONG	MISCED	Mother education	I	I	agree
BELONG	MISSSC	Missing school	I	I	agree
BELONG	OPENART	Openness to art	W	I	disagree
BELONG	PARINVOL	Parental involvement	I	I	agree
BELONG	PASCHPOL	School parent policy	I	I	agree
BELONG	PERSEVAGR	Perseverance	I	I	agree
BELONG	PROBSELF	Self-regulation problems	I	I	agree
BELONG	PSYCHSYM	Psychosomatic symptoms	I	I	agree
BELONG	PV1MATH	Math achievement (PV1)	I	I	agree
BELONG	PV1READ	Reading achievement (PV1)	I	I	agree
BELONG	PV1SCIE	Science achievement (PV1)	I	I	agree
BELONG	RELATST	Teacher–student relations	U	U	agree
BELONG	SCHRISK	School risk	I	I	agree
BELONG	SDLEFF	SDL self-efficacy	I	I	agree
BELONG	SKIPPING	Class skipping	I	I	agree
BELONG	SOCCON	Social connection	I	I	agree
BELONG	SOCONPA	Social conn. parents	I	I	agree
BELONG	ST004D01T	Gender	I	I	agree
BELONG	STRESAGR	Stress resistance	W	I	disagree
BELONG	TEACHSUP	Math teacher support	I	I	agree
ANXMAT	AGE	Age	I	I	agree
ANXMAT	ASSERAGR	Assertiveness	I	I	agree
ANXMAT	BFMJ2	Father occupation	I	I	agree
ANXMAT	BMMJ1	Mother occupation	I	I	agree
ANXMAT	BODYIMA	Body image	I	I	agree
ANXMAT	BULLIED	Exposure to bullying	I	I	agree
ANXMAT	COGACMCO	Cognitive activation	I	I	agree
ANXMAT	COGACRCO	Cognitive reasoning	I	I	agree
ANXMAT	COOPAGR	Cooperation tendency	I	I	agree
ANXMAT	CREATEFF	Creative self-efficacy	I	I	agree
ANXMAT	CREATOPN	Creative openness	I	I	agree
ANXMAT	CURIOAGR	Curiosity	I	I	agree
ANXMAT	CURSUPP	Current support	I	I	agree
ANXMAT	DISCLIM	Disciplinary climate	I	I	agree
ANXMAT	EMOCOAGR	Emotional control	I	I	agree
ANXMAT	EMPATAGR	Empathy	I	I	agree
ANXMAT	ESCS	Socio-economic index	I	I	agree
ANXMAT	EXPO21ST	Exposure 21c math	I	I	agree
ANXMAT	EXPOFA	Exposure formal math	I	I	agree
ANXMAT	EXPWB	Experienced wellbeing	I	I	agree
ANXMAT	FAMCON	Math familiarity	I	I	agree
ANXMAT	FAMSUP	Family support	I	I	agree
ANXMAT	FAMSUPSL	Family support self-dir.	I	I	agree

Outcome	Code	Feature label	IDV code	LTO code	Agreement
ANXMAT	FEELLAH	Feelings learning home	I	I	agree
ANXMAT	FEELSAFE	School safety	I	I	agree
ANXMAT	FISCED	Father education	I	I	agree
ANXMAT	GRADE	Grade vs modal	I	I	agree
ANXMAT	GROSAGR	Growth mindset	U	U	agree
ANXMAT	HISEI	Parent ISEI	I	I	agree
ANXMAT	HOMEPOS	Home possessions	I	I	agree
ANXMAT	ICTAVHOM	ICT use at home	I	I	agree
ANXMAT	ICTAVSCH	ICT use at school	I	I	agree
ANXMAT	ICTEFFIC	ICT self-efficacy	I	I	agree
ANXMAT	ICTENQ	ICT inquiry use	I	I	agree
ANXMAT	ICTFEED	ICT feedback	I	I	agree
ANXMAT	ICTHOME	ICT availability home	I	I	agree
ANXMAT	ICTINFO	ICT info-seeking	I	I	agree
ANXMAT	ICTOUT	ICT out-of-school use	I	I	agree
ANXMAT	ICTQUAL	ICT quality	I	I	agree
ANXMAT	ICTREG	ICT regulation	I	I	agree
ANXMAT	ICTRES	ICT resources	I	I	agree
ANXMAT	ICTSCH	ICT availability school	I	I	agree
ANXMAT	ICTSUBJ	ICT subject lessons	I	I	agree
ANXMAT	ICTWKDY	ICT weekday	I	I	agree
ANXMAT	ICTWKEND	ICT weekend	I	I	agree
ANXMAT	IMAGINE	Imagination	I	I	agree
ANXMAT	IMMIG	Immigration status	I	I	agree
ANXMAT	INFOSEEK	Information seeking	I	I	agree
ANXMAT	LANGN	Language at home	I	I	agree
ANXMAT	LIFESAT	Life satisfaction	I	I	agree
ANXMAT	MATHEF21	Math self-efficacy 21st-c.	U	U	agree
ANXMAT	MATHEFF	Math self-efficacy	U	U	agree
ANXMAT	MATHPERS	Math persistence	U	U	agree
ANXMAT	MISCED	Mother education	I	I	agree
ANXMAT	MISSSC	Missing school	I	I	agree
ANXMAT	OPENART	Openness to art	I	I	agree
ANXMAT	PARINVOL	Parental involvement	I	I	agree
ANXMAT	PASCHPOL	School parent policy	I	I	agree
ANXMAT	PERSEVAGR	Perseverance	I	I	agree
ANXMAT	PROBSELF	Self-regulation problems	I	I	agree
ANXMAT	PSYCHSYM	Psychosomatic symptoms	I	I	agree
ANXMAT	PV1MATH	Math achievement (PV1)	U	U	agree
ANXMAT	PV1READ	Reading achievement (PV1)	I	I	agree
ANXMAT	PV1SCIE	Science achievement (PV1)	I	I	agree
ANXMAT	RELATST	Teacher–student relations	I	I	agree
ANXMAT	SCHRISK	School risk	I	I	agree
ANXMAT	SDLEFF	SDL self-efficacy	I	I	agree
ANXMAT	SKIPPING	Class skipping	I	I	agree
ANXMAT	SOCCON	Social connection	I	I	agree
ANXMAT	SOCONPA	Social conn. parents	I	I	agree
ANXMAT	ST004D01T	Gender	I	I	agree
ANXMAT	STRESAGR	Stress resistance	W	I	disagree
ANXMAT	TEACHSUP	Math teacher support	W	I	disagree

5 TABLE S4. PER-ECONOMY CRONBACH'S α

Table S4: A limited complete-case descriptive diagnostic for the BELONG and ANXMAT six-item scales. Only 4 economies provided enough complete six-item responses to calculate Cronbach's α for both outcomes; the remaining economies are shown to document the balanced incomplete block design constraint. The available median values ($\alpha = .78$ for BELONG and $.85$ for ANXMAT) describe those economies only. This diagnostic is not evidence of measurement invariance or cross-economy equivalence.

Economy	n (BELONG)	α BELONG	n (ANXMAT)	α ANXMAT
ALB	0	-	0	-
ARE	0	-	0	-
ARG	0	-	0	-
AUS	1	-	0	-
AUT	0	-	0	-
BEL	0	-	0	-
BGR	0	-	0	-
BRA	0	-	0	-
BRN	0	-	0	-
CAN	0	-	0	-
CHE	0	-	0	-
CHL	0	-	0	-
COL	0	-	0	-
CRI	0	-	0	-
CZE	0	-	0	-
DEU	0	-	0	-
DNK	0	-	0	-
DOM	0	-	0	-
ESP	0	-	0	-
EST	0	-	0	-
FIN	0	-	0	-
FRA	0	-	0	-
GBR	0	-	0	-
GEO	0	-	0	-
GRC	0	-	0	-
GTM	4,572	0.821	3,618	0.855
HKG	0	-	0	-
HRV	0	-	0	-
HUN	0	-	0	-
IDN	0	-	0	-
IRL	0	-	0	-
ISL	0	-	0	-
ISR	0	-	0	-
ITA	0	-	0	-
JAM	0	-	0	-
JOR	0	-	0	-
JPN	0	-	0	-
KAZ	0	-	0	-
KHM	4,600	0.664	4,053	0.804
KOR	0	-	0	-
KSV	0	-	0	-
LTU	0	-	0	-
LVA	0	-	0	-
MAC	0	-	0	-

**Economy**	**n (BELONG)**	**α BELONG**	**n (ANXMAT)**	**α ANXMAT**
MAR	0	-	0	-
MDA	0	-	0	-
MEX	0	-	0	-
MKD	0	-	0	-
MLT	0	-	0	-
MNE	0	-	0	-
MNG	0	-	0	-
MYS	0	-	0	-
NLD	0	-	0	-
NOR	0	-	0	-
NZL	0	-	0	-
PAN	0	-	0	-
PER	2	-	2	-
PHL	0	-	0	-
POL	0	-	0	-
PRT	0	-	0	-
PRY	4,214	0.827	3,660	0.842
PSE	0	-	0	-
QAT	0	-	0	-
QAZ	0	-	0	-
QUR	0	-	0	-
ROU	0	-	0	-
SAU	0	-	0	-
SGP	0	-	0	-
SLV	0	-	0	-
SRB	0	-	0	-
SVK	0	-	0	-
SVN	0	-	0	-
SWE	0	-	0	-
TAP	0	-	0	-
THA	0	-	0	-
TUR	0	-	0	-
URY	0	-	0	-
USA	0	-	0	-
UZB	0	-	0	-
VNM	5,926	0.729	5,875	0.885

6 TABLE S5. BOOTSTRAP 95% CI ON INITIAL UNADJUSTED CLUSTER MEAN RANK

6.1 S5a. Outcome: BELONG

Table S5: Bootstrap 95% confidence intervals on initial unadjusted cluster mean ranks for BELONG, all features. Cell format: `point [lower, upper]` where the point is the cluster mean rank across economies in the cluster (1 = most important) and the bracketed bounds are the 2.5th and 97.5th percentiles of 1,000 economy-level resamples within cluster. Features are sorted by their best (lowest) cluster mean rank; features assigned the operational U code under the initial rule appear at top.

Code	Feature label	low IDV [95% CI]	mid IDV [95% CI]	high IDV [95% CI]
FEELSAFE	School safety	3.0 [1.2, 6.4]	1.5 [1.2, 2.0]	10.0 [2.8, 19.4]
BULLIED	Exposure to bullying	3.1 [2.6, 3.5]	3.1 [2.5, 3.8]	2.3 [2.0, 2.6]
RELATST	Teacher–student relations	9.6 [3.9, 17.0]	3.6 [3.0, 4.2]	6.0 [3.7, 10.0]
STRESAGR	Stress resistance	44.7 [39.6, 49.2]	18.8 [10.7, 27.8]	12.0 [6.8, 18.7]
ASSERAGR	Assertiveness	42.1 [35.3, 47.9]	13.8 [7.3, 23.3]	12.2 [7.1, 18.8]
CURIOAGR	Curiosity	12.2 [8.3, 17.2]	20.6 [15.4, 26.1]	29.8 [25.0, 35.3]
EMOCOAGR	Emotional control	19.2 [12.1, 26.8]	13.6 [10.7, 17.1]	23.1 [16.1, 31.1]
CREATEFF	Creative self-efficacy	14.1 [10.0, 19.1]	14.7 [11.2, 18.6]	22.4 [17.0, 28.3]
OPENART	Openness to art	26.0 [21.5, 31.2]	17.5 [13.5, 22.5]	14.9 [9.9, 20.8]
DISCLIM	Disciplinary climate	15.5 [12.0, 19.8]	20.2 [16.5, 24.2]	20.5 [16.2, 25.3]
COOPAGR	Cooperation tendency	39.5 [31.8, 47.0]	22.4 [12.3, 33.5]	16.0 [7.2, 25.5]
FAMSUP	Family support	17.6 [12.6, 23.6]	16.9 [13.4, 20.3]	17.8 [13.8, 22.2]
PV1READ	Reading achievement (PV1)	17.1 [13.0, 21.6]	26.5 [21.3, 31.7]	26.1 [21.2, 30.5]
PV1SCIE	Science achievement (PV1)	17.1 [13.7, 20.5]	28.2 [21.0, 35.3]	22.2 [18.4, 26.7]
PERSEVAGR	Perseverance	36.2 [28.0, 43.8]	18.9 [12.8, 26.9]	19.5 [14.1, 26.1]
PV1MATH	Math achievement (PV1)	19.0 [14.9, 23.4]	33.4 [27.5, 39.8]	35.3 [30.6, 40.3]
PROBSELF	Self-regulation problems	21.3 [17.1, 25.9]	27.2 [21.9, 33.4]	24.5 [19.3, 29.9]
ST004D01T	Gender	31.9 [25.3, 38.3]	42.2 [33.9, 49.6]	22.0 [14.2, 30.0]
HOMEPOS	Home possessions	22.3 [17.6, 27.1]	26.1 [20.6, 31.9]	29.1 [24.5, 34.5]
ICTREG	ICT regulation	34.5 [30.1, 38.9]	32.3 [26.2, 37.8]	23.2 [17.2, 29.7]
EMPATAGR	Empathy	43.3 [36.8, 49.3]	23.3 [17.1, 30.5]	28.8 [22.4, 35.8]
MATHPERS	Math persistence	23.4 [19.2, 28.4]	30.8 [25.5, 36.2]	24.9 [20.2, 29.9]
ICTQUAL	ICT quality	34.5 [29.2, 39.2]	26.8 [21.5, 32.1]	23.7 [18.4, 30.1]
ICTRES	ICT resources	24.0 [18.0, 31.0]	34.8 [25.7, 43.3]	41.1 [32.0, 49.7]
GROSAGR	Growth mindset	24.0 [20.2, 28.2]	29.2 [24.2, 34.8]	33.1 [28.6, 38.2]
FISCED	Father education	25.0 [20.9, 29.4]	38.8 [32.9, 45.0]	34.9 [29.0, 41.0]
FAMSUPSL	Family support self-dir.	25.4 [20.8, 30.4]	29.0 [24.9, 33.7]	30.0 [24.4, 35.7]
TEACHSUP	Math teacher support	26.3 [20.5, 32.6]	39.8 [33.8, 45.5]	37.0 [31.7, 41.9]
SDLEFF	SDL self-efficacy	26.4 [21.4, 31.2]	32.5 [27.7, 37.4]	32.3 [26.1, 39.0]
MATHEF21	Math self-efficacy 21st-c.	27.8 [24.4, 31.7]	32.1 [27.7, 36.6]	32.0 [27.5, 36.3]
MISCED	Mother education	27.8 [22.5, 33.6]	39.2 [33.5, 44.6]	38.3 [32.8, 43.8]
MATHEFF	Math self-efficacy	27.8 [22.8, 32.5]	28.7 [23.6, 33.6]	28.6 [24.1, 33.0]
COGACMCO	Cognitive activation	27.9 [24.4, 31.6]	28.7 [24.1, 33.0]	29.0 [25.0, 33.1]
BFMJ2	Father occupation	28.2 [24.0, 32.8]	31.5 [27.3, 35.8]	34.2 [30.4, 38.1]
ESCS	Socio-economic index	28.4 [24.1, 33.0]	34.8 [28.2, 41.0]	30.2 [25.6, 35.1]
COGACRCO	Cognitive reasoning	28.8 [25.0, 32.5]	30.5 [26.0, 34.8]	34.3 [30.6, 38.2]
EXPOFA	Exposure formal math	29.4 [25.4, 34.1]	35.9 [31.6, 40.4]	33.7 [29.0, 38.2]
HISEI	Parent ISEI	29.8 [25.4, 34.3]	40.2 [35.5, 44.5]	37.5 [33.5, 41.3]
INFOSEEK	Information seeking	30.1 [26.3, 33.4]	31.5 [26.1, 37.3]	37.5 [32.1, 42.6]
EXPO21ST	Exposure 21c math	30.6 [27.0, 34.4]	34.6 [30.3, 38.5]	36.1 [32.6, 39.4]
IMAGINE	Imagination	34.4 [30.3, 38.5]	31.8 [27.3, 36.3]	38.4 [33.0, 43.9]

Code	Feature label	low IDV [95% CI]	mid IDV [95% CI]	high IDV [95% CI]
SCHRISK	School risk	32.3 [26.7, 38.2]	41.5 [36.0, 47.0]	43.7 [37.2, 49.5]
BMMJ1	Mother occupation	32.6 [28.0, 37.4]	34.3 [28.6, 40.0]	32.5 [27.8, 37.0]
ICTEFFIC	ICT self-efficacy	32.5 [27.7, 37.5]	32.5 [26.8, 38.1]	33.7 [27.8, 39.2]
FEELLAH	Feelings learning home	33.2 [29.3, 37.7]	40.5 [35.4, 45.8]	41.8 [36.4, 47.0]
ICTINFO	ICT info-seeking	37.6 [32.4, 42.1]	33.5 [28.4, 38.6]	37.8 [32.9, 42.2]
ICTWKEND	ICT weekend	39.9 [35.9, 43.3]	34.8 [30.0, 39.8]	36.2 [31.5, 41.0]
ICTFEED	ICT feedback	41.4 [37.0, 45.6]	39.2 [34.4, 43.8]	35.8 [30.4, 41.1]
FAMCON	Math familiarity	42.3 [38.2, 45.9]	35.8 [30.3, 42.0]	37.1 [32.9, 41.2]
ICTSUBJ	ICT subject lessons	37.0 [33.0, 40.5]	37.3 [33.7, 41.2]	39.5 [34.3, 44.5]
MISSSC	Missing school	37.4 [32.2, 42.6]	49.5 [44.0, 54.8]	50.6 [45.8, 54.6]
ICTWKDY	ICT weekday	41.5 [38.4, 44.3]	40.2 [35.6, 44.2]	37.8 [33.0, 42.4]
ICTOUT	ICT out-of-school use	38.4 [34.3, 42.6]	39.2 [35.3, 43.1]	38.7 [34.0, 43.1]
ICTENQ	ICT inquiry use	41.6 [38.5, 45.2]	39.4 [35.3, 43.2]	38.5 [33.7, 43.0]
LANGN	Language at home	40.1 [34.0, 45.7]	45.2 [37.5, 52.2]	39.0 [31.0, 46.8]
SOCCON	Social connection	39.3 [33.3, 44.9]	52.2 [45.5, 57.7]	46.9 [38.7, 54.0]
PSYCHSYM	Psychosomatic symptoms	39.5 [33.6, 44.9]	51.4 [43.6, 57.6]	46.4 [37.5, 53.9]
LIFESAT	Life satisfaction	39.5 [32.5, 45.8]	50.5 [41.7, 57.4]	44.7 [34.6, 53.5]
EXPWB	Experienced wellbeing	39.7 [33.8, 45.2]	52.4 [45.8, 57.8]	46.0 [37.8, 53.4]
BODYIMA	Body image	40.2 [33.6, 46.0]	51.2 [43.2, 57.5]	45.9 [36.8, 53.9]
GRADE	Grade vs modal	40.6 [35.4, 45.8]	55.2 [51.6, 58.6]	52.1 [47.4, 56.3]
IMMIG	Immigration status	46.6 [43.0, 50.6]	54.4 [50.5, 57.7]	40.7 [33.6, 47.5]
AGE	Age	41.2 [37.4, 45.1]	43.0 [37.2, 48.7]	46.3 [42.8, 49.8]
SOCONPA	Social conn. parents	41.4 [35.8, 46.7]	56.8 [54.2, 59.1]	50.6 [44.7, 55.6]
CURSUPP	Current support	41.9 [37.0, 46.6]	51.6 [45.4, 57.0]	56.6 [53.5, 59.2]
PARINVOL	Parental involvement	42.2 [37.6, 46.5]	52.0 [47.6, 55.9]	54.3 [50.5, 57.6]
ICTSCH	ICT availability school	43.4 [40.0, 46.9]	53.1 [50.4, 55.8]	51.0 [48.1, 53.5]
CREATOPN	Creative openness	43.7 [39.5, 47.2]	46.9 [40.1, 52.9]	53.0 [47.9, 57.2]
PASCHPOL	School parent policy	43.8 [40.0, 47.3]	48.6 [42.7, 53.7]	54.7 [51.2, 57.8]
SKIPPING	Class skipping	44.2 [40.4, 48.0]	55.4 [51.5, 58.7]	53.3 [50.4, 56.0]
ICTHOME	ICT availability home	46.3 [43.2, 49.4]	55.2 [52.5, 57.7]	53.4 [50.7, 55.7]
ICTAVHOM	ICT use at home	46.5 [43.7, 49.6]	56.1 [53.5, 58.4]	55.1 [52.1, 57.7]
ICTAVSCH	ICT use at school	47.0 [44.0, 50.1]	55.8 [52.9, 58.3]	53.2 [49.9, 55.9]

6.2 S5b. Outcome: ANXMAT

Table S5: Bootstrap 95% confidence intervals on initial unadjusted cluster mean ranks for ANXMAT, all features. Cell format: `point [lower, upper]` where the point is the cluster mean rank across economies in the cluster (1 = most important) and the bracketed bounds are the 2.5th and 97.5th percentiles of 1,000 economy-level resamples within cluster. Features are sorted by their best (lowest) cluster mean rank; features assigned the operational U code under the initial rule appear at top.

Code	Feature label	low IDV [95% CI]	mid IDV [95% CI]	high IDV [95% CI]
MATHEFF	Math self-efficacy	5.8 [1.6, 11.6]	2.0 [1.5, 2.5]	1.5 [1.2, 1.8]
MATHEF21	Math self-efficacy 21st-c.	6.7 [2.7, 11.8]	2.6 [2.0, 3.2]	2.9 [2.5, 3.3]
PV1MATH	Math achievement (PV1)	6.7 [4.9, 9.1]	6.4 [4.7, 8.5]	4.2 [3.4, 5.0]
MATHPERS	Math persistence	4.6 [2.5, 7.5]	5.3 [4.0, 7.0]	12.5 [9.2, 16.3]
GROSAGR	Growth mindset	11.6 [8.5, 15.5]	8.5 [7.1, 10.2]	12.6 [8.2, 18.0]
STRESAGR	Stress resistance	42.6 [36.0, 48.5]	15.2 [6.9, 24.6]	9.6 [3.9, 17.3]
EMOCOAGR	Emotional control	16.3 [8.8, 25.3]	10.3 [8.2, 13.5]	20.4 [13.5, 28.3]
EXPO21ST	Exposure 21c math	12.7 [10.6, 14.9]	17.4 [13.6, 21.3]	19.2 [16.0, 23.3]
PROBSELF	Self-regulation problems	16.7 [12.8, 21.3]	14.3 [10.1, 19.2]	13.3 [8.8, 18.5]
DISCLIM	Disciplinary climate	13.7 [10.4, 17.5]	21.8 [17.5, 26.3]	22.2 [16.8, 27.4]
TEACHSUP	Math teacher support	27.8 [21.5, 33.7]	24.8 [18.2, 31.3]	14.1 [10.4, 18.4]
IMAGINE	Imagination	22.2 [15.5, 29.3]	14.8 [11.0, 19.7]	29.9 [22.2, 37.5]
ST004D01T	Gender	16.0 [9.5, 23.2]	31.6 [24.0, 39.1]	19.9 [13.0, 27.7]
BULLIED	Exposure to bullying	16.0 [11.6, 21.0]	27.6 [20.9, 34.5]	24.4 [18.4, 30.5]
EXPOFA	Exposure formal math	16.2 [13.4, 19.4]	21.4 [17.6, 25.6]	23.1 [19.7, 26.3]
FAMCON	Math familiarity	31.7 [24.1, 39.0]	22.0 [14.3, 30.0]	17.7 [12.8, 23.8]
CREATEFF	Creative self-efficacy	19.5 [15.4, 23.9]	20.6 [16.8, 24.4]	22.2 [17.1, 27.8]
PV1READ	Reading achievement (PV1)	19.7 [15.3, 24.4]	27.9 [22.5, 33.5]	30.6 [25.7, 35.0]
EMPATAGR	Empathy	43.6 [37.5, 49.5]	19.8 [14.5, 26.6]	25.1 [18.7, 32.0]
PV1SCIE	Science achievement (PV1)	20.4 [15.9, 25.5]	31.5 [26.0, 37.3]	26.0 [20.5, 31.7]
COGACMCO	Cognitive activation	22.8 [18.4, 28.0]	21.0 [17.0, 25.4]	25.3 [22.2, 29.0]
RELATST	Teacher–student relations	24.8 [19.6, 30.8]	27.0 [22.7, 31.6]	22.2 [18.2, 26.8]
PERSEVAGR	Perseverance	37.6 [30.7, 44.3]	26.8 [21.4, 33.2]	22.6 [17.8, 28.4]
OPENART	Openness to art	24.8 [21.2, 28.9]	23.1 [19.2, 26.7]	23.5 [18.8, 28.9]
CURIOAGR	Curiosity	23.5 [20.2, 27.0]	27.8 [23.9, 31.8]	31.5 [27.4, 35.6]
ICTOUT	ICT out-of-school use	34.9 [30.2, 39.1]	24.8 [19.5, 30.1]	23.8 [17.7, 30.2]
FAMSUP	Family support	23.9 [19.8, 29.0]	28.6 [25.0, 32.4]	32.0 [28.1, 36.4]
COGACRCO	Cognitive reasoning	24.0 [20.0, 27.9]	27.6 [23.1, 32.5]	31.6 [27.1, 35.8]
SDLEFF	SDL self-efficacy	24.3 [20.5, 28.6]	36.0 [32.6, 39.5]	35.1 [30.6, 39.7]
INFOSEEK	Information seeking	26.0 [23.0, 29.2]	30.1 [25.5, 35.7]	29.4 [23.1, 35.3]
FEELSAFE	School safety	30.0 [25.5, 35.1]	30.1 [24.5, 36.1]	32.0 [25.8, 38.6]
FEELLAH	Feelings learning home	30.3 [25.6, 35.0]	40.3 [35.8, 44.5]	42.9 [38.5, 47.3]
FAMSUPSL	Family support self-dir.	31.0 [26.2, 36.0]	35.3 [31.6, 38.8]	36.0 [32.2, 40.0]
ASSERAGR	Assertiveness	43.6 [39.0, 48.4]	31.1 [24.9, 37.6]	31.5 [27.3, 35.8]
ICTINFO	ICT info-seeking	37.5 [31.7, 42.6]	34.0 [29.7, 38.0]	31.4 [26.1, 36.9]
HOMEPOS	Home possessions	32.3 [27.5, 36.9]	38.7 [34.5, 42.4]	39.7 [35.3, 43.4]
FISCED	Father education	32.3 [27.7, 36.9]	44.1 [38.8, 48.9]	39.2 [33.6, 44.4]
ICTENQ	ICT inquiry use	38.2 [34.4, 42.0]	32.5 [28.2, 37.0]	38.9 [34.8, 42.8]
ICTREG	ICT regulation	35.0 [30.1, 39.4]	32.6 [28.7, 36.8]	36.8 [32.3, 40.8]
MISCED	Mother education	33.1 [28.4, 38.3]	41.8 [36.4, 46.3]	39.5 [33.6, 44.5]
ICTQUAL	ICT quality	36.0 [31.5, 40.0]	33.5 [29.3, 38.0]	36.8 [32.8, 40.9]
ICTWKEND	ICT weekend	39.8 [35.3, 43.8]	34.3 [30.8, 38.0]	35.9 [30.8, 40.8]
ICTWKDY	ICT weekday	38.7 [35.0, 42.2]	36.8 [32.2, 41.9]	34.8 [29.9, 39.4]
ICTSUBJ	ICT subject lessons	35.2 [30.8, 39.6]	37.6 [32.7, 42.3]	36.0 [30.3, 41.6]

Code	Feature label	low IDV [95% CI]	mid IDV [95% CI]	high IDV [95% CI]
BFMJ2	Father occupation	35.7 [30.9, 40.9]	40.2 [36.7, 43.6]	35.3 [31.1, 39.7]
ESCS	Socio-economic index	36.0 [31.5, 40.5]	43.0 [39.2, 46.6]	41.0 [37.0, 44.9]
HISEI	Parent ISEI	36.0 [31.6, 40.6]	41.2 [37.1, 45.2]	42.6 [39.5, 45.7]
PSYCHSYM	Psychosomatic symptoms	36.5 [28.8, 43.8]	50.2 [42.6, 56.3]	44.9 [35.4, 53.5]
ICTRES	ICT resources	36.5 [31.2, 42.0]	44.0 [38.0, 50.1]	49.6 [44.5, 54.4]
BMMJ1	Mother occupation	36.5 [32.4, 40.7]	38.0 [34.0, 41.8]	40.9 [37.6, 44.0]
ICTFEED	ICT feedback	38.5 [34.8, 42.5]	37.1 [32.8, 41.3]	38.1 [33.7, 42.1]
ICTEFFIC	ICT self-efficacy	38.5 [34.1, 42.9]	38.3 [34.3, 42.1]	39.0 [34.5, 43.3]
COOPAGR	Cooperation tendency	41.5 [35.2, 47.9]	39.0 [33.1, 45.3]	38.6 [33.7, 44.0]
SCHRISK	School risk	39.2 [35.2, 43.7]	46.5 [42.5, 50.4]	47.6 [42.3, 51.8]
BODYIMA	Body image	39.7 [32.3, 46.0]	51.7 [45.8, 56.6]	46.8 [38.9, 53.8]
AGE	Age	39.8 [35.5, 44.1]	49.8 [46.8, 52.5]	47.2 [43.3, 50.5]
CURSUPP	Current support	41.9 [37.0, 46.1]	52.0 [47.5, 56.4]	56.2 [53.5, 58.6]
SOCCON	Social connection	42.0 [36.2, 47.1]	52.8 [47.8, 57.0]	51.0 [45.8, 55.7]
LANGN	Language at home	42.2 [35.9, 48.3]	53.8 [48.1, 58.1]	44.7 [37.5, 50.6]
LIFESAT	Life satisfaction	42.5 [37.0, 47.2]	52.3 [46.7, 57.0]	48.7 [41.9, 54.6]
CREATOPN	Creative openness	43.3 [39.1, 47.1]	49.6 [44.8, 54.0]	54.0 [50.5, 57.2]
EXPWB	Experienced wellbeing	43.5 [39.9, 47.2]	54.8 [51.0, 58.2]	49.8 [43.6, 54.9]
PASCHPOL	School parent policy	43.5 [39.8, 46.9]	51.2 [46.7, 55.2]	53.8 [50.5, 57.0]
SKIPPING	Class skipping	44.2 [40.5, 48.3]	56.2 [53.1, 58.9]	51.3 [46.5, 55.2]
PARINVOL	Parental involvement	44.2 [41.1, 47.1]	49.9 [45.5, 54.1]	53.5 [48.8, 57.3]
ICTHOME	ICT availability home	44.3 [41.1, 47.7]	53.8 [50.7, 56.7]	52.7 [50.3, 54.7]
MISSSC	Missing school	44.8 [40.5, 49.4]	57.4 [54.2, 60.0]	54.7 [51.8, 57.5]
ICTSCH	ICT availability school	44.8 [41.7, 48.0]	51.9 [49.0, 54.6]	50.9 [47.9, 53.5]
ICTAVHOM	ICT use at home	45.1 [41.2, 49.1]	56.1 [53.1, 58.7]	54.2 [51.5, 56.6]
SOCONPA	Social conn. parents	45.3 [41.9, 48.9]	53.9 [49.5, 57.5]	52.0 [48.2, 55.9]
GRADE	Grade vs modal	45.8 [41.4, 50.1]	54.7 [51.0, 57.9]	48.0 [41.5, 52.9]
ICTAVSCH	ICT use at school	46.8 [43.4, 50.5]	55.7 [52.8, 58.1]	54.3 [51.7, 56.6]
IMMIG	Immigration status	47.5 [43.4, 51.8]	55.2 [51.0, 58.6]	48.2 [43.5, 52.7]

6.3 S5c. Pairwise cross-cluster rank agreement

Table S5: Pairwise cross-cluster Spearman rank correlations and permutation-null reference intervals from the initial unadjusted analysis. Observed ρ values compare the top-20 cluster rank profiles. Null summaries were obtained by permuting economy cluster labels; the last two columns are the 2.5th and 97.5th percentiles of the corresponding null distribution.

Outcome	Cluster comparison	Observed ρ	Null mean	Null 2.5%	Null 97.5%
BELONG	low vs. mid IDV	0.316	0.703	0.462	0.869
BELONG	low vs. high IDV	0.070	0.705	0.450	0.867
BELONG	mid vs. high IDV	0.652	0.704	0.443	0.875
ANXMAT	low vs. mid IDV	0.475	0.730	0.565	0.870
ANXMAT	low vs. high IDV	0.435	0.728	0.568	0.876
ANXMAT	mid vs. high IDV	0.580	0.738	0.590	0.874

7 TABLE S6. STRICTER ALTERNATIVE RULES VS. INITIAL UNADJUSTED OPERATIONAL CODES

Table S6: Comparison of a stricter alternative rule set with the initial unadjusted operational codes. The stricter alternative uses median rank, requires cross-cluster $\rho \geq 0.70$ for U, and requires a rank difference ≥ 30 for W/C. The stricter-rule counts are BELONG: U=0, W=4, C=0, I=69; ANXMAT: U=0, W=1, C=0, I=72. Neither rule set is treated as a natural psychological classification.

Outcome	Code	Feature label	Stricter-rule code	Initial unadjusted code
BELONG	AGE	Age	I	I
BELONG	ASSERAGR	Assertiveness	W	W
BELONG	BFMJ2	Father occupation	I	I
BELONG	BMMJ1	Mother occupation	I	I
BELONG	BODYIMA	Body image	I	I
BELONG	BULLIED	Exposure to bullying	I	U
BELONG	COGACMCO	Cognitive activation	I	I
BELONG	COGACRCO	Cognitive reasoning	I	I
BELONG	COOPAGR	Cooperation tendency	I	I
BELONG	CREATEFF	Creative self-efficacy	I	I
BELONG	CREATOPN	Creative openness	I	I
BELONG	CURIOAGR	Curiosity	I	C
BELONG	CURSUPP	Current support	I	I
BELONG	DISCLIM	Disciplinary climate	I	I
BELONG	EMOCOAGR	Emotional control	I	I
BELONG	EMPATAGR	Empathy	I	I
BELONG	ESCS	Socio-economic index	I	I
BELONG	EXPO21ST	Exposure 21c math	I	I
BELONG	EXPOFA	Exposure formal math	I	I
BELONG	EXPWB	Experienced wellbeing	I	I
BELONG	FAMCON	Math familiarity	I	I
BELONG	FAMSUP	Family support	I	I
BELONG	FAMSUPSL	Family support self-dir.	I	I
BELONG	FEELLAH	Feelings learning home	I	I
BELONG	FEELSAFE	School safety	I	U
BELONG	FISCED	Father education	I	I
BELONG	GRADE	Grade vs modal	I	I
BELONG	GROSAGR	Growth mindset	I	I
BELONG	HISEI	Parent ISEI	I	I
BELONG	HOMEPOS	Home possessions	I	I
BELONG	ICTAVHOM	ICT use at home	I	I
BELONG	ICTAVSCH	ICT use at school	I	I
BELONG	ICTEFFIC	ICT self-efficacy	I	I
BELONG	ICTENQ	ICT inquiry use	I	I
BELONG	ICTFEED	ICT feedback	I	I
BELONG	ICTHOME	ICT availability home	I	I
BELONG	ICTINFO	ICT info-seeking	I	I
BELONG	ICTOUT	ICT out-of-school use	I	I
BELONG	ICTQUAL	ICT quality	I	I
BELONG	ICTREG	ICT regulation	W	I
BELONG	ICTRES	ICT resources	I	I
BELONG	ICTSCH	ICT availability school	I	I
BELONG	ICTSUBJ	ICT subject lessons	I	I

Outcome	Code	Feature label	Stricter-rule code	Initial unadjusted code
BELONG	ICTWKDY	ICT weekday	I	I
BELONG	ICTWKEND	ICT weekend	I	I
BELONG	IMAGINE	Imagination	I	I
BELONG	IMMIG	Immigration status	I	I
BELONG	INFOSEEK	Information seeking	I	I
BELONG	LANGN	Language at home	I	I
BELONG	LIFESAT	Life satisfaction	I	I
BELONG	MATHEF21	Math self-efficacy 21st-c.	I	I
BELONG	MATHEFF	Math self-efficacy	I	I
BELONG	MATHPERS	Math persistence	I	I
BELONG	MISCED	Mother education	I	I
BELONG	MISSSC	Missing school	I	I
BELONG	OPENART	Openness to art	I	W
BELONG	PARINVOL	Parental involvement	I	I
BELONG	PASCHPOL	School parent policy	I	I
BELONG	PERSEVAGR	Perseverance	W	I
BELONG	PROBSELF	Self-regulation problems	I	I
BELONG	PSYCHSYM	Psychosomatic symptoms	I	I
BELONG	PV1MATH	Math achievement (PV1)	I	I
BELONG	PV1READ	Reading achievement (PV1)	I	I
BELONG	PV1SCIE	Science achievement (PV1)	I	I
BELONG	RELATST	Teacher–student relations	I	U
BELONG	SCHRISK	School risk	I	I
BELONG	SDLEFF	SDL self-efficacy	I	I
BELONG	SKIPPING	Class skipping	I	I
BELONG	SOCCON	Social connection	I	I
BELONG	SOCONPA	Social conn. parents	I	I
BELONG	ST004D01T	Gender	I	I
BELONG	STRESAGR	Stress resistance	W	W
BELONG	TEACHSUP	Math teacher support	I	I
ANXMAT	AGE	Age	I	I
ANXMAT	ASSERAGR	Assertiveness	I	I
ANXMAT	BFMJ2	Father occupation	I	I
ANXMAT	BMMJ1	Mother occupation	I	I
ANXMAT	BODYIMA	Body image	I	I
ANXMAT	BULLIED	Exposure to bullying	I	I
ANXMAT	COGACMCO	Cognitive activation	I	I
ANXMAT	COGACRCO	Cognitive reasoning	I	I
ANXMAT	COOPAGR	Cooperation tendency	I	I
ANXMAT	CREATEFF	Creative self-efficacy	I	I
ANXMAT	CREATOPN	Creative openness	I	I
ANXMAT	CURIOAGR	Curiosity	I	I
ANXMAT	CURSUPP	Current support	I	I
ANXMAT	DISCLIM	Disciplinary climate	I	I
ANXMAT	EMOCOAGR	Emotional control	I	I
ANXMAT	EMPATAGR	Empathy	I	I
ANXMAT	ESCS	Socio-economic index	I	I
ANXMAT	EXPO21ST	Exposure 21c math	I	I
ANXMAT	EXPOFA	Exposure formal math	I	I
ANXMAT	EXPWB	Experienced wellbeing	I	I
ANXMAT	FAMCON	Math familiarity	I	I
ANXMAT	FAMSUP	Family support	I	I
ANXMAT	FAMSUPSL	Family support self-dir.	I	I

Outcome	Code	Feature label	Stricter-rule code	Initial unadjusted code
ANXMAT	FEELLAH	Feelings learning home	I	I
ANXMAT	FEELSAFE	School safety	I	I
ANXMAT	FISCED	Father education	I	I
ANXMAT	GRADE	Grade vs modal	I	I
ANXMAT	GROSAGR	Growth mindset	I	U
ANXMAT	HISEI	Parent ISEI	I	I
ANXMAT	HOMEPOS	Home possessions	I	I
ANXMAT	ICTAVHOM	ICT use at home	I	I
ANXMAT	ICTAVSCH	ICT use at school	I	I
ANXMAT	ICTEFFIC	ICT self-efficacy	I	I
ANXMAT	ICTENQ	ICT inquiry use	I	I
ANXMAT	ICTFEED	ICT feedback	I	I
ANXMAT	ICTHOME	ICT availability home	I	I
ANXMAT	ICTINFO	ICT info-seeking	I	I
ANXMAT	ICTOUT	ICT out-of-school use	I	I
ANXMAT	ICTQUAL	ICT quality	I	I
ANXMAT	ICTREG	ICT regulation	I	I
ANXMAT	ICTRES	ICT resources	I	I
ANXMAT	ICTSCH	ICT availability school	I	I
ANXMAT	ICTSUBJ	ICT subject lessons	I	I
ANXMAT	ICTWKDY	ICT weekday	I	I
ANXMAT	ICTWKEND	ICT weekend	I	I
ANXMAT	IMAGINE	Imagination	I	I
ANXMAT	IMMIG	Immigration status	I	I
ANXMAT	INFOSEEK	Information seeking	I	I
ANXMAT	LANGN	Language at home	I	I
ANXMAT	LIFESAT	Life satisfaction	I	I
ANXMAT	MATHEF21	Math self-efficacy 21st-c.	I	U
ANXMAT	MATHEFF	Math self-efficacy	I	U
ANXMAT	MATHPERS	Math persistence	I	U
ANXMAT	MISCED	Mother education	I	I
ANXMAT	MISSSC	Missing school	I	I
ANXMAT	OPENART	Openness to art	I	I
ANXMAT	PARINVOL	Parental involvement	I	I
ANXMAT	PASCHPOL	School parent policy	I	I
ANXMAT	PERSEVAGR	Perseverance	I	I
ANXMAT	PROBSELF	Self-regulation problems	I	I
ANXMAT	PSYCHSYM	Psychosomatic symptoms	I	I
ANXMAT	PV1MATH	Math achievement (PV1)	I	U
ANXMAT	PV1READ	Reading achievement (PV1)	I	I
ANXMAT	PV1SCIE	Science achievement (PV1)	I	I
ANXMAT	RELATST	Teacher–student relations	I	I
ANXMAT	SCHRISK	School risk	I	I
ANXMAT	SDLEFF	SDL self-efficacy	I	I
ANXMAT	SKIPPING	Class skipping	I	I
ANXMAT	SOCCON	Social connection	I	I
ANXMAT	SOCONPA	Social conn. parents	I	I
ANXMAT	ST004D01T	Gender	I	I
ANXMAT	STRESAGR	Stress resistance	W	W
ANXMAT	TEACHSUP	Math teacher support	I	W

8 TABLE S7. SUBNATIONAL COUNTRY-SCORE SENSITIVITY DESIGN

Table S7: Treatment of subnational samples in the country-score sensitivity. QAZ and QUR are omitted because their PISA samples are matched to national country scores. TAP is retained because the official source provides a direct Taiwan score. MAC is absent from all IDV-based analyses because no official score is available. The sensitivity reuses saved per-economy SHAP values and does not retrain the predictive models. No operational code changed for either outcome; Cohen's κ was 1.000 for the four-code and U-versus-other comparisons.

**Economy**	**Official source entry**	**IDV**	**Treatment**
QAZ	Azerbaijan	28	Omitted: Baku is assigned the Azerbaijan country score.
QUR	Ukraine	55	Omitted: the regional sample is assigned the Ukraine country score.
TAP	Taiwan	40	Retained: the official page provides a direct Taiwan score.
MAC	–	–	Not included in IDV analyses because no official score is available.

9 TABLE S8. EXPLORATORY LOW-IDV-ALIGNED RANK DIFFERENCES

9.1 S8a. Outcome: BELONG

Table S8: Exploratory low-IDV-aligned rank differences for BELONG in the initial unadjusted analysis: features whose mean rank is lower (more important) in the low-IDV than in the high-IDV cluster under a direction-only rule (BH-FDR-corrected permutation $p < 0.05$). These features do not satisfy the C-code inclusion criterion and are reported only as exploratory rank contrasts.

Code	Feature label	Mean rank low-IDV	Mean rank high-IDV	Perm. p
CURIOAGR	Curiosity	12.2	29.8	< 0.001
CREATEFF	Creative self-efficacy	14.1	22.4	0.018
PV1READ	Reading achievement (PV1)	17.1	26.1	0.018
PV1MATH	Math achievement (PV1)	19.0	35.3	< 0.001
ICTRES	ICT resources	24.0	41.1	0.008
GROSAGR	Growth mindset	24.0	33.1	0.007
TEACHSUP	Math teacher support	26.3	37.0	0.017
MISCED	Mother education	27.8	38.3	0.010
HISEI	Parent ISEI	29.8	37.5	0.014
SCHRISK	School risk	32.3	43.7	0.016
MISSSC	Missing school	37.4	50.6	0.003
GRADE	Grade vs modal	40.6	52.1	0.003
SOCONPA	Social conn. parents	41.4	50.6	0.014
CURSUPP	Current support	41.9	56.6	< 0.001
PARINVOL	Parental involvement	42.2	54.3	< 0.001
ICTSCH	ICT availability school	43.4	51.0	0.005
CREATOPN	Creative openness	43.7	53.0	0.016
PASCHPOL	School parent policy	43.8	54.7	0.002
SKIPPING	Class skipping	44.2	53.3	< 0.001
ICTHOME	ICT availability home	46.3	53.4	0.003
ICTAVHOM	ICT use at home	46.5	55.1	< 0.001
ICTAVSCH	ICT use at school	47.0	53.2	0.009

9.2 S8b. Outcome: ANXMAT

Table S8: Exploratory low-IDV-aligned rank differences for ANXMAT in the initial unadjusted analysis: features whose mean rank is lower (more important) in the low-IDV than in the high-IDV cluster under a direction-only rule (BH-FDR-corrected permutation $p < 0.05$). These features do not satisfy the C-code inclusion criterion and are reported only as exploratory rank contrasts.

Code	Feature label	Mean rank low-IDV	Mean rank high-IDV	Perm. p
MATHPERS	Math persistence	4.6	12.5	< 0.001
EXPO21ST	Exposure 21c math	12.7	19.2	0.020
DISCLIM	Disciplinary climate	13.7	22.2	0.019
EXPOFA	Exposure formal math	16.2	23.1	0.010
PV1READ	Reading achievement (PV1)	19.7	30.6	0.003
CURIOAGR	Curiosity	23.5	31.5	0.010
FAMSUP	Family support	23.9	32.0	0.017
COGACRCO	Cognitive reasoning	24.0	31.6	0.018
SDLEFF	SDL self-efficacy	24.3	35.1	< 0.001
FEELLAH	Feelings learning home	30.3	42.9	0.002
HISEI	Parent ISEI	36.0	42.6	0.018
ICTRES	ICT resources	36.5	49.6	0.004
SCHRISK	School risk	39.2	47.6	0.006
AGE	Age	39.8	47.2	0.011
CURSUPP	Current support	41.9	56.2	< 0.001
SOCCON	Social connection	42.0	51.0	0.017
CREATOPN	Creative openness	43.3	54.0	< 0.001
PASCHPOL	School parent policy	43.5	53.8	0.002
PARINVOL	Parental involvement	44.2	53.5	0.002
ICTHOME	ICT availability home	44.3	52.7	0.005
ICTSCH	ICT availability school	44.8	50.9	0.011
MISSSC	Missing school	44.8	54.7	< 0.001
ICTAVHOM	ICT use at home	45.1	54.2	< 0.001
ICTAVSCH	ICT use at school	46.8	54.3	0.005

10 TABLE S9. MICE MULTIPLE-IMPUTATION SENSITIVITY FOR ANXMAT

Table S9: Multiple-imputation sensitivity (MICE, $m = 5$) for ANXMAT: per-economy pooled R^2 and top-5 features by mean |SHAP| pooled across imputations. Relative to the source-consistent native-missingness analysis, the MICE-pooled operational codes yield full Cohen's $\kappa = 0.927$ and U-coded-versus-not Cohen's $\kappa = 0.902$. This student-level missingness sensitivity does not address economy-level structural module non-availability.

Economy	n	R^2_{MICE}	Top 1	Top 2	Top 3	Top 4	Top 5
ALB	3,674	0.232	MATHEF21	MATHEFF	DISCLIM	FAMCON	PV1MATH
ARE	20,441	0.303	MATHEF21	MATHEFF	MATHPERS	PV1MATH	PSYCHSYM
ARG	7,857	0.143	MATHEFF	PV1MATH	MATHEF21	EMOCOAGR	MATHPERS
AUS	12,327	0.238	STRESAGR	MATHEFF	MATHEF21	MATHPERS	GROSAGR
AUT	5,228	0.217	MATHEFF	STRESAGR	MATHEF21	PV1MATH	PROBSELF
BEL	6,667	0.236	STRESAGR	MATHEF21	MATHEFF	TEACHSUP	LANGN
BGR	4,476	0.195	MATHPERS	MATHEF21	MATHEFF	IMAGINE	FAMCON
BRA	7,566	0.214	MATHEFF	STRESAGR	MATHEF21	MATHPERS	PV1MATH
BRN	4,822	0.168	STRESAGR	MATHEFF	MATHEF21	PROBSELF	EMPATAGR
CAN	18,834	0.244	MATHEFF	STRESAGR	MATHEF21	PV1MATH	MATHPERS
CHE	5,175	0.246	STRESAGR	MATHEFF	MATHEF21	PV1MATH	LANGN
CHL	5,372	0.214	MATHEFF	STRESAGR	MATHEF21	PV1MATH	ST004D01T
COL	6,342	0.143	ST004D01T	MATHEFF	MATHPERS	MATHEF21	CREATEFF
CRI	5,646	0.212	STRESAGR	MATHEFF	PV1MATH	ST004D01T	MATHEF21
CZE	7,567	0.299	MATHEFF	PV1MATH	MATHEF21	STRESAGR	GROSAGR
DEU	4,812	0.334	MATHEFF	STRESAGR	PV1MATH	MATHEF21	CREATEFF
DNK	4,967	0.342	MATHEFF	PV1MATH	STRESAGR	MATHEF21	ST004D01T
DOM	3,343	0.179	MATHPERS	MATHEF21	ST004D01T	EXPO21ST	EMOCOAGR
ESP	27,646	0.296	MATHEF21	MATHEFF	STRESAGR	PV1MATH	PSYCHSYM
EST	6,018	0.360	MATHEFF	STRESAGR	MATHEF21	PV1MATH	PROBSELF
FIN	8,450	0.285	MATHEFF	STRESAGR	MATHEF21	PV1MATH	PROBSELF
FRA	5,478	0.282	STRESAGR	MATHEFF	PV1MATH	MATHEF21	PROBSELF
GBR	10,072	0.283	STRESAGR	MATHEFF	MATHEF21	PV1MATH	ST004D01T
GEO	4,497	0.191	MATHPERS	MATHEFF	ST004D01T	EMOCOAGR	MATHEF21
GRC	5,770	0.293	MATHEFF	STRESAGR	MATHEF21	MATHPERS	PV1MATH
GTM	3,736	0.104	MATHEFF	EMOCOAGR	PV1MATH	PROBSELF	BULLIED
HKG	5,521	0.354	MATHEFF	MATHEF21	STRESAGR	PV1MATH	GROSAGR
HRV	5,579	0.256	MATHEF21	STRESAGR	MATHEFF	TEACHSUP	PV1MATH
HUN	5,465	0.292	MATHEFF	PV1MATH	STRESAGR	TEACHSUP	MATHEF21
IRL	5,278	0.375	STRESAGR	MATHEF21	MATHEFF	PSYCHSYM	PV1MATH
ISL	2,765	0.247	MATHEFF	STRESAGR	PV1MATH	MATHPERS	DISCLIM
ITA	9,749	0.194	STRESAGR	MATHEF21	PV1MATH	MATHEFF	TEACHSUP
JAM	2,322	0.151	MATHEFF	MATHEF21	MATHPERS	GROSAGR	EMOCOAGR
JOR	5,594	0.201	MATHPERS	MATHEFF	MATHEF21	COGACMCO	DISCLIM
JPN	5,680	0.280	MATHEF21	MATHEFF	PROBSELF	MATHPERS	PV1READ
KAZ	18,876	0.184	MATHEF21	EMOCOAGR	MATHPERS	PROBSELF	PV1SCIE
KHM	4,184	0.143	MATHEFF	DISCLIM	MATHPERS	EXPO21ST	BULLIED
KOR	6,317	0.235	MATHEFF	STRESAGR	MATHEF21	ICTOUT	GROSAGR
KSV	4,428	0.216	MATHEFF	MATHEF21	ST004D01T	MATHPERS	GROSAGR
LTU	6,563	0.247	MATHEFF	STRESAGR	PV1MATH	MATHEF21	ST004D01T
LVA	4,623	0.285	MATHEFF	STRESAGR	PV1MATH	MATHEF21	GROSAGR
MAC	4,314	0.308	MATHEFF	MATHEF21	STRESAGR	MATHPERS	PSYCHSYM
MAR	4,296	0.191	MATHPERS	MATHEFF	MATHEF21	BULLIED	IMAGINE
MDA	5,518	0.260	MATHEFF	PV1MATH	MATHEF21	GROSAGR	EMOCOAGR
MEX	5,545	0.128	MATHEF21	MATHEFF	PSYCHSYM	PV1MATH	GROSAGR

Economy	n	R^2_{MICE}	Top 1	Top 2	Top 3	Top 4	Top 5
MKD	4,868	0.237	MATHEFF	MATHPERS	MATHEF21	FAMCON	ST004D01T
MLT	2,679	0.313	MATHEF21	MATHEFF	STRESAGR	PV1MATH	EMOCOAGR
MNE	4,768	0.219	MATHEF21	MATHPERS	MATHEFF	PV1MATH	EMOCOAGR
MNG	6,262	0.295	MATHEFF	MATHPERS	MATHEF21	GROSAGR	COGACMCO
MYS	6,351	0.229	EMOCOAGR	MATHEFF	MATHEF21	GROSAGR	PV1READ
NLD	4,352	0.117	MATHEFF	MATHEF21	PSYCHSYM	STRESAGR	PROBSELF
NOR	5,466	0.342	MATHEFF	ST004D01T	MATHEF21	PV1MATH	TEACHSUP
NZL	4,016	0.243	MATHEFF	STRESAGR	MATHEF21	PSYCHSYM	PROBSELF
PAN	2,098	0.122	MATHEFF	ICTOUT	PSYCHSYM	MATHEF21	MATHPERS
PER	5,150	0.186	MATHEFF	ST004D01T	PV1MATH	MATHPERS	EMOCOAGR
POL	5,406	0.192	MATHEFF	MATHEF21	PV1MATH	STRESAGR	MATHPERS
PRT	5,913	0.287	MATHEFF	STRESAGR	MATHEF21	PV1MATH	MATHPERS
PRY	3,810	0.211	MATHEFF	EMOCOAGR	PV1MATH	MATHPERS	DISCLIM
PSE	6,162	0.174	MATHEFF	MATHEF21	MATHPERS	DISCLIM	PV1MATH
QAT	5,343	0.261	MATHEFF	MATHPERS	MATHEF21	PV1MATH	PV1READ
QAZ	3,059	0.118	MATHEF21	MATHPERS	MATHEFF	RELATST	PV1MATH
QUR	2,962	0.177	STRESAGR	MATHEF21	MATHEFF	COGACRCO	MATHPERS
ROU	6,356	0.154	STRESAGR	MATHPERS	PV1MATH	MATHEFF	RELATST
SAU	5,816	0.270	MATHEFF	MATHPERS	MATHEF21	GROSAGR	PV1MATH
SGP	6,524	0.242	STRESAGR	MATHEF21	MATHEFF	PV1MATH	FAMCON
SLV	4,678	0.091	MATHPERS	ST004D01T	MATHEF21	PV1MATH	EMOCOAGR
SRB	5,514	0.179	MATHEF21	MATHEFF	STRESAGR	MATHPERS	GROSAGR
SVK	5,038	0.258	MATHEF21	MATHEFF	STRESAGR	PV1MATH	EMOCOAGR
SVN	6,166	0.234	MATHEF21	MATHEFF	STRESAGR	PSYCHSYM	FAMCON
SWE	5,260	0.299	MATHEFF	PV1MATH	MATHEF21	ST004D01T	MATHPERS
TAP	5,747	0.250	MATHEF21	MATHEFF	STRESAGR	PV1MATH	GROSAGR
TUR	7,059	0.211	MATHEF21	MATHEFF	STRESAGR	MATHPERS	ST004D01T
URY	4,389	0.166	PV1MATH	MATHEFF	MATHEF21	STRESAGR	MATHPERS
USA	4,183	0.246	MATHEFF	MATHEF21	PV1MATH	BULLIED	ST004D01T
UZB	5,063	0.191	MATHPERS	MATHEFF	DISCLIM	PV1MATH	MATHEF21
VNM	5,934	0.238	MATHPERS	MATHEFF	EMOCOAGR	PROBSELF	DISCLIM

11 TABLE S10. PREDICTOR DICTIONARY FOR THE 73 CANDIDATE VARIABLES

Table S10: Predictor dictionary for the 73 candidate variables used in the per-economy LightGBM and TreeSHAP analyses. The description column uses PISA 2022 metadata labels when available. Economy code was not supplied to those per-economy models; it was included as a categorical predictor only in the pooled comparator described in the main text.

Code	Description	Block	Source type	Coding note	Categorical
ST004D01T	Student (Standardized) Gender	Demographics	categorical item/index	Treated as categorical in LightGBM.	yes
IMMIG	Index on immigrant background (OECD definition)	Demographics	categorical item/index	Treated as categorical in LightGBM.	yes
GRADE	Grade compared to modal grade in country	Demographics	categorical item/index	Treated as categorical in LightGBM.	yes
MISCED	Mother's level of education (ISCED)	Demographics	categorical item/index	Treated as categorical in LightGBM.	yes
FISCED	Father's level of education (ISCED)	Demographics	categorical item/index	Treated as categorical in LightGBM.	yes
LANGN	Language spoken at home	Demographics	categorical item/index	Treated as categorical in LightGBM.	yes
AGE	Students' age	Demographics	derived continuous variable	Numeric predictor used as released by OECD.	no
ESCS	Index of economic, social and cultural status	Socioeconomic resources	OECD index	Numeric predictor used as released by OECD.	no
HISEI	Highest parental occupational status (ISEI) based on 4-digit human coded ISCO	Socioeconomic resources	derived continuous variable	Numeric predictor used as released by OECD.	no
HOMEPOS	Home possessions (WLE)	Socioeconomic resources	OECD WLE index	OECD weighted-likelihood-estimate scale.	no
ICTRES	ICT Resources (WLE)	Socioeconomic resources	OECD WLE index	OECD weighted-likelihood-estimate scale.	no
ICTHOME	ICT availability outside of school (WLE)	Socioeconomic resources	OECD WLE index	OECD weighted-likelihood-estimate scale.	no
BMMJ1	Mother's occupational status (ISEI) based on 4-digit human coded ISCO	Socioeconomic resources	derived continuous variable	Numeric predictor used as released by OECD.	no
BFMJ2	Father's occupational status (ISEI) based on 4-digit human coded ISCO	Socioeconomic resources	derived continuous variable	Numeric predictor used as released by OECD.	no
PV1MATH	Plausible Value 1 in Mathematics	Achievement	plausible value	PV1 used in primary analysis; PV1–PV10 checked in sensitivity analysis.	no
PV1READ	Plausible Value 1 in Reading	Achievement	plausible value	PV1 used in primary analysis; PV1–PV10 checked in sensitivity analysis.	no

continued on next page

Code	Description	Block	Source type	Coding note	Categorical
PV1SCIE	Plausible Value 1 in Science	Achievement	plausible value	PV1 used in primary analysis; PV1–PV10 checked in sensitivity analysis.	no
MATHEFF	Mathematics self-efficacy: formal and applied mathematics - response options reversed in 2022 (WLE)	Affective/motivational	OECD WLE index	OECD weighted-likelihood-estimate scale.	no
MATHEF21	Mathematics self-efficacy: mathematical reasoning and 21st century skills (WLE)	Affective/motivational	OECD WLE index	OECD weighted-likelihood-estimate scale.	no
ASSERAGR	Assertiveness (agreement) (WLE)	Affective/motivational	OECD WLE index	OECD weighted-likelihood-estimate scale.	no
COOPAGR	Cooperation (agreement) (WLE)	Affective/motivational	OECD WLE index	OECD weighted-likelihood-estimate scale.	no
EMPATAGR	Empathy (agreement) (WLE)	Affective/motivational	OECD WLE index	OECD weighted-likelihood-estimate scale.	no
PERSEVAGR	Perseverance (agreement) (WLE)	Affective/motivational	OECD WLE index	OECD weighted-likelihood-estimate scale.	no
STRESAGR	Stress resistance (agreement) (WLE)	Affective/motivational	OECD WLE index	OECD weighted-likelihood-estimate scale.	no
EMOCOAGR	Emotional control (agreement) (WLE)	Affective/motivational	OECD WLE index	OECD weighted-likelihood-estimate scale.	no
CURIOAGR	Curiosity (agreement) (WLE)	Affective/motivational	OECD WLE index	OECD weighted-likelihood-estimate scale.	no
GROSAGR	Growth Mindset (WLE)	Growth mindset	OECD WLE index	OECD weighted-likelihood-estimate scale.	no
EXPWB	Experienced Well-being (Previous Day) (WLE)	Wellbeing/experience	OECD WLE index	OECD weighted-likelihood-estimate scale.	no
LIFESAT	Students' Life Satisfaction across Domains (WLE)	Wellbeing/experience	OECD WLE index	OECD weighted-likelihood-estimate scale.	no
PSYCHSYM	Psychosomatic Symptoms (WLE)	Wellbeing/experience	OECD WLE index	OECD weighted-likelihood-estimate scale.	no
BODYIMA	Body Image (WLE)	Wellbeing/experience	OECD WLE index	OECD weighted-likelihood-estimate scale.	no
FEELLAH	Feelings about learning at home (WLE)	Wellbeing/experience	OECD WLE index	OECD weighted-likelihood-estimate scale.	no
DISCLIM	Disciplinary climate in mathematics (WLE)	School climate/safety	OECD WLE index	OECD weighted-likelihood-estimate scale.	no
BULLIED	Exposure to bullying (WLE)	School climate/safety	OECD WLE index	Higher WLE denotes more frequent exposure to bullying.	no

continued on next page

Code	Description	Block	Source type	Coding note	Categorical
RELATST	Quality of student-teacher relationships (WLE)	School climate/safety	OECD WLE index	OECD weighted-likelihood-estimate scale.	no
FEELSAFE	Feeling safe (WLE)	School climate/safety	OECD WLE index	OECD weighted-likelihood-estimate scale.	no
SCHRISK	School safety risks (WLE)	School climate/safety	OECD WLE index	OECD weighted-likelihood-estimate scale.	no
TEACHSUP	Mathematics Teacher Support (WLE)	Pedagogy	OECD WLE index	OECD weighted-likelihood-estimate scale.	no
COGACMCO	Cognitive activation in mathematics: Encourage mathematical thinking Version B (WLE)	Pedagogy	OECD WLE index	OECD weighted-likelihood-estimate scale.	no
COGACRCO	Cognitive activation in mathematics: Foster reasoning Version B (WLE)	Pedagogy	OECD WLE index	OECD weighted-likelihood-estimate scale.	no
MATHPERS	Effort and Persistence in Mathematics (WLE)	Pedagogy	OECD WLE index	OECD weighted-likelihood-estimate scale.	no
EXPO21ST	Exposure to Mathematical Reasoning and 21st century mathematics tasks (WLE)	Pedagogy	OECD WLE index	OECD weighted-likelihood-estimate scale.	no
EXPOFA	Exposure to Formal and Applied Mathematics Tasks (WLE)	Pedagogy	OECD WLE index	OECD weighted-likelihood-estimate scale.	no
FAMCON	Subjective familiarity with mathematics concepts (WLE)	Pedagogy	OECD WLE index	OECD weighted-likelihood-estimate scale.	no
SKIPPING	Skipping classes or days of school	Engagement	continuous item/index	Numeric predictor used as released by OECD.	no
MISSSC	Missing school for more than 3 months	Engagement	continuous item/index	Numeric predictor used as released by OECD.	no
ICTAVHOM	Availability and Usage of ICT at Home	ICT	continuous item/index	Numeric predictor used as released by OECD.	no
ICTAVSCH	Availability and Usage of ICT at School	ICT	continuous item/index	Numeric predictor used as released by OECD.	no
ICTOUT	Use of ICT for school activities outside of the classroom (WLE)	ICT	OECD WLE index	OECD weighted-likelihood-estimate scale.	no
ICTSCH	ICT availability at school (WLE)	ICT	OECD WLE index	OECD weighted-likelihood-estimate scale.	no
ICTQUAL	Quality of access to ICT (WLE)	ICT	OECD WLE index	OECD weighted-likelihood-estimate scale.	no
ICTSUBJ	Subject-related ICT Use During Lessons (WLE)	ICT	OECD WLE index	OECD weighted-likelihood-estimate scale.	no

continued on next page

Code	Description	Block	Source type	Coding note	Categorical
ICTWKDY	Frequency of ICT activity (weekday) (WLE)	ICT	OECD WLE index	OECD weighted-likelihood-estimate scale.	no
ICTWKEND	Frequency of ICT activity (weekend) (WLE)	ICT	OECD WLE index	OECD weighted-likelihood-estimate scale.	no
ICTEFFIC	Self-efficacy in digital competencies (WLE)	ICT	OECD WLE index	OECD weighted-likelihood-estimate scale.	no
ICTENQ	Use of ICT in enquiry-based learning activities (WLE)	ICT	OECD WLE index	OECD weighted-likelihood-estimate scale.	no
ICTFEED	Support or feedback via ICT (WLE)	ICT	OECD WLE index	OECD weighted-likelihood-estimate scale.	no
ICTINFO	Students' practices regarding online information (WLE)	ICT	OECD WLE index	OECD weighted-likelihood-estimate scale.	no
ICTREG	Views of regulated ICT use in school (WLE)	ICT	OECD WLE index	OECD weighted-likelihood-estimate scale.	no
FAMSUP	Family support (WLE)	Family/parental	OECD WLE index	OECD weighted-likelihood-estimate scale.	no
FAMSUPSL	Family support for self-directed learning (WLE)	Family/parental	OECD WLE index	OECD weighted-likelihood-estimate scale.	no
CURSUPP	Current Parental/guardian Support (WLE)	Family/parental	OECD WLE index	OECD weighted-likelihood-estimate scale.	no
PARINVOL	Parental Involvement (WLE)	Family/parental	OECD WLE index	OECD weighted-likelihood-estimate scale.	no
SOCONPA	Social connection to Parents (WLE)	Family/parental	OECD WLE index	OECD weighted-likelihood-estimate scale.	no
SOCCON	Social Connections: Ease of Communication About Worries and Concerns (WLE)	Family/parental	OECD WLE index	OECD weighted-likelihood-estimate scale.	no
PASCHPOL	School Policies for Parental Involvement (WLE)	Family/parental	OECD WLE index	OECD weighted-likelihood-estimate scale.	no
SDLEFF	Self-directed learning self-efficacy (WLE)	Self-regulation	OECD WLE index	OECD weighted-likelihood-estimate scale.	no
PROBSELF	Problems with self-directed learning (WLE)	Self-regulation	OECD WLE index	OECD weighted-likelihood-estimate scale.	no
INFOSEEK	Information seeking about future careers (WLE)	Self-regulation	OECD WLE index	OECD weighted-likelihood-estimate scale.	no
CREATEFF	Creative self-efficacy (WLE)	Creativity	OECD WLE index	OECD weighted-likelihood-estimate scale.	no

continued on next page

Code	Description	Block	Source type	Coding note	Categorical
CREATOPN	Creativity and Openness to Intellect (WLE)	Creativity	OECD WLE index	OECD weighted-likelihood-estimate scale.	no
IMAGINE	Imagination and Adventurousness (WLE)	Creativity	OECD WLE index	OECD weighted-likelihood-estimate scale.	no
OPENART	Openness to Art and Reflection (WLE)	Creativity	OECD WLE index	OECD weighted-likelihood-estimate scale.	no

12 TABLE S11. OFFICIAL 2023 IDV SCORES AND TERCILE MEMBERSHIP

Table S11: Official 2023 individualism (IDV) scores and tercile membership for 74 economies: 25 low-, 25 mid-, and 24 high-IDV economies. BELONG and ANXMAT indicate inclusion in the initial outcome-specific per-economy LightGBM analysis. Six PISA economies with outcome data have no official IDV score and are therefore absent from this table and all IDV-based analyses: BRN, KHM, KSV, MAC, PSE, and UZB. QAZ and QUR use Azerbaijan and Ukraine country scores, respectively; TAP uses the direct Taiwan score.

Economy	Official source entry	IDV	IDV tercile	BELONG	ANXMAT	Mapping note
ALB	Albania	27	low IDV	yes	yes	Direct official country match
ARE	United Arab Emirates	36	low IDV	yes	yes	Direct official country match
BRA	Brazil	36	low IDV	yes	yes	Direct official country match
COL	Colombia	29	low IDV	yes	yes	Direct official country match
CRI	Costa Rica	15	low IDV	yes	yes	Direct official country match
GEO	Georgia	15	low IDV	yes	yes	Direct official country match
GTM	Guatemala	36	low IDV	yes	yes	Direct official country match
IDN	Indonesia	5	low IDV	yes	no	Direct official country match
JOR	Jordan	20	low IDV	yes	yes	Direct official country match
KAZ	Kazakhstan	20	low IDV	yes	yes	Direct official country match
MAR	Morocco	24	low IDV	yes	yes	Direct official country match
MDA	Moldova	27	low IDV	yes	yes	Direct official country match
MEX	Mexico	34	low IDV	yes	yes	Direct official country match
MNE	Montenegro	27	low IDV	yes	yes	Direct official country match
MNG	Mongolia	37	low IDV	yes	yes	Direct official country match
MYS	Malaysia	27	low IDV	yes	yes	Direct official country match
PAN	Panama	11	low IDV	yes	yes	Direct official country match
PER	Peru	20	low IDV	yes	yes	Direct official country match
PHL	Philippines	17	low IDV	yes	no	Direct official country match
PRY	Paraguay	12	low IDV	yes	yes	Direct official country match
QAT	Qatar	18	low IDV	yes	yes	Direct official country match
QAZ	Azerbaijan	28	low IDV	yes	yes	Azerbaijan country score; omitted in the subnational sensitivity
SLV	El Salvador	19	low IDV	yes	yes	Direct official country match
THA	Thailand	19	low IDV	yes	no	Direct official country match
VNM	Vietnam	30	low IDV	yes	yes	Direct official country match
ARG	Argentina	51	mid IDV	yes	yes	Direct official country match
BGR	Bulgaria	50	mid IDV	yes	yes	Direct official country match
CHL	Chile	49	mid IDV	yes	yes	Direct official country match
DOM	Dominican Republic	38	mid IDV	yes	yes	Direct official country match
GRC	Greece	59	mid IDV	yes	yes	Direct official country match
HKG	Hong Kong	50	mid IDV	yes	yes	Direct official country match
HRV	Croatia	42	mid IDV	yes	yes	Direct official country match
IRL	Ireland	58	mid IDV	yes	yes	Direct official country match
ISR	Israel	56	mid IDV	no	no	Direct official country match
ITA	Italy	53	mid IDV	yes	yes	Direct official country match
JAM	Jamaica	39	mid IDV	yes	yes	Direct official country match
KOR	South Korea	58	mid IDV	yes	yes	Direct official country match
LTU	Lithuania	55	mid IDV	yes	yes	Direct official country match
MKD	North Macedonia	40	mid IDV	yes	yes	Direct official country match
MLT	Malta	59	mid IDV	yes	yes	Direct official country match
POL	Poland	47	mid IDV	yes	yes	Direct official country match

continued on next page

Economy	Official source entry	IDV	IDV tercile	BELONG	ANXMAT	Mapping note
PRT	Portugal	59	mid IDV	yes	yes	Direct official country match
QUR	Ukraine	55	mid IDV	yes	yes	Ukraine country score; omitted in the subnational sensitivity
ROU	Romania	46	mid IDV	yes	yes	Direct official country match
SAU	Saudi Arabia	48	mid IDV	yes	yes	Direct official country match
SGP	Singapore	43	mid IDV	yes	yes	Direct official country match
SRB	Serbia	42	mid IDV	yes	yes	Direct official country match
SVK	Slovakia	57	mid IDV	yes	yes	Direct official country match
TAP	Taiwan	40	mid IDV	yes	yes	Direct Taiwan score; retained in the subnational sensitivity
TUR	Turkey	46	mid IDV	yes	yes	Direct official country match
AUS	Australia	73	high IDV	yes	yes	Direct official country match
AUT	Austria	77	high IDV	yes	yes	Direct official country match
BEL	Belgium	81	high IDV	yes	yes	Direct official country match
CAN	Canada	72	high IDV	yes	yes	Direct official country match
CHE	Switzerland	79	high IDV	yes	yes	Direct official country match
CZE	Czech Republic	70	high IDV	yes	yes	Direct official country match
DEU	Germany	79	high IDV	yes	yes	Direct official country match
DNK	Denmark	89	high IDV	yes	yes	Direct official country match
ESP	Spain	67	high IDV	yes	yes	Direct official country match
EST	Estonia	62	high IDV	yes	yes	Direct official country match
FIN	Finland	75	high IDV	yes	yes	Direct official country match
FRA	France	74	high IDV	yes	yes	Direct official country match
GBR	United Kingdom	76	high IDV	yes	yes	Direct official country match
HUN	Hungary	71	high IDV	yes	yes	Direct official country match
ISL	Iceland	83	high IDV	yes	yes	Direct official country match
JPN	Japan	62	high IDV	yes	yes	Direct official country match
LVA	Latvia	70	high IDV	yes	yes	Direct official country match
NLD	Netherlands	100	high IDV	yes	yes	Direct official country match
NOR	Norway	81	high IDV	yes	yes	Direct official country match
NZL	New Zealand	69	high IDV	yes	yes	Direct official country match
SVN	Slovenia	81	high IDV	yes	yes	Direct official country match
SWE	Sweden	87	high IDV	yes	yes	Direct official country match
URY	Uruguay	60	high IDV	yes	yes	Direct official country match
USA	United States	60	high IDV	yes	yes	Direct official country match

13 TABLE S12. WEIGHTED VERSUS UNWEIGHTED SHAP SENSITIVITY

Table S12: Weighted versus unweighted SHAP sensitivity for the initial unadjusted analysis. Weighted models used the PISA final student weight in LightGBM fitting and weighted mean absolute SHAP aggregation. No focal feature changed code in either outcome.

Outcome	Economies	Features	Economy rho	Top-5 overlap	Cluster rho L/M/H	Full kappa	U kappa	W kappa	Changed features	Focal changes
BELONG	78	73	0.953	4.78	0.992/0.987/0.982	0.918	1.000	0.793	OPENART:W to I	none
ANXMAT	76	73	0.962	4.47	0.990/0.990/0.994	0.917	1.000	0.660	TEACHSUP:W to I	none

14 TABLE S13. RANDOM-SEED STABILITY

Table S13: Random-seed stability of the initial unadjusted analysis for seeds 42, 202, 2022, 2024, and 777. All focal features retained their classifications across all seeds; observed changes were limited to non-focal or boundary features.

Outcome	Seed	Economy rho	Top-5 overlap	Full kappa	U kappa	W kappa	Focal retained	Max SD/IQR	Changed features
ANXMAT	42	0.975	4.57	1.000	1.000	1.000	6/6	0.870/1.273	none
ANXMAT	202	0.937	4.26	1.000	1.000	1.000	6/6	0.870/1.273	none
ANXMAT	777	0.933	4.24	0.928	1.000	0.793	6/6	0.870/1.273	FAMCON:I to W
ANXMAT	2022	0.932	4.26	0.928	1.000	0.793	6/6	0.870/1.273	FAMCON:I to W
ANXMAT	2024	0.939	4.24	1.000	1.000	1.000	6/6	0.870/1.273	none
BELONG	42	0.964	4.87	1.000	1.000	1.000	6/6	0.536/0.625	none
BELONG	202	0.920	4.47	0.918	1.000	0.793	6/6	0.536/0.625	OPENART:W to I
BELONG	777	0.914	4.50	0.918	1.000	0.793	6/6	0.536/0.625	OPENART:W to I
BELONG	2022	0.909	4.50	1.000	1.000	1.000	6/6	0.536/0.625	none
BELONG	2024	0.923	4.50	0.918	1.000	0.793	6/6	0.536/0.625	OPENART:W to I

15 TABLE S14. PLAUSIBLE-VALUES SENSITIVITY

Table S14: Plausible-values sensitivity for the initial unadjusted analysis. Each run replaced PV1MATH, PV1READ, and PV1SCIE with the corresponding plausible value set. Mathematics achievement retained the operational U code for ANXMAT and I code for BELONG across all ten plausible-value specifications.

Outcome	PV	Achievement feature	Code	Rank L/M/H	Full kappa	U kappa	Focal retained	Changed features
ANXMAT	1	PVMATH	U	6.864/6.667/4.250	1.000	1.000	6/6	none
ANXMAT	2	PVMATH	U	6.273/7.125/4.500	1.000	1.000	6/6	none
ANXMAT	3	PVMATH	U	6.864/6.083/3.875	1.000	1.000	6/6	none
ANXMAT	4	PVMATH	U	7.091/6.000/4.333	1.000	1.000	6/6	none
ANXMAT	5	PVMATH	U	7.409/6.292/4.375	1.000	1.000	6/6	none
ANXMAT	6	PVMATH	U	6.591/6.542/4.250	1.000	1.000	6/6	none
ANXMAT	7	PVMATH	U	7.864/6.083/4.250	1.000	1.000	6/6	none
ANXMAT	8	PVMATH	U	6.636/6.292/4.292	1.000	1.000	6/6	none
ANXMAT	9	PVMATH	U	6.136/6.042/4.000	1.000	1.000	6/6	none
ANXMAT	10	PVMATH	U	7.591/6.167/3.875	0.927	0.902	6/6	PROBSELF:I to U
BELONG	1	PVMATH	I	19.960/35.458/32.917	1.000	1.000	6/6	none
BELONG	2	PVMATH	I	21.240/32.250/36.667	0.918	1.000	6/6	OPENART:W to I
BELONG	3	PVMATH	I	22.200/31.708/34.583	0.918	1.000	6/6	OPENART:W to I
BELONG	4	PVMATH	I	20.080/31.792/35.375	0.918	1.000	6/6	OPENART:W to I
BELONG	5	PVMATH	I	18.840/33.208/33.500	0.918	1.000	6/6	OPENART:W to I
BELONG	6	PVMATH	I	22.720/36.042/35.000	0.918	1.000	6/6	OPENART:W to I
BELONG	7	PVMATH	I	22.680/35.000/32.292	0.918	1.000	6/6	OPENART:W to I
BELONG	8	PVMATH	I	20.280/32.292/36.292	0.918	1.000	6/6	OPENART:W to I
BELONG	9	PVMATH	I	19.960/34.542/37.750	1.000	1.000	6/6	none
BELONG	10	PVMATH	I	18.840/35.125/36.083	0.918	1.000	6/6	OPENART:W to I

16 TABLE S15. PERCENTILE-THRESHOLD SENSITIVITY

Table S15: Percentile-threshold sensitivity for the initial unadjusted analysis. Rank thresholds were converted to percentile importance thresholds mathematically equivalent to the initial rank cutoffs. The percentile-normalised classification exactly reproduced the initial classification for both outcomes.

**Outcome**	**Primary U/W/C**	**Percentile U/W/C**	**Full kappa**	**U kappa**	**W kappa**	**Changed features**	**Focal changes**
BELONG	3/3/1	3/3/1	1.000	1.000	1.000	none	none
ANXMAT	5/2/0	5/2/0	1.000	1.000	1.000	none	none

17 TABLE S16. MEAN VERSUS MEDIAN AGGREGATION COMPARISON

Table S16: Mean-versus-median aggregation comparison for the initial unadjusted analysis. No focal code changed, although several non-focal boundary features changed code. The comparison shows that exact all-feature assignments depend partly on the aggregation statistic.

Outcome	Full kappa	U kappa	W kappa	Changed features	Focal changes
BELONG	0.808	0.850	0.736	EMOCOAGR:I to U;ICTREG:I to W;PERSEVAGR:I to W	none
ANXMAT	0.863	0.819	1.000	EMOCOAGR:I to U;PROBSELF:I to U	none

Table S16: Changed features in the mean-versus-median comparison for the initial unadjusted analysis, with within-cluster rank-distribution summaries. The distribution notes show counts in top-5, ranks 6–15, ranks 16–30, and ranks 31+ within each IDV cluster.

Outcome	Feature	Shift	Mean L/M/H	Median L/M/H	Rank-distribution note
BELONG	EMOCOAGR	I to U	19.240/13.625/23.083	10.000/11.500/14.000	L: top5=5, 6–15=11, 16–30=2, 31+=7 ; M: top5=2, 6–15=14, 16–30=7, 31+=1 ; H: top5=0, 6–15=13, 16–30=3, 31+=8
BELONG	ICTREG	I to W	34.480/32.292/23.208	39.000/34.000/15.000	L: top5=0, 6–15=3, 16–30=5, 31+=17 ; M: top5=0, 6–15=5, 16–30=6, 31+=13 ; H: top5=0, 6–15=12, 16–30=5, 31+=7
BELONG	PERSEVAGR	I to W	36.240/18.917/19.500	41.000/12.000/12.500	L: top5=3, 6–15=4, 16–30=0, 31+=18 ; M: top5=1, 6–15=14, 16–30=4, 31+=5 ; H: top5=1, 6–15=13, 16–30=3, 31+=7
ANXMAT	EMOCOAGR	I to U	16.318/10.333/20.417	6.000/9.000/10.500	L: top5=10, 6–15=6, 16–30=0, 31+=6 ; M: top5=2, 6–15=20, 16–30=1, 31+=1 ; H: top5=3, 6–15=13, 16–30=2, 31+=6
ANXMAT	PROBSELF	I to U	16.727/14.292/13.292	14.500/8.500/8.500	L: top5=2, 6–15=10, 16–30=9, 31+=1 ; M: top5=0, 6–15=17, 16–30=4, 31+=3 ; H: top5=3, 6–15=15, 16–30=3, 31+=3

Table S17. Economy–feature availability assessment and percentile summary for the twelve focal outcome–predictor combinations. Coverage and mean percentile importance are reported for low-/mid-/high-IDV terciles. A combination meets the coverage criterion only when its predictor is usable in at least 50% of outcome-eligible economies in every tercile; otherwise, it is classified as coverage-limited.

Outcome	Predictor	Coverage L/M/H	Percentile L/M/H	Availability status
ANXMAT	GROSAGR	86%/100%/92%	0.84/0.86/0.84	coverage criterion met
ANXMAT	MATHEF21	86%/100%/100%	0.97/0.97/0.97	coverage criterion met
ANXMAT	MATHEFF	91%/100%/100%	0.98/0.98/0.99	coverage criterion met
ANXMAT	MATHPERS	95%/100%/100%	0.95/0.92/0.81	coverage criterion met
ANXMAT	PV1MATH	100%/100%/100%	0.88/0.90/0.94	coverage criterion met
ANXMAT	STRESAGR	9%/75%/83%	0.99/0.98/0.98	coverage-limited
BELONG	ASSERAGR	12%/83%/83%	0.92/0.93/0.92	coverage-limited
BELONG	BULLIED	100%/100%/100%	0.95/0.96/0.98	coverage criterion met
BELONG	COOPAGR	20%/63%/75%	0.95/0.98/0.96	coverage-limited
BELONG	FEELSAFE	96%/100%/83%	0.99/0.99/1.00	coverage criterion met
BELONG	RELATST	84%/100%/96%	0.97/0.96/0.94	coverage criterion met
BELONG	STRESAGR	8%/75%/83%	0.81/0.92/0.92	coverage-limited

Table S17 separates predictive salience from questionnaire availability. Four social-emotional combinations selected for cultural follow-up fail the stated availability criterion because fewer than half of outcome-eligible economies in at least one IDV tercile provide usable scores. They are therefore retained only as coverage-limited exploratory results. The other eight focal combinations meet the availability criterion. Their stable relative predictive salience is based on the rank and bootstrap criteria reported in the main text and machine-readable results; it does not imply context-invariant psychological mechanisms.

Documentation and missingness check. Student item nonresponse, planned item-level missingness from the balanced incomplete block design, and economy-level structural module non-availability were treated as distinct conditions. Native LightGBM missing-value routing applies only within an implemented predictor, and the MICE sensitivity analysis addresses student-level missingness; neither method was used to create values for an economy in which a questionnaire module was structurally unavailable. The economy–feature availability assessment therefore precedes the coverage-adjusted comparisons, and each variable is compared only among economies meeting the stated availability rule.

Table S18. Complete focal main-association and IDV-interaction estimates for the economy fixed-effects primary models and multilevel sensitivity models. Coefficients are standardised associations, not causal effects. Main-association and interaction q values were BH-FDR-corrected in separate six-test families within each outcome and model type.

S18a. Economy fixed-effects models

Outcome	Predictor	Term	β [95% CI]	p	q	N / G	Status
ANXMAT	GROSAGR	Main	-0.0797 [-0.0928, -0.0666]	$p < .001$	$q < .001$	374,425 / 60	stable-rank combination
ANXMAT	GROSAGR	× IDV	-0.0278 [-0.0444, -0.0111]	$p = 0.001$	$q = 0.003$	374,425 / 60	stable-rank combination
ANXMAT	MATHEF21	Main	-0.3224 [-0.3374, -0.3074]	$p < .001$	$q < .001$	377,227 / 61	stable-rank combination
ANXMAT	MATHEF21	× IDV	-0.0076 [-0.0217, 0.0064]	$p = 0.282$	$q = 0.282$	377,227 / 61	stable-rank combination
ANXMAT	MATHEFF	Main	-0.3404 [-0.3555, -0.3253]	$p < .001$	$q < .001$	369,638 / 62	stable-rank combination
ANXMAT	MATHEFF	× IDV	-0.0126 [-0.0286, 0.0034]	$p = 0.121$	$q = 0.146$	369,638 / 62	stable-rank combination
ANXMAT	MATHPERS	Main	-0.2361 [-0.2557, -0.2165]	$p < .001$	$q < .001$	391,107 / 63	stable-rank combination
ANXMAT	MATHPERS	× IDV	0.0465 [0.0263, 0.0667]	$p < .001$	$q < .001$	391,107 / 63	stable-rank combination
ANXMAT	PVMATH	Main	-0.2331 [-0.2447, -0.2214]	$p < .001$	$q < .001$	401,045 / 64	stable-rank combination
ANXMAT	PVMATH	× IDV	-0.0328 [-0.0497, -0.0159]	$p < .001$	$q < .001$	401,045 / 64	stable-rank combination
ANXMAT	STRESAGR	Main	-0.2458 [-0.2646, -0.2270]	$p < .001$	$q < .001$	241,786 / 37	coverage limited
ANXMAT	STRESAGR	× IDV	-0.0310 [-0.0519, -0.0101]	$p = 0.005$	$q = 0.007$	241,786 / 37	coverage limited
BELONG	ASSERAGR	Main	0.1814 [0.1566, 0.2061]	$p < .001$	$q < .001$	274,137 / 40	coverage limited
BELONG	ASSERAGR	× IDV	0.0007 [-0.0237, 0.0250]	$p = 0.956$	$q = 0.956$	274,137 / 40	coverage limited
BELONG	BULLIED	Main	-0.2534 [-0.2660, -0.2408]	$p < .001$	$q < .001$	476,803 / 67	stable-rank combination
BELONG	BULLIED	× IDV	-0.0306 [-0.0431, -0.0180]	$p < .001$	$q < .001$	476,803 / 67	stable-rank combination
BELONG	COOPAGR	Main	0.3045 [0.2800, 0.3291]	$p < .001$	$q < .001$	246,041 / 34	coverage limited
BELONG	COOPAGR	× IDV	0.0056 [-0.0144, 0.0256]	$p = 0.571$	$q = 0.685$	246,041 / 34	coverage limited
BELONG	FEELSAFE	Main	0.3234 [0.3142, 0.3326]	$p < .001$	$q < .001$	432,443 / 63	stable-rank combination
BELONG	FEELSAFE	× IDV	0.0248 [0.0153, 0.0342]	$p < .001$	$q < .001$	432,443 / 63	stable-rank combination
BELONG	RELATST	Main	0.2817 [0.2643, 0.2992]	$p < .001$	$q < .001$	427,031 / 62	stable-rank combination
BELONG	RELATST	× IDV	-0.0190 [-0.0335, -0.0045]	$p = 0.011$	$q = 0.017$	427,031 / 62	stable-rank combination
BELONG	STRESAGR	Main	0.2002 [0.1719, 0.2286]	$p < .001$	$q < .001$	258,011 / 37	coverage limited
BELONG	STRESAGR	× IDV	0.0422 [0.0109, 0.0736]	$p = 0.010$	$q = 0.017$	258,011 / 37	coverage limited

S18b. Multilevel sensitivity models

Outcome	Predictor	Term	β [95% CI]	p	q	N / G	Status
ANXMAT	GROSAGR	Main	-0.0826 [-0.0858, -0.0794]	$p < .001$	$q < .001$	374,425 / 60	stable-rank combination; RI
ANXMAT	GROSAGR	× IDV	-0.0263 [-0.0298, -0.0228]	$p < .001$	$q < .001$	374,425 / 60	stable-rank combination; RI

Outcome	Predictor	Term	β [95% CI]	p	q	N / G	Status
ANXMAT	MATHEF21	Main	-0.3216 [-0.3246, -0.3185]	$p < .001$	$q < .001$	377,227 / 61	stable-rank combination; RI
ANXMAT	MATHEF21	× IDV	-0.0056 [-0.0089, -0.0023]	$p < .001$	$q = 0.001$	377,227 / 61	stable-rank combination; RI
ANXMAT	MATHEFF	Main	-0.3402 [-0.3433, -0.3371]	$p < .001$	$q < .001$	369,638 / 62	stable-rank combination; RI
ANXMAT	MATHEFF	× IDV	-0.0112 [-0.0146, -0.0077]	$p < .001$	$q < .001$	369,638 / 62	stable-rank combination; RI
ANXMAT	MATHPERS	Main	-0.2393 [-0.2538, -0.2248]	$p < .001$	$q < .001$	391,107 / 63	stable-rank combination; RS
ANXMAT	MATHPERS	× IDV	0.0400 [0.0244, 0.0556]	$p < .001$	$q < .001$	391,107 / 63	stable-rank combination; RS
ANXMAT	PVMATH	Main	-0.2312 [-0.2447, -0.2178]	$p < .001$	$q < .001$	401,045 / 64	stable-rank combination; RS
ANXMAT	PVMATH	× IDV	-0.0347 [-0.0492, -0.0202]	$p < .001$	$q < .001$	401,045 / 64	stable-rank combination; RS
ANXMAT	STRESAGR	Main	-0.2457 [-0.2627, -0.2288]	$p < .001$	$q < .001$	241,786 / 37	coverage limited; RS
ANXMAT	STRESAGR	× IDV	-0.0278 [-0.0469, -0.0086]	$p = 0.005$	$q = 0.005$	241,786 / 37	coverage limited; RS
BELONG	ASSERAGR	Main	0.1885 [0.1709, 0.2060]	$p < .001$	$q < .001$	274,137 / 40	coverage limited; RS
BELONG	ASSERAGR	× IDV	-0.0008 [-0.0211, 0.0195]	$p = 0.938$	$q = 0.938$	274,137 / 40	coverage limited; RS
BELONG	BULLIED	Main	-0.2554 [-0.2582, -0.2527]	$p < .001$	$q < .001$	476,803 / 67	stable-rank combination; RI
BELONG	BULLIED	× IDV	-0.0295 [-0.0323, -0.0267]	$p < .001$	$q < .001$	476,803 / 67	stable-rank combination; RI
BELONG	COOPAGR	Main	0.3106 [0.2930, 0.3282]	$p < .001$	$q < .001$	246,041 / 34	coverage limited; RS
BELONG	COOPAGR	× IDV	0.0081 [-0.0114, 0.0275]	$p = 0.416$	$q = 0.499$	246,041 / 34	coverage limited; RS
BELONG	FEELSAFE	Main	0.3243 [0.3214, 0.3271]	$p < .001$	$q < .001$	432,443 / 63	stable-rank combination; RI
BELONG	FEELSAFE	× IDV	0.0256 [0.0226, 0.0285]	$p < .001$	$q < .001$	432,443 / 63	stable-rank combination; RI
BELONG	RELATST	Main	0.2873 [0.2736, 0.3010]	$p < .001$	$q < .001$	427,031 / 62	stable-rank combination; RS
BELONG	RELATST	× IDV	-0.0097 [-0.0245, 0.0050]	$p = 0.196$	$q = 0.295$	427,031 / 62	stable-rank combination; RS
BELONG	STRESAGR	Main	0.2033 [0.1978, 0.2088]	$p < .001$	$q < .001$	258,011 / 37	coverage limited; RI
BELONG	STRESAGR	× IDV	0.0437 [0.0377, 0.0498]	$p < .001$	$q < .001$	258,011 / 37	coverage limited; RI

Notes. G is the number of economies; RS = random intercept plus focal random slope; RI = random intercept only. The PVMATH estimates pool all ten mathematics plausible values. Any singular or non-converged RS model was refitted using the documented RI fallback rule. “Singular final” indicates that the RI variance also approached zero; its point estimate and raw diagnostic quantities are retained in the machine-readable output, but its confidence interval, p, and q are not reported here. The remaining multilevel p values use large-sample Wald normal approximations. PISA student weights were used for within-economy preprocessing but were not passed to `lmer`, whose `weights` argument represents prior rather than survey-design weights. The multilevel models are therefore only model-based sensitivity analyses. Predictors that failed the stated availability criterion remain coverage-limited regardless of coefficient significance.
